# Variation along *P2RX7* interacts with early traumas on severity of anxiety suggesting a role for neuroinflammation

**DOI:** 10.1038/s41598-023-34781-w

**Published:** 2023-05-12

**Authors:** Zsuliet Kristof, Zsofia Gal, Dora Torok, Nora Eszlari, Sara Sutori, Berta Erdelyi-Hamza, Peter Petschner, Beata Sperlagh, Ian M. Anderson, John Francis William Deakin, Gyorgy Bagdy, Gabriella Juhasz, Xenia Gonda

**Affiliations:** 1 Doctoral School of Mental Health Sciences, Semmelweis University, Budapest, Hungary; 2 Laboratory of Molecular Pharmacology, Institute of Experimental Medicine, Budapest, Hungary; 3 Department of Psychiatry and Psychotherapy, Semmelweis University, Gyulai Pál Str. 2, Budapest, 1085 Hungary; 4 Department of Pharmacodynamics, Faculty of Pharmacy, Semmelweis University, Budapest, Hungary; 5 NAP3.0-SE Neuropsychopharmacology Research Group, Hungarian Brain Research Program, Semmelweis University, Budapest, Hungary; 6 Bioinformatics Center, Institute of Chemical Research, Kyoto University, Uji, Kyoto, Japan; 7 Research Unit for Realization of Sustainable Society, Kyoto University, Uji, Kyoto, Japan; 8 Division of Neuroscience and Experimental Psychology, School of Biological Sciences, Faculty of Biological, Medical and Human Sciences, Manchester Academic Health Sciences Centre, The University of Manchester, Manchester, UK

**Keywords:** Emotion, Neuroimmunology, Psychology, Human behaviour, Neuroscience, Genetics of the nervous system

## Abstract

Emotional stress is a leading risk factor in the development of neuropsychiatric disorders possibly via immune activation. P2X7 receptors promote neuroinflammation, and research suggests a relationship between chromosome region 12q2431, in which the *P2X7R* gene is located, and development of mood disorders, however, few studies concentrate on its association with anxiety. Our aim was to investigate the effects of *P2RX7* variation in interaction with early childhood traumas and recent stressors on anxiety. 1752 participants completed questionnaires assessing childhood adversities and recent negative life events, provided data on anxiety using the Brief Symptom Inventory, and were genotyped for 681 SNPs in the *P2RX7* gene, 335 of which passed quality control and were entered into linear regression models followed by a linkage disequilibrium-based clumping procedure to identify clumps of SNPs with a significant main or interaction effect. We identified a significant clump with top SNP rs67881993 and containing a set of 29SNPs that are in high LD, which significantly interacted with early childhood traumas but not with recent stress conveying a protective effect against increased anxiety in those exposed to early adversities. Our study demonstrated that *P2RX7* variants interact with distal and more etiological stressors in influencing the severity of anxiety symptoms, supporting previous scarce results and demonstrating its role in moderating the effects of stress.

## Introduction

Anxiety disorders are amongst the most prevalent and disabling psychiatric disorders, substantially impacting the quality of life. According to epidemiological surveys, up to 33.7% of the population is affected by at least one episode of an anxiety disorder during their lifetime^1^. Anxiety is also often present in various other psychiatric and also somatic disorders significantly decreasing quality of life, well-being and the course of these disorders. While anxiety has been extensively studied, its pathophysiology and genetic background is still not completely understood^2^ in part because anxiety disorders are highly multifaceted and heterogeneous. Current treatments for anxiety are similarly divergent and mostly involve medications primarily developed for other disease groups, and are only partially efficient. Thus, the identification of specific genetic and molecular targets for novel therapeutic approaches in the treatment of anxiety and anxiety disorders is urgent^3^. Furthermore, a paradigm shift is imminent, challenging the current “neuron-centric” approach to neuropsychiatric disorders, and embracing the concept that beyond neurons also their key cellular partners, glia are important players in the brain and may account for important aspects of psychiatric pathology^4–6^.

A growing body of evidence points to the crucial role of imbalance in the immune system in neuropsychiatric disorders leading to the hypothesis that certain inflammatory pathways contribute to the pathophysiology of major psychiatric illnesses^7^, including mood disorders, schizophrenia and anxiety disorders. The resident immune cells of the central nervous system (CNS), microglia are essential in maintaining innate immunity and are the drivers of inflammation within the CNS as well^6^. These macrophage cells are sensitive to environmental alterations in the brain and play an important role in modulating the impact of stress^8^. Stress exposure leads to the activation of the microglia, which results in the release of pro-inflammatory cytokines, including TNF-α and IL-1β, as well as other factors, such as glutamate, chemokines, nitric oxide (NO) and growth factors^4,9^. Several studies demonstrated an association between greater and/or perturbated microglial activity and different behavioural outcomes in animal models^10–14^.

The purinergic receptor P2X7 (P2X7R), a subtype of P2 purinoreceptor (P2R) family, is an ATP-gated nonselective cation channel, expressed in the brain primarily by microglia^14^. On the periphery, the primary role of P2X7R lies in the modulation of cytokine response to inflammatory challenge, while in the CNS, the best characterized consequence of P2X7R activation is the release of neurotransmitters, in particular, of glutamate to the extracellular space^15^. Increased glutamatergic neurotransmission has been implicated in several stress-related disorders, such as anxiety^16,17^ and depression^2,18,19^ that is supported by the significant anxiolytic-like effects of anti-glutamatergic compounds, like ketamine, observed in both animals and humans^20–22^. Moreover, the P2X7R antagonist Brillant Blue G is capable of alleviating the depression- and anxiety-like behaviours in epileptic rats, just as effectively as the classic anti-depressant and anti-anxiety drug fluoxetine^23^. Therefore, recent data indicate that the pharmacological blockade of P2 purinoreceptors could potentially elicit antidepressant- and/or anxiolytic-like effects^2^.

Several earlier candidate gene studies suggested a relationship between chromosome 12q2431, where *P2RX7* (Purinergic Receptor P2X 7 gene)is located, and the development of mood disorders^24–27^. Very recently, these earlier findings have been complemented by results from large-scale unbiased genomic studies, such as a genome-wide-Mendelian randomisation study which has strongly suggested P2RX7 as an actionable brain target for approved drugs or those in the clinical testing phase for depression^28^ also remarking that the *P2RX7* gene was one of the genes located in the genome-wide significant risk loci and also found as a risk gene in the original genome-wide association study (GWAS) in depression^29^. An even more recent cross-ancestry genome-wide association study (GWAS) with systems-level integrative analyses published as a preprint also implicated *P2RX7* as a causal variant in depression based on a colocalization analysis of QTL and GWAS signals and Mendelian randomization analyses (MR) once more strongly suggesting P2X7R as a promising drug target for the treatment of depression^30^.In a previous study in the same sample we found that while variation along the *P2RX7* shows no association with depressive symptom severity as a main effect, but moderates the effects of both early childhood traumas and recent life stressors on depression^31^. Anxiety and depression, however, in spite of a both being internalising disorders showing correlation in the clinical level, are clearly two distinct phenomena^32,33^ concerning their manifestations and related subjective experience, treatment, as well as their etiopathological pathways and biological and genetic underpinnings, with important differences in their adaptive functions, relationship with motivation and complex cognitive and regulatory processes, as well as in their heterogeneous nature^34,35^. Thus, it is of key importance to improve their differentiation and identify their distinctie aspects also on the neurobiolological and genetic level. Nevertheless, only a few studies specifically focus on the involvement of P2X7 receptor and the *P2RX7* gene in anxiety disorders and anxiety^36^, which thus warrants further clarification. While an increasing number of genome-wide association studies (GWAS) focus on psychiatric disorders or symptoms like anxiety, GWAS-s, due to the very large number of tests performed and stringent p-value criteria to compensate for this, may fail to detect existing associations, and SNPs with an effect not reaching genome-wide significance^37,38^. A possible approach to reducing multiple testing burden, while overcoming the hit-and-miss approach of candidate variant studies is employing a gene-wide approach focusing on all available SNPs along the *P2RX7* gene, and by “clumping” the variants inherited together based on linkage disequilibrium, as we did.

**Figure 1:**
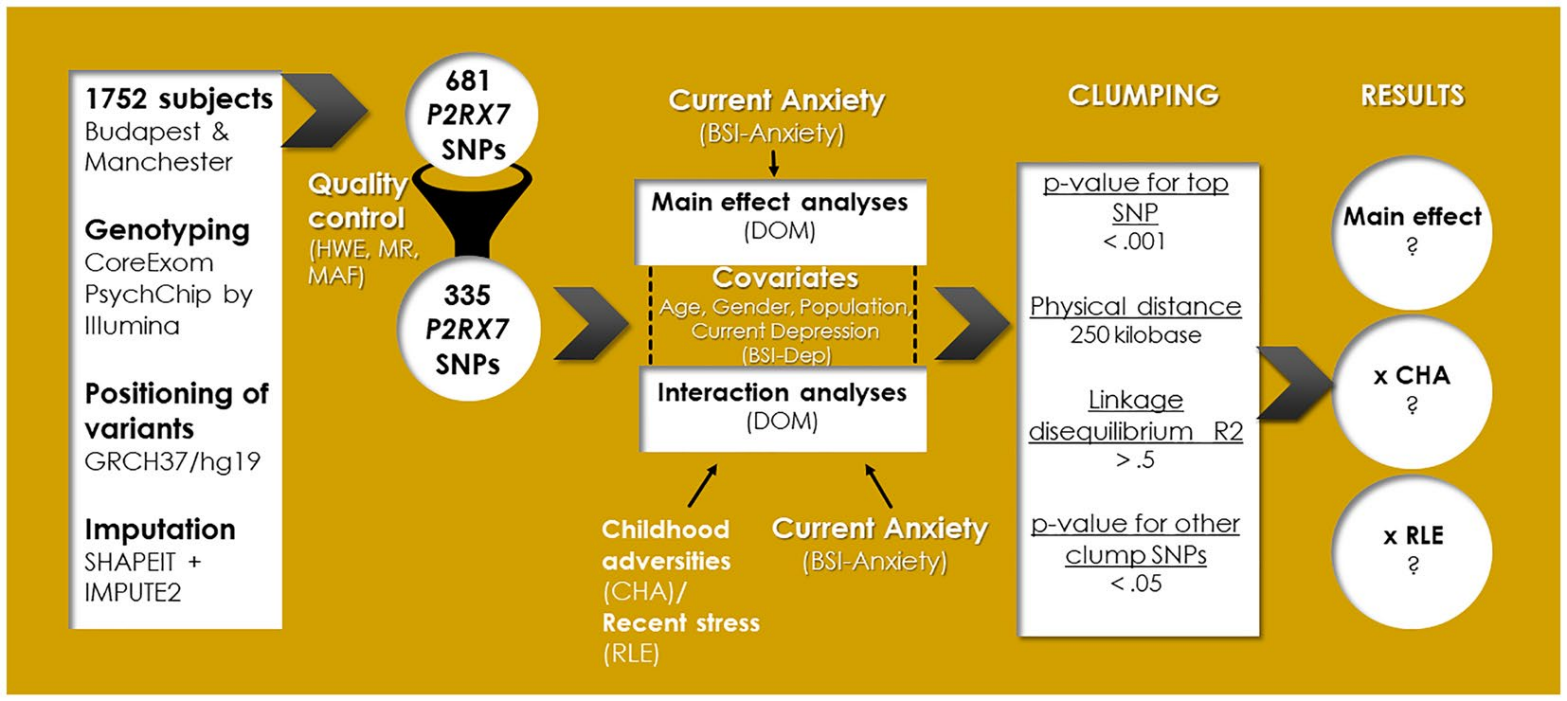
Aims and methods of investigating the effects of variation in *P2RX7* in interaction with childhood adversities and recent life events on current anxiety: study population, quality control steps and statistical analyses. *SNP* single nucleotide polymorphism, *HWE* Hardy–Weinberg equilibrium, *MR* missingness rate, *MAF* minor allele frequency, *BSI-anxiety* Brief Symptom Inventory anxiety score, *ADD* additive model, *DOM* dominant model, *REC* recessive model, *CHA* childhood adversities, *RLE* recent life events.

In our study, we focused on the effects of variation along the *P2RX7* gene in interaction with early childhood adversities and recent negative life events on current anxiety symptoms in a large general European sample, applying a linkage-disequilibrium-based clumping method (Fig. 1).

## Results

### Description of the sample

Descriptive statistics of the study sample are presented in Table 1.

**Table 1:** Descriptive statistics of the study sample.

Gender	n	%			
Male	501	28.6			
Female	1251	71.4			

	Minimum	Maximum	Mean	SEM	Range
Age	18	60	32.56	0.25	18–60
BSI-Anx	0	4	0.86	0.02	0–4
Childhood adversity	0	16	3.28	0.08	0–18
Recent negative life events	0	8	1.21	0.03	0–12

Anxiety symptom scores were measured by the Brief Symptom Inventory (BSI-Anx). Range: possible numeric range of the variable. Minimum and maximum refer to minimum and maximum values for the given variable in our sample.

*SEM* standard error of mean.

**Figure 2:**
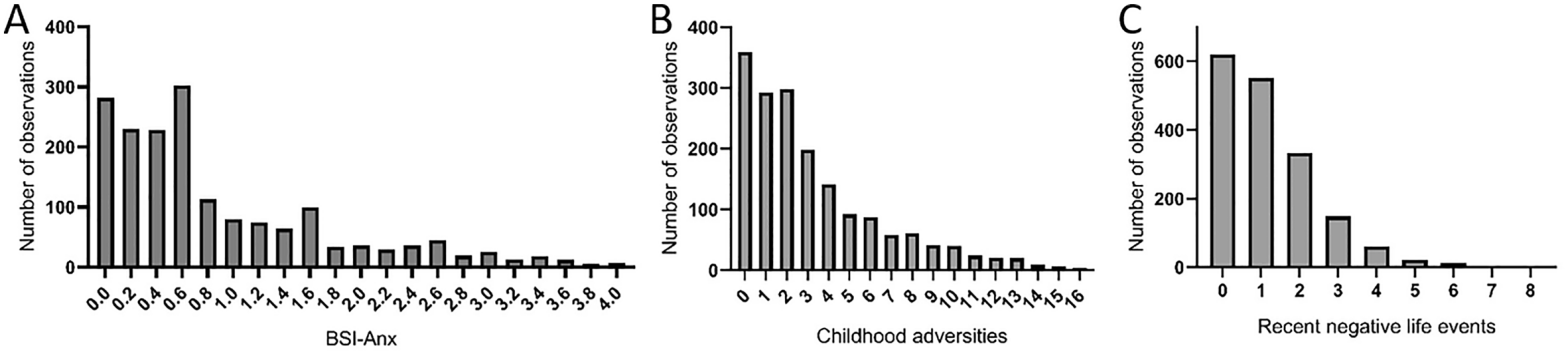
Distribution of anxiety scores (BSI-anx) (**A**); number of childhood adversities (**B**); and number recent negative life events (**C**) experienced in our sample.

Distribution of anxiety scores (BSI-Anx), number of childhood traumas (CHA), and number of recent life events (RLE) are shown in Fig. 2.

### Main effects of variation in *P2RX7* on current anxiety symptoms

Our linear regression models on current anxiety symptoms identified no SNPs with a nominally significant main effect, therefore clumps could not be formed.

### Gene x Environment effects of variation in *P2RX7* on current anxiety symptoms: interaction with childhood adversities (CHA)

**Figure 3:**
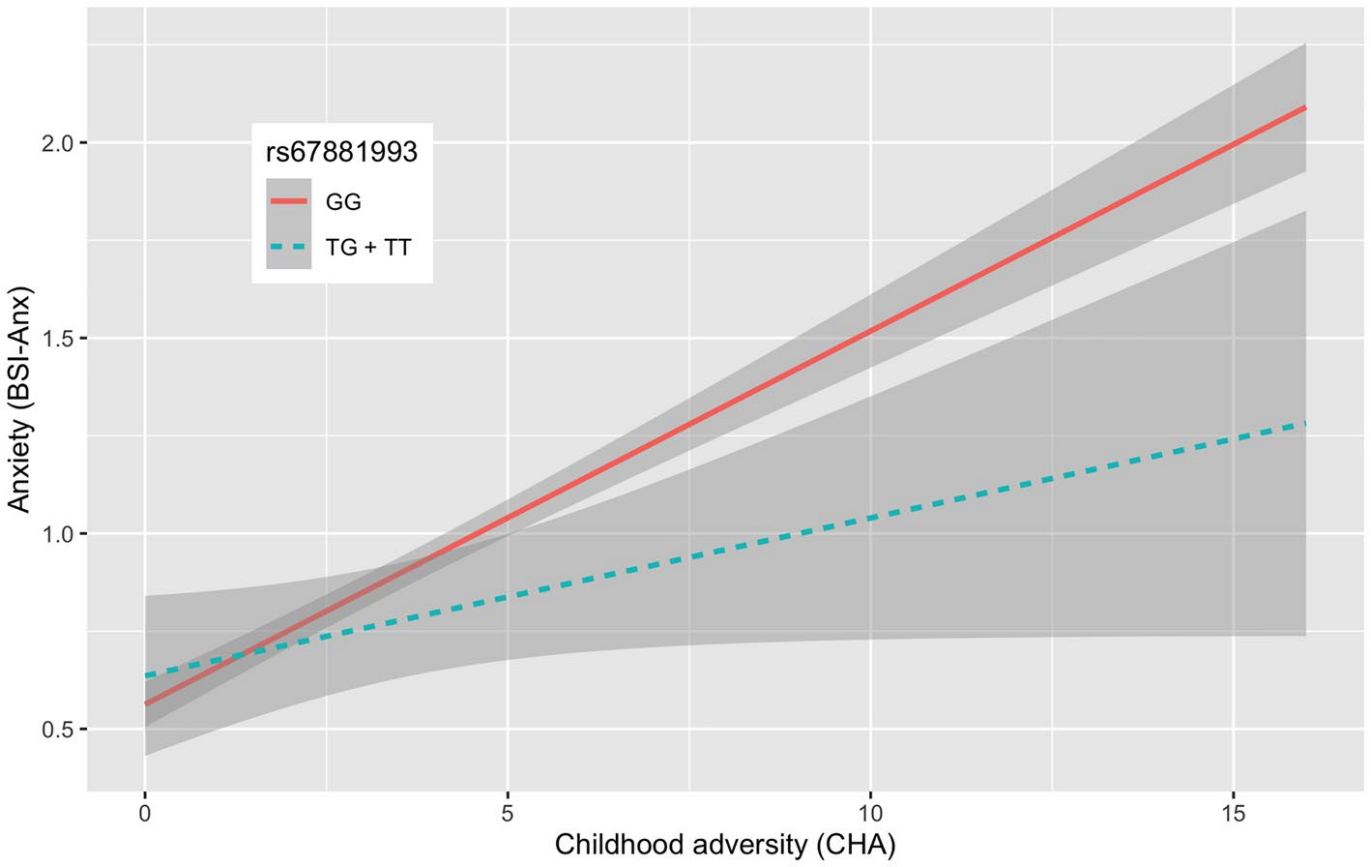
Linear regression analyses indicated a significant interaction between a clump comprising 29 SNPs in the *P2RX7* gene and exposure to childhood adversities on the severity of current anxiety symptoms, with the minor or effect alleles as a protective allele. For visualization purposes we show this effect for the top/index SNP rs67881993, with a protective effect for the minor T allele. Data on all *P2RX7* SNPs in the original NewMood database, including quality control and regression results in interaction with CHA for dominant models are shown in Supplementary Table S1.

**Figure 4:**
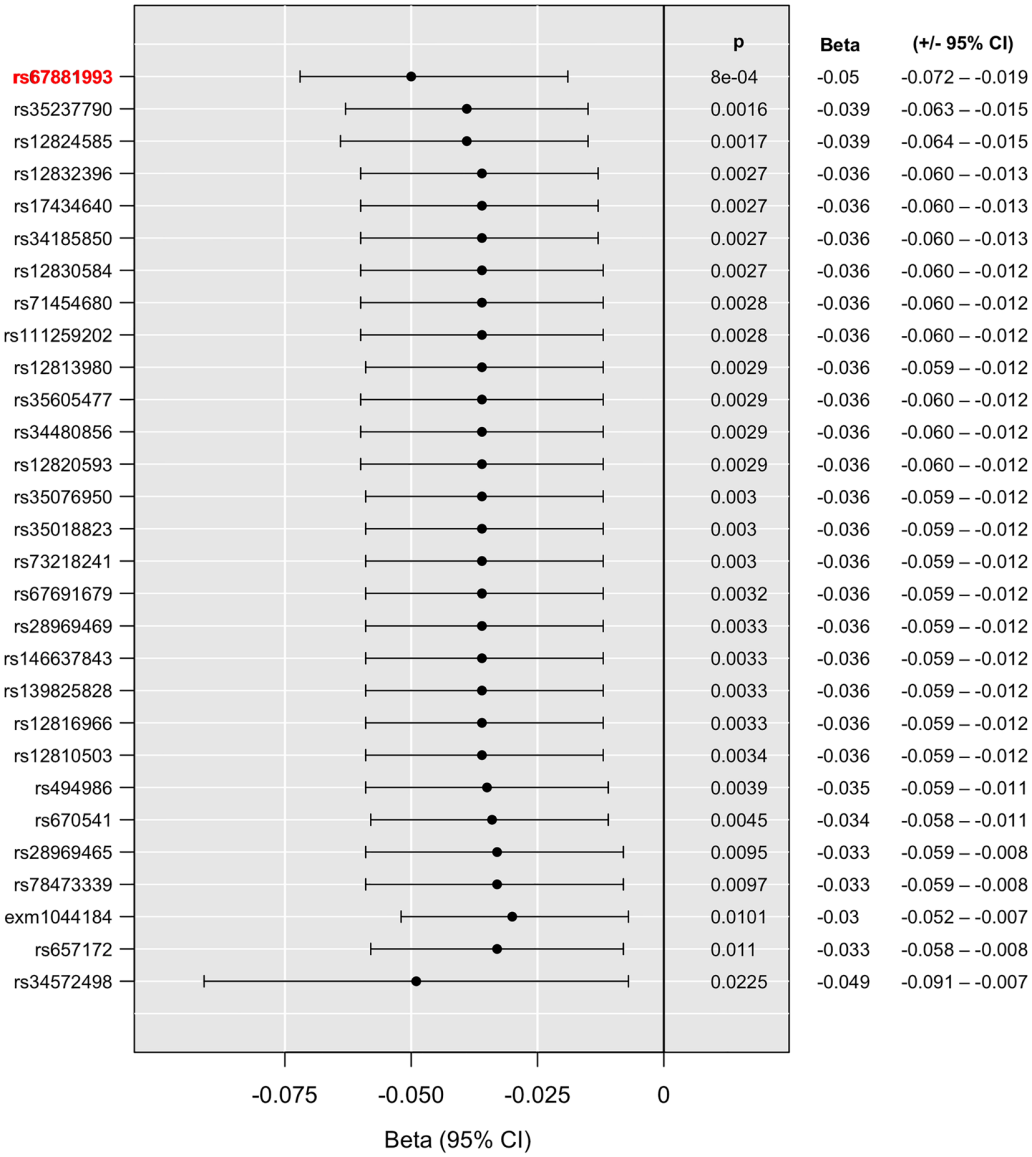
Significant clump of 29 SNPs interacting with early childhood adversities (CHA) on current anxiety symptoms. Linear regression beta coefficients and 95% CI values are shown in the figure. Bold red type denotes top SNPs of the clump. P denotes nominal significance, FDR Q shows levels of significance corrected for multiple testing.

Our analyses for interaction with childhood adversities on current anxiety symptoms yielded one significant clump including 29 SNPs in high LD surviving correction for multiple testing. The top SNP was rs67881993, where the minor T allele had a frequency of 0.0430. The presence of the minor or effect alleles of the SNPs comprising the clump was associated with a significantly lower increase in BSI-Anxiety sores compared to minor allele noncarriers if the subjects were exposed to more severe childhood traumas indicating a protective effect. (Fig. 3 shows the effect in case of the index SNP of the clump, rs67881993 for visualisation purposes, while Fig. 4 shows the effect of all variants on BSI-Anxiety in the interaction model.

### Gene × environment effects of variation in *P2RX7* on current anxiety symptoms: interaction with recent life events (RLE)

In case of gene × environment interaction models with recent life events on current anxiety symptoms, linear regression models yielded no significant clumps.

### In silico characterization and functional prediction of identified top SNP rs67881993

**Figure 5:**
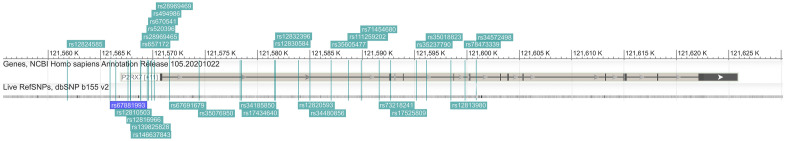
Genomic location of significant SNPs identified in the clumping procedure. Significant SNPs belonging to the clump identified in the analysis with an effect on current anxiety symptoms in interaction with childhood adversities are shown. Top SNP is highlighted in dark blue font UCSC Genome Browser on Human Feb. 2009 (GRCh37/hg19) was used to visualize the location of polymorphisms on *P2RX7* gene.

Genomic location of significant SNPs and top SNP identified in the clumping procedure are shown in Fig. 5.

Search in LitVar, dbSNP, UCSC Genome Browser and GWAS catalog database did not identify any relevant information regarding the identified SNPs in the significant clump (S2 Table).

**Figure 6:**
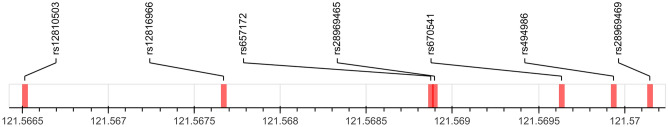
Significant SNPs interacting with childhood adversities on the severity of anxiety symptoms located in the transcription binding site of *P2RX7.*

To detect the functional effect of the significant SNPs, we utilized FuncPred (https://snpinfo.niehs.nih.gov), and SNPNexus (https://www.snp-nexus.org/v4/) web-based variant annotation tools (S2 Table). In silico functional characterization of lead SNP rs67881993 did not show any known functional effect. 7 SNPs (rs12810503, rs12816966, rs28969465, rs28969469, rs494986, rs657172, rs670541) are located in the transcription factor-binding site of the *P2RX7* gene, which suggest that this region possibly plays role in transcriptional regulatory processes (Fig. 6).

We also performed pathway analysis of the identified variants with SNPNexus. SNPNexus uses Reactome data to link genes involved in the observed variants. For each pathway, a p-value is provided, taking into account all the genes associated with the original SNP set. The identified pathways for our significant variants included (1) NLRP3 inflammasome R-HSA-844456 (p = 0.0013), (2) elevation of cytosolic Ca^2+^ levels, R-HSA-139853 (p = 0.0014), (3) inflammasomes, R-HSA-622312 (p = 0.0018), (4) purinergic signaling in leishmaniasis infection (p = 0.0022), (5) cell recruitment (pro-inflammatory response), R-HSA-9664424 (p = 0.0022), (6) platelet calcium homeostasis R-HSA-418360 (p = 0.0025), (7) nucleotide-binding domain, leucine rich repeat containing receptor (NLR) signaling pathways R-HSA-168643 (p = 0.0050).

## Discussion

Our present study investigated the effects of variation in the *P2RX7* gene on anxiety in a large European general population sample, using a clumping procedure investigating all available SNPs along the gene. We identified a significant clump comprised of a set of 29 SNPs in high linkage disequlibrium, with top SNP rs67881993, which significantly interacted with early childhood adverse experiences, conveying a protective effect, that is, leading to decreased anxiety levels in those minor allele carriers who reported having experienced more severe maltreatment in their childhood. We found no significant main effect, nor a significant interaction with recent negative life events for *P2RX7* gene on current anxiety. These findings underpin previous, although scarce results on the putative association of variation in this gene with anxiety, while providing a more robust support that it may play a role in moderating the effects of certain types of stress on the development of psychological symptoms.

### An emerging target: the potential role of neuroinflammation, P2X7 receptors and the *P2RX7* gene in the emergence of anxiety

In the context of the recent paradigm shift towards appreciation of the role of neuroinflammation in neuropsychiatric disorders and symptoms including anxiety, the P2X7 purine receptor is gaining significant interest as a possible central hub in brain disorders^36^.

P2X7 receptors play a role in processes related to neuroinflammatory response, impairment of neuroplasticity, and stimulation of glutamate response^36^, influencing cellular proliferation and death, rapid as well as reversible phosphatidylserine exposure, membrane blebbing, microparticle and exosome release, multinucleated cell formation, and reactive oxygen and nitrogen species production^36^. P2X7 receptors are implicated in various disorders including neurodegenerative as well as psychiatric disorders such as depression, bipolar disorder, schizophrenia, or anxiety. While P2X7 receptors may offer a potential target for prevention and intervention in various neuropsychiatric illnesses, study of their role and impact is hindered by the lack of specific agonists and sufficient antibodies^36^.

The *P2RX7* gene encoding the P2X7 receptor is located in humans at 12q24.31 chromosome position^39^, a region important for MDD, bipolar disorder, and anxiety disorders, suggesting a genetic overlap in these groups of psychiatric disorders^40^. Polymorphisms are widespread in the human *P2RX7* gene with at least a dozen non-synonymous polymorphisms (NS-SNPs) contributing to changes in amino acid sequence and as a consequence altered receptor function and possibly altered susceptibility to various neuropsychiatric diseases^41^. Preclinical studies combining rodent behaviour models with genetic and pharmacological interventions show the P2X7 receptor to be crucial in stress-induced depressive-like and anxiety-like behaviours. A number of human studies have observed significant association of SNPs and NS-SNPs with a vulnerability to mood disorders^42^.

### *P2RX7* variation does not directly influence severity of anxiety symptoms

However, to date, only very few studies investigating the role of the *P2RX7* gene in anxiety or anxiety disorders have been conducted, and even those focused primarily on other phenotypes, were carried out in small samples, and with a candidate variant approach focusing on a very small number of preselected polymorphisms, and while some of them reported a trend^40^ or a significant association^25^ of individual candidate variants with anxiety-related phenotypes, none of them considered the effect of stress. Our present analyses could not confirm a main effect of any SNPs in the *P2RX7* gene on current levels of anxiety thus arguing for the lack of direct involvement of this gene in anxious states and symptoms. Furthermore, in the same sample we also previously reported that variation in *P2RX7* does no directly impact current depressive symptoms, but only in interaction with early traumas or recent stressful life events^31^. Thus our present finding is comparable to our previous results suggesting that this gene is involved in moderating the effects of stress in the development of psychological symptoms rather then exerts a direct effect.

### P2X7 receptors interact with only certain types of stressors on current levels of anxiety

Psychosocial stress have been previously found to be associated with changes in ATP-mediated P2X7 receptor signalling and also neuroinflammation^43^. P2X7R has an abundant and widely accepted expression in microglia^44–46^, the immune cells of the brain, establishing P2X7R as a major driver of neuroinflammation, similar to its proinflammatory function in peripheral macrophages^47^. It is hypothesized that P2X7R is a silent receptor in nonpathological states and in the absence of ATP increase^48^. Stressful conditions, and especially chronic stress, however, lead to a sharp increase in extracellular ATP, acting as a damage-associated molecular pattern (DAMPs) leading to the activation of P2X7 receptors in microglia^49^, which in turn activates the NRLP3-associated inflammasome^50^ leading to increased release of proinflammatory cytokines^51,52^. In addition, P2X7R directly regulates hippocampal glutamate release and influences the signalling pathway of brain-derived neurotrophic factor (BDNF), which’s impairment is a vulnerability factor for anxiety disorders^53^. The above mechanism has been proposed to play a role in the effect of chronic stress leading to impaired neuroplasticity and the appearance of depressive-like behaviour, while blockade of the *P2RX7-*NLRP3-IL1β pathway may promote stress resilience via the involvement of microglia and monocytes^54^, thus supporting the possible role of P2X7R signalling as a connecting point between chronic stress and clinical mood symptoms^55,56^. As we mentioned before, only a very few studies, and usually not primarily focusing on anxiety, have reported results on the potential role of the *P2RX7* gene in anxiety, and none of them considered the possible interacting role of stress, although in one previous study rs208294 was found to be associated with mood disorder outcomes in a relationship mediated by neuroticism, a personality trait which is associated with less adaptive responses and increased anxiety upon exposure to stress^24^.

In our present study, considering the effects of both early and recent stress, we demonstrated that while *P2RX7* variation did not interact with recent, proximal stressors, one clump comprised of a set of 29 variants in high LD significantly interacted with early childhood traumas on current anxiety levels. This important finding is in line with rodent models investigating the developmental and long-term behavioural consequences of early life stress (ELS) and the potential long-term consequences of neuroinflammation in this relationship. Exposure to brief daily separation (BDS), a mouse model of ELS, induces clinical features seen in maltreated children including elevated markers of peripheral inflammation such as C-reactive protein and IL-6^57–60^, reduced myelination, and increased anxiety-like behaviours during the juvenile period and adulthood^61–64^. The low-grade inflammation mentioned above persists into adulthood; multiple studies report that inflammatory biomarkers are elevated in adults exposed to maltreatment and/or disadvantage during childhood^59,65,66^. These associations are typically independent of the adult’s concurrent psychosocial and socioeconomic conditions, suggesting that childhood adversity leaves an inflammatory “residue”^59^. Moreover, exposure to postnatal stress increases the density and activity of microglia in the hippocampus, which is associated with changes in the expression of several developmental genes involved in cell cycle progression, inflammation, and cell migration^61,67^. A large body of work has shown that the perturbation of microglial function during specific developmental periods causes structural alterations and behavioural changes in psychiatrically relevant behaviours such as anxiety, depression, and social affiliation that last into adulthood^61,68–71^. Our results, by showing a significant interaction of early stressors and a clump of variants in the *P2RX7* gene may suggest the involvement of altered P2X7R signalling in this process. Importantly, in our previous study in the same sample investigating the effect of *P2RX7* on depressive phenotypes we similarly found a significant interaction effect with early traumas^31^. However, the specific SNPs comprising the significantly interacting clumps are different for anxiety and depression, suggesting different biological mechanisms acting on *P2RX7* for anxiety and depression. Notably, the interaction between *P2RX7* variation and early stress on current anxiety was also significant while correcting our analysis of depression, supporting that this effect is valid and not simply a consequence of the overlap between depression and anxiety.

While the identified SNPs in the significant clump have not been reported before, seven of them are located in a transcription factor binding site of the *P2RX7* gene suggesting possible, although not yet characterized functional consequences. Furthermore, pathway analysis of the identified variants confirmed the involvement in inflammasome activation and cell recruitment during proinflammatory response.

### The potential role of the *P2RX7* gene in anxiety and depression

As mentioned above, we previously analysed the association between *P2RX7*, environmental stress, and depression in the same sample^31^, and to increase our chance to capture the association of *P2RX7* variation with only anxiety, we corrected for current depressive symptoms in our models. Earlier we found that while variation along the *P2RX7* gene had no direct main effect on depressive symptoms, it did moderate the effects of both early childhood traumas as well as recent life stressors on current depressive symptoms, although this latter effect was much weaker^31^. In case of anxiety, however, we found an effect only in case of early childhood traumas. This difference may support previous assumptions that different types of adverse environmental influences have different acute or long-term effects on psychopathology, and that distinct genes or variants may only moderate the effect of only specific types of stress^72^. While distal stressors such as early traumas in interaction with genetic risk variants may influence the development of a diathesis, proximal stressors such as recent life events may trigger the precipitation of symptoms underlied by this diathesis. Therefore it is of potential importance that our present findings concerning the association between *P2RX7* variation and anxiety show that, unlike in the case of depression, variation in this gene only moderates the effects of childhood traumas but not recent stressors on the appearance of the phenotype and symptoms, thus raising attention to the potentially different role of this gene in these two symptoms. Finally, considering that neuroinflammation is suspected in the background of both depressive and anxiety symptoms at least in a subgroup of patients, it is important to see how different genes, stressors and their interactions have a distinct and divergent impact on these phenomena. It is also noteworthy, that there is a significant clinical correlation between depression and anxiety as they are both internalising disorders responding to similar pharmacological and psychological treatments, which also show a genetic overlap in cross-disorder studies. However, given the differences in the subjective experience of patients in case of depression and anxiety both of which cause significant suffering, and the lack of suitable efficacy of currently available treatments, understanding both differences and similarities in their genetic and neurobiological backgrounds is of high clinical importance.

### Limitations

The present study has several limitations. First of all, we would like to mention once more that while we focused on anxiety in our present analysis, anxiety correlates with depression, and in fact anxiety is an integral part of depression and the most common mental disorder in the community is mixed anxiety-depression. However, in order to focus on anxiety, we corrected all our analyses for current depressive symptoms as measured by the BSI. Second, childhood adversity and recent life events were assessed retrospectively, and based on self-report of the subjects but not confirmed by other informants, which may contribute to distortion from several sources, including recall bias. Third, in a similar way, the severity of current anxiety was also based on a self-reported measure. Fourth, our sample consisted of approximately two thirds of female subjects and was limited to European white participants. Fifth, scoring childhood adversity and counting the number of recent negative life events does not take into consideration the differing severity of individual life events. Also, we considered objective occurrence of these life events and not their subjective meaning. As a final limitation it should be noted that the analyses were performed in a general population with low rates of anxiety, and early/late traumas. Nevertheless, our study also has several strengths, including considering several hundred variants along the *P2RX7* gene with a clump method rather than just a single candidate SNP, employing a dimensional approach capture current anxiety symptom severity, and using a GxE paradigm with two etiologically different types of stressors.

## Conclusions

In conclusion, our study is the first to investigate variation along the *P2RX7* gene in a large sample using a linkage disequilibrium-based clumping method and also a GxE interaction approach with two different types of stressors with respect to their effect on anxiety symptoms. We report that while variation in this gene does not directly influence anxiety, we also identified a clump of 29 variants in a high linkage disequilibrium, with a novel lead SNP moderating the effects of early traumas on the severity of current anxiety symptoms, and conveying a protective effect against higher anxiety for the minor/effect allele in those exposed to childhood trauma. Besides specifically focusing on the role of *P2RX7*, our findings extend our understanding of the role of neuroinflammation in anxiety and anxiety disorders, and also provide further support for the hypothesis that a great majority of genes involved in the background of anxiety act by influencing sensitivity to the deleterious effects of early, etiological stressors, and that effects of neuroinflammation stemming from early traumas and lasting into adulthood may have an important role in this relationship. Thus, understanding the role of *P2RX7* variation in anxiety also helps propose novel possible underlying pathways and mechanisms for the prevention and treatment of anxiety and anxiety disorders.

## Methods

### Study population

1752 unrelated volunteers (501 males, 1251 females) with self-reported European white ethnic origin aged 18–60 were recruited from the general population via advertisements, an online platform, and general practices to participate in the NewMood study (New Molecules in Mood Disorders, Sixth Framework Program of the European Union LHSM-CT-2004-503474) in Budapest and Greater Manchester. After obtaining written informed consent, participants provided genetic data by a saliva-based sampling kit for genotyping; and self-reported sociodemographic data including age, gender, recent negative life events occurring in the past year, childhood adversities, and current anxiety symptoms for phenotyping. A detailed description of the population sample is available in our previously published reports^73–75^. The study was carried out in accordance with the Declaration of Helsinki, and it was approved by the Scientific and Research Ethics Committee of the Medical Research Council, Budapest, Hungary, and by the North Manchester Local Research Ethics Committee, Manchester, United Kingdom. All participant provided written informed consent prior to participation.

### Phenotypes

The present study focused on measuring the current level of anxiety and two types of stressors.

#### Measurement of anxiety

Current level of anxiety (BSI-Anxiety) was assessed by the Brief Symptom Inventory^76^, a questionnaire measuring psychopathological symptoms in several scales, each item scored between 0 and 4 depending on the distress caused. For this study, we used only the anxiety subscale score to reflect the actual levels of anxiety, calculated as the sum of anxiety symptom item scores divided by the number of completed items. Anxiety showed a moderately strong correlation with the depression subscale of BSI (Spearman r = 0.7390, p < 0.0001). To focus on anxiety and separate it from overlapping depression symptoms, we also used the depression subscale of the Brief Symptom Inventory (depressive items plus 4 additional items, BSI-Depression) to adjust for current depressive symptoms in our statistical models.

#### Measures of environmental stressors

To evaluate gene-environment interactions contributing to the emergence of the measured anxiety symptoms, we assessed two types of environmental stressors: childhood adversities (CHA) as distal etiological stressors, and recent negative life events (RLE) occurring in the past year as proximal, trigger stressors. Early childhood adversity and trauma (CHA) was measured by an instrument derived from the Childhood Trauma Questionnaire (CTQ)^77^, including four items on emotional and physical abuse and emotional and physical neglect, and two items on the loss of parents, as validated previously^78^. The sum of item scores was used in the analyses. We utilized the List of Threatening Experiences^79,80^ to record recent stressful life events (RLE) occurring in the past year, comprised of 12 items evaluating four types of life events related to financial difficulties, illnesses/injuries, personal problems, and intimate relationship or social network difficulties. The number of recent negative life events was used in the statistical analyses. Anxiety scores showed a mild correlation with childhood adversity (r = 0.292, p < 0.05) and recent negative life event scores (r = 0.221, p < 0.05). There was also a very weak but significant correlation between childhood trauma scores and recent life event scores (r = 0.1196, p < 0.05). To correct for this, in the interaction analyses CHA and RLE were also covariants in the analyses which did not test for their interaction with *P2RX7* variation.

### Genotyping

Participants provided buccal mucosa cells collected by a cytology brush (Cytobrush plus C0012, Durbin PLC) to detect DNA. Genomic DNA extraction was carried out according to the protocol described by Freeman et al.^81^. Genotyping was performed by Illumina's CoreExom PsychChip. All laboratory work was performed under the ISO 9001:2000 quality management requirements, and was blinded regarding phenotype. Variant annotation was carried out based on the GRCh37/hg19 human assembly. For the purpose of phasing SHAPEIT was used to estimate additional haplotypes, followed by imputation of missing genotypes via IMPUTE2.

### Statistical analyses

Genotyping and imputation provided a dataset incorporating 1752 individuals and 681 SNPs in the region of *P2RX7* gene (with boundaries extended by 10 kb) available in the NewMood database. We applied a 3-step quality control protocol for the SNPs, involving the calculation of Hardy–Weinberg Equilibrium (HWE; > 1 × 10^−5^), missingness rates (MR; < 0.05), and minor allele frequencies (MAF; > 0.01), which was survived by 335 SNPs. These SNPs were analysed with linear regression models to test for main effects of *P2RX7* variants on current anxiety, and gene-environment interaction models with early childhood adversities and recent negative life events were run as well (Fig. 1).

Next, the regression models were followed by a clumping procedure both for main effect and for the two types GxE interaction effects separately, based on linkage disequilibrium (LD) estimates between the SNPs using the CLUMP function in Plink. Clumping is a statistical method for yielding clumps of intercorrelated SNPs based on empirical estimates of their linkage disequilibrium (LD), stepping beyond independent significance levels, identifying connected SNPs and their top SNP, with the highest significance. The four parameters used for clumping were the following: (1) maximum p-value of the clump's top SNP was 0.001; (2) maximum p-value for secondary SNPs was 0.05; (3) minimum LD R2 was 0.5; and (4) physical distance threshold was 250 kilobases.

In the above analyses, Plink v1.90 was used to calculate MR (< 0.05), HWE (> 1 × 10^−5^) and MAF (> 0.01) as part of quality control steps prior to the analyses; for clumping; and for building linear regression models to test for main and interaction effects of genetic variation in the *P2RX7* gene. Analyses were supported by scripts individually written in R 3.0.2 (R Core Team, 2013). Descriptive statistics were calculated using IBM SPSS Statistics 25. For visualization purposes the R software (version R-4.1.1) and Rstudio (version 2021.9.0.351) were used, supported by the Ggplot2 (Wickham, 2016) package^82^.

All analyses were run according to dominant models, as small MAF values in case of several SNPs (however, still meeting MAF requirements as part of our quality control) would have led to groups carrying the minor allele with very small sample sizes. In all Plink linear regression models population (to account for rectruitment sites, as a categorical variable), gender (as a categorical variable), age, (as a dimensional variable) and BSI-depression (as a dimensional variable) were covariates. Additionally, when testing an SNP × CHA/RLE interaction effect, main effects of both the SNP and CHA/RLE were also entered as covariates in the model. Nominal significance threshold was p < 0.05. To correct for multiple comparisons in analyses for each of the above outcome variables, Benjamini–Hochberg false discovery rate (FDR) Q-values were calculated; results with a Q-value of ≤ 0.05 were considered significant. The design and methods of our study are shown in Fig. 1.

## Supplementary Information

Supplementary Tables.

## Data Availability

The datasets presented in this study can be found in online FigShare repository at https://figshare.com/articles/dataset/Effect_of_P2RX7_variation_and_stress_on_anxiety/20278905 (10.6084/m9.figshare.20278905.v1).
