# Supplementary material for: Variation along P2RX7 interacts with early traumas on severity of anxiety suggesting a role for neuroinflammation
Source: Sci Rep. 2023 May 12;13:7757. doi: 10.1038/s41598-023-34781-w (PMC10182087; doi:10.1038/s41598-023-34781-w)
Supplement: Supplementary file 1 — Supplementary Tables. [file 41598_2023_34781_MOESM1_ESM.docx]

Variation along P2RX7 interacts with early traumas on severity of anxiety suggesting a role for neuroinflammation

Zsuliet Kristof, Zsofia Gal, Dora Torok, Nora Eszlari, Sara Sutori, Berta Erdelyi-Hamza, Peter Petschner, Beata Sperlagh, Ian M. Anderson, John Francis William Deakin, Gyorgy Bagdy, Gabriella Juhasz, Xenia Gonda

S1 Table. P2RX7 SNPs in the NewMood database in interaction with childhood adversities (CHA) with results in quality control steps and linear regression for BSI-Anxiety

S2 Table. In silico functional characterisation of SNPs in the significant clumps

| S1 Table. P2RX7 SNPs in the NewMood database in interaction with childhood adversities (CHA) with results in quality control steps and linear regression for BSI-Anxiety | | | | | | | | | | | | | |
| --- | --- | --- | --- | --- | --- | --- | --- | --- | --- | --- | --- | --- | --- |
| CHR | SNP | BP | A1 | TEST | BETA | SE | L95 | U95 | STAT | P | Hardy | MAF | Missing |
| 12 | **rs67881993:121565950:G:T** | **121565950** | **T** | **DOMxCHS** | **-0.04518** | **0.01348** | **-0.0716** | **-0.01876** | **-3.351** | **0.000822** | **0.2349** | **0.04195** | **0.02418** |
| 12 | exm1044272 | 121622304 | C | DOMxCHS | -0.02645 | 0.00807 | -0.04226 | -0.01063 | -3.277 | 0.001069 | 0.7644 | 0.1942 | 0 |
| 12 | rs35237790:121595186:A:G | 121595186 | G | DOMxCHS | -0.03889 | 0.01233 | -0.06305 | -0.01473 | -3.155 | 0.001631 | 0.2569 | 0.05537 | 0.007692 |
| 12 | rs181687754:121606765:T:C | 121606765 | C | DOMxCHS | -0.2533 | 0.08041 | -0.4109 | -0.09574 | -3.151 | 0.001658 | 1 | 0.001126 | 0.02418 |
| 12 | rs12824585:121561868:A:C | 121561868 | C | DOMxCHS | -0.03914 | 0.01244 | -0.06353 | -0.01475 | -3.145 | 0.001688 | 0.3383 | 0.05279 | 0.01648 |
| 12 | rs11065475:121630438:A:G | 121630438 | G | DOMxCHS | 0.02644 | 0.00851 | 0.009762 | 0.04312 | 3.107 | 0.001921 | 0.2117 | 0.1584 | 0.03077 |
| 12 | psy_rs11615992 | 121627268 | G | DOMxCHS | -0.0257 | 0.008287 | -0.04195 | -0.00946 | -3.101 | 0.001958 | 0.8735 | 0.1794 | 0 |
| 12 | rs34185850:121578351:T:C | 121578351 | C | DOMxCHS | -0.03604 | 0.012 | -0.05956 | -0.01253 | -3.004 | 0.002701 | 0.2812 | 0.05855 | 0.00055 |
| 12 | rs17434640:121578450:G:A | 121578450 | A | DOMxCHS | -0.03604 | 0.012 | -0.05956 | -0.01253 | -3.004 | 0.002701 | 0.2812 | 0.05855 | 0.00055 |
| 12 | rs71454679:121580223:G:A | 121580223 | A | DOMxCHS | -0.03604 | 0.012 | -0.05956 | -0.01253 | -3.004 | 0.002701 | 0.2812 | 0.05855 | 0.00055 |
| 12 | rs12832396:121581681:T:C | 121581681 | C | DOMxCHS | -0.03602 | 0.012 | -0.05953 | -0.01251 | -3.003 | 0.002715 | 0.2858 | 0.05913 | 0.001099 |
| 12 | rs12830584:121581661:A:T | 121581661 | T | DOMxCHS | -0.036 | 0.012 | -0.05952 | -0.01248 | -3 | 0.002737 | 0.2813 | 0.05858 | 0.001099 |
| 12 | rs111259202:121588707:G:A | 121588707 | A | DOMxCHS | -0.03595 | 0.012 | -0.05947 | -0.01243 | -2.996 | 0.002777 | 0.2834 | 0.05886 | 0.001099 |
| 12 | rs71454680:121589925:G:A | 121589925 | A | DOMxCHS | -0.03591 | 0.01201 | -0.05946 | -0.01237 | -2.99 | 0.00283 | 0.2813 | 0.05858 | 0.001099 |
| 12 | rs12820593:121583937:A:G | 121583937 | G | DOMxCHS | -0.03585 | 0.01201 | -0.05939 | -0.0123 | -2.984 | 0.002883 | 0.2812 | 0.05855 | 0.00055 |
| 12 | rs34480856:121584990:G:A | 121584990 | A | DOMxCHS | -0.03585 | 0.01201 | -0.05939 | -0.0123 | -2.984 | 0.002883 | 0.2812 | 0.05855 | 0.00055 |
| 12 | rs35605477:121587034:C:T | 121587034 | T | DOMxCHS | -0.03585 | 0.01201 | -0.05939 | -0.0123 | -2.984 | 0.002883 | 0.2812 | 0.05855 | 0.00055 |
| 12 | rs12813980:121598487:C:A | 121598487 | A | DOMxCHS | -0.03578 | 0.01201 | -0.05932 | -0.01224 | -2.98 | 0.002927 | 0.2834 | 0.05886 | 0.001099 |
| 12 | rs73218241:121591632:G:A | 121591632 | A | DOMxCHS | -0.03572 | 0.01201 | -0.05925 | -0.01218 | -2.974 | 0.002976 | 0.2833 | 0.05882 | 0.00055 |
| 12 | rs35018823:121596139:G:A | 121596139 | A | DOMxCHS | -0.03572 | 0.01201 | -0.05925 | -0.01218 | -2.974 | 0.002976 | 0.2833 | 0.05882 | 0.00055 |
| 12 | rs35076950:121574410:C:T | 121574410 | T | DOMxCHS | -0.03586 | 0.01208 | -0.05952 | -0.01219 | -2.969 | 0.003026 | 0.3797 | 0.05736 | 0.003846 |
| 12 | rs67691679:121571646:C:T | 121571646 | T | DOMxCHS | -0.03568 | 0.01208 | -0.05936 | -0.012 | -2.953 | 0.003188 | 0.3774 | 0.05718 | 0.005495 |
| 12 | rs208289:121593381:A:G | 121593381 | G | DOMxCHS | 0.02916 | 0.009876 | 0.0098 | 0.04851 | 2.952 | 0.003221 | 0.6664 | 0.2208 | 0.3753 |
| 12 | rs12816966:121567670:A:G | 121567670 | G | DOMxCHS | -0.03555 | 0.01208 | -0.05923 | -0.01186 | -2.941 | 0.00331 | 0.3751 | 0.05697 | 0.006593 |
| 12 | rs139825828:121567913:A:T | 121567913 | T | DOMxCHS | -0.03555 | 0.01208 | -0.05923 | -0.01186 | -2.941 | 0.00331 | 0.3751 | 0.05697 | 0.006593 |
| 12 | rs146637843:121567915:G:C | 121567915 | C | DOMxCHS | -0.03555 | 0.01208 | -0.05923 | -0.01186 | -2.941 | 0.00331 | 0.3751 | 0.05697 | 0.006593 |
| 12 | rs28969469:121570147:A:G | 121570147 | G | DOMxCHS | -0.03555 | 0.01208 | -0.05923 | -0.01186 | -2.941 | 0.00331 | 0.3749 | 0.05694 | 0.006044 |
| 12 | rs12810503:121566515:G:C | 121566515 | C | DOMxCHS | -0.0356 | 0.01213 | -0.05936 | -0.01183 | -2.936 | 0.003369 | 0.3731 | 0.05679 | 0.008242 |
| 12 | rs494986:121569936:A:C | 121569936 | A | DOMxCHS | -0.03495 | 0.01209 | -0.05864 | -0.01125 | -2.891 | 0.00389 | 0.378 | 0.05731 | 0.007692 |
| 12 | rs12372746:121628052:A:G | 121628052 | G | DOMxCHS | 0.02428 | 0.008424 | 0.007772 | 0.04079 | 2.883 | 0.003994 | 0.3538 | 0.1694 | 0.02198 |
| 12 | rs11065474:121630348:A:G | 121630348 | G | DOMxCHS | 0.02313 | 0.0081 | 0.007259 | 0.03901 | 2.856 | 0.004342 | 0.8324 | 0.2162 | 0.04066 |
| 12 | rs670541:121569634:T:C | 121569634 | T | DOMxCHS | -0.03411 | 0.012 | -0.05764 | -0.01059 | -2.842 | 0.004529 | 0.5273 | 0.06013 | 0.01319 |
| 12 | rs11065473:121628709:G:T | 121628709 | T | DOMxCHS | 0.02225 | 0.008392 | 0.005802 | 0.0387 | 2.651 | 0.008091 | 0.3102 | 0.1687 | 0.02143 |
| 12 | rs144782841:121633581:C:G | 121633581 | G | DOMxCHS | 0.02484 | 0.009396 | 0.00642 | 0.04325 | 2.643 | 0.008288 | 0.04108 | 0.1185 | 0.02198 |
| 12 | rs180753544:121564797:G:A | 121564797 | A | DOMxCHS | 0.2235 | 0.08464 | 0.05763 | 0.3894 | 2.641 | 0.008344 | 1 | 0.001652 | 0.002198 |
| 12 | rs78595509:121595453:T:C | 121595453 | C | DOMxCHS | 0.08268 | 0.03148 | 0.02099 | 0.1444 | 2.627 | 0.008698 | 1 | 0.01277 | 0.01044 |
| 12 | rs28969465:121568896:A:G | 121568896 | G | DOMxCHS | -0.03338 | 0.01285 | -0.05857 | -0.0082 | -2.598 | 0.009465 | 0.4561 | 0.0507 | 0.01923 |
| 12 | rs78473339:121599829:G:C | 121599829 | C | DOMxCHS | -0.03324 | 0.01283 | -0.05838 | -0.0081 | -2.591 | 0.00965 | 0.487 | 0.05463 | 0.01429 |
| 12 | exm1044184 | 121592689 | G | DOMxCHS | -0.02953 | 0.01147 | -0.05201 | -0.00705 | -2.574 | 0.01012 | 0.8539 | 0.06868 | 0 |
| 12 | rs657172:121568874:G:A | 121568874 | G | DOMxCHS | -0.03272 | 0.01286 | -0.05791 | -0.00752 | -2.545 | 0.01101 | 0.4603 | 0.05104 | 0.02033 |
| 12 | rs208302:121602760:G:A | 121602760 | A | DOMxCHS | 0.02036 | 0.008016 | 0.004652 | 0.03607 | 2.54 | 0.01116 | 0.8933 | 0.2304 | 0.02802 |
| 12 | rs208295:121600779:A:G | 121600779 | G | DOMxCHS | 0.02865 | 0.01146 | 0.006179 | 0.05111 | 2.499 | 0.01256 | 0.6988 | 0.06962 | 0.1121 |
| 12 | rs183949627:121622625:C:A | 121622625 | A | DOMxCHS | 0.1002 | 0.04313 | 0.01566 | 0.1847 | 2.323 | 0.0203 | 1 | 0.002501 | 0.01154 |
| 12 | rs34572498:121600811:C:G | 121600811 | G | DOMxCHS | -0.04946 | 0.02166 | -0.09192 | -0.00701 | -2.283 | 0.02253 | 0.3992 | 0.01698 | 0.02912 |
| 12 | rs140031782:121604726:G:A | 121604726 | A | DOMxCHS | 0.04095 | 0.01812 | 0.005433 | 0.07646 | 2.26 | 0.02396 | 0.6358 | 0.0269 | 0.02967 |
| 12 | exm1044264 | 121622196 | G | DOMxCHS | 0.01842 | 0.00815 | 0.002441 | 0.03439 | 2.259 | 0.02398 | 0.6857 | 0.1758 | 0 |
| 12 | rs75268007:121627748:C:G | 121627748 | G | DOMxCHS | 0.02062 | 0.009178 | 0.002629 | 0.0386 | 2.246 | 0.02481 | 0.07741 | 0.1315 | 0.01813 |
| 12 | rs1653625:121622885:C:A | 121622885 | A | DOMxCHS | 0.0203 | 0.009177 | 0.002314 | 0.03829 | 2.212 | 0.02714 | 1 | 0.2837 | 0.2824 |
| 12 | rs146942410:121589443:G:A | 121589443 | A | DOMxCHS | -0.1052 | 0.04776 | -0.1988 | -0.01161 | -2.203 | 0.02773 | 1 | 0.004178 | 0.01374 |
| 12 | 12:121562259:C:T | 121562259 | T | DOMxCHS | -0.0925 | 0.04226 | -0.1753 | -0.00967 | -2.189 | 0.02875 | 1 | 0.003062 | 0.01319 |
| 12 | rs12366304:121615629:C:G | 121615629 | G | DOMxCHS | 0.01764 | 0.00818 | 0.00161 | 0.03368 | 2.157 | 0.03116 | 0.5681 | 0.1746 | 0.003846 |
| 12 | rs1975124:121628224:G:T | 121628224 | T | DOMxCHS | 0.01789 | 0.008387 | 0.001455 | 0.03433 | 2.133 | 0.03303 | 0.01437 | 0.4017 | 0.04725 |
| 12 | rs1653609:121605919:A:C | 121605919 | C | DOMxCHS | 0.0183 | 0.008749 | 0.001155 | 0.03545 | 2.092 | 0.03659 | 0.6715 | 0.4878 | 0.008791 |
| 12 | rs10849852:121631969:T:G | 121631969 | G | DOMxCHS | 0.02045 | 0.009796 | 0.00125 | 0.03965 | 2.088 | 0.03699 | 0.8119 | 0.1122 | 0.02802 |
| 12 | rs1653604:121611201:T:C | 121611201 | C | DOMxCHS | 0.01804 | 0.008692 | 0.001 | 0.03507 | 2.075 | 0.03814 | 0.6722 | 0.4862 | 0.004396 |
| 12 | rs12316059:121627464:T:A | 121627464 | A | DOMxCHS | 0.02274 | 0.01133 | 0.000537 | 0.04495 | 2.007 | 0.04487 | 0.7272 | 0.07311 | 0.01923 |
| 12 | rs3886929:121593600:A:C | 121593600 | C | DOMxCHS | 0.02229 | 0.01113 | 0.000471 | 0.0441 | 2.002 | 0.04542 | 0.6047 | 0.0757 | 0.06374 |
| 12 | rs2116499:121584160:A:G | 121584160 | G | DOMxCHS | -0.01984 | 0.00996 | -0.03936 | -0.00032 | -1.992 | 0.04654 | 0.7946 | 0.1002 | 0.004945 |
| 12 | rs1136293:121629119:G:A | 121629119 | A | DOMxCHS | 0.02252 | 0.01131 | 0.000353 | 0.0447 | 1.991 | 0.04663 | 0.7284 | 0.07403 | 0.02033 |
| 12 | rs7132846:121614934:C:T | 121614934 | T | DOMxCHS | 0.02192 | 0.01117 | 2.51E-05 | 0.04382 | 1.962 | 0.0499 | 0.8649 | 0.07394 | 0.00055 |
| 12 | rs58423269:121612364:T:A | 121612364 | A | DOMxCHS | 0.02186 | 0.01118 | -4.82E-05 | 0.04376 | 1.956 | 0.05067 | 1 | 0.07343 | 0.001099 |
| 12 | rs3751146:121613090:G:A | 121613090 | A | DOMxCHS | 0.02185 | 0.01118 | -6.67E-05 | 0.04376 | 1.954 | 0.05086 | 1 | 0.07347 | 0.001648 |
| 12 | rs3751142:121622419:G:T | 121622419 | T | DOMxCHS | 0.02157 | 0.01115 | -0.00029 | 0.04342 | 1.934 | 0.0533 | 0.8649 | 0.07422 | 0.00055 |
| 12 | rs7312642:121620187:G:T | 121620187 | T | DOMxCHS | 0.02155 | 0.01115 | -0.0003 | 0.0434 | 1.933 | 0.05343 | 0.8649 | 0.07418 | 0 |
| 12 | rs3751144 | 121622239 | A | DOMxCHS | 0.02155 | 0.01115 | -0.0003 | 0.0434 | 1.933 | 0.05343 | 0.8649 | 0.07418 | 0 |
| 12 | rs7305883:121608775:G:A | 121608775 | A | DOMxCHS | 0.02155 | 0.01115 | -0.00031 | 0.04341 | 1.932 | 0.0535 | 0.8649 | 0.07422 | 0.00055 |
| 12 | exm1044253 | 121615131 | C | DOMxCHS | 0.02151 | 0.01116 | -0.00036 | 0.04337 | 1.928 | 0.05405 | 0.8649 | 0.07394 | 0.00055 |
| 12 | rs3751147:121613060:G:C | 121613060 | C | DOMxCHS | 0.02148 | 0.01115 | -0.00038 | 0.04334 | 1.926 | 0.05425 | 0.8649 | 0.07426 | 0.001099 |
| 12 | rs117692465:121579140:G:A | 121579140 | A | DOMxCHS | -0.03688 | 0.01937 | -0.07485 | 0.001094 | -1.904 | 0.05715 | 1 | 0.026 | 0.08077 |
| 12 | 12:121589624:T:C | 121589624 | C | DOMxCHS | 0.0538 | 0.02849 | -0.00204 | 0.1096 | 1.888 | 0.05914 | 1 | 0.005257 | 0.007143 |
| 12 | rs520396:121569505:G:T | 121569505 | G | DOMxCHS | -0.01893 | 0.01005 | -0.03863 | 0.000773 | -1.883 | 0.05985 | 1 | 0.09794 | 0.01264 |
| 12 | rs61953399:121614248:C:T | 121614248 | T | DOMxCHS | 0.01556 | 0.008308 | -0.00073 | 0.03184 | 1.873 | 0.0613 | 0.6204 | 0.1721 | 0.007143 |
| 12 | rs368489709:121602470:G:A | 121602470 | A | DOMxCHS | 0.7131 | 0.3821 | -0.03584 | 1.462 | 1.866 | 0.06219 | 1 | 0.00055 | 0.00055 |
| 12 | rs189490384:121568358:C:T | 121568358 | T | DOMxCHS | -0.1278 | 0.06963 | -0.2643 | 0.008659 | -1.836 | 0.06659 | 1 | 0.00055 | 0.001648 |
| 12 | rs12312648:121564391:C:T | 121564391 | T | DOMxCHS | -0.01745 | 0.009511 | -0.03609 | 0.001193 | -1.835 | 0.06675 | 0.816 | 0.1147 | 0.02253 |
| 12 | rs12312695:121564463:C:T | 121564463 | T | DOMxCHS | -0.01745 | 0.009511 | -0.03609 | 0.001193 | -1.835 | 0.06675 | 0.816 | 0.1147 | 0.02253 |
| 12 | rs12312758:121564615:C:A | 121564615 | A | DOMxCHS | -0.01745 | 0.009511 | -0.03609 | 0.001193 | -1.835 | 0.06675 | 0.816 | 0.1147 | 0.02253 |
| 12 | rs9805004:121565870:C:T | 121565870 | T | DOMxCHS | -0.01745 | 0.009511 | -0.03609 | 0.001193 | -1.835 | 0.06675 | 0.816 | 0.1147 | 0.02253 |
| 12 | rs2708099:121563794:G:C | 121563794 | C | DOMxCHS | 0.03397 | 0.01852 | -0.00233 | 0.07027 | 1.834 | 0.06683 | 1 | 0.01942 | 0.00989 |
| 12 | rs12310654:121563293:C:T | 121563293 | T | DOMxCHS | -0.01695 | 0.009406 | -0.03539 | 0.001486 | -1.802 | 0.07172 | 0.816 | 0.1151 | 0.02143 |
| 12 | rs12297280:121563386:A:T | 121563386 | T | DOMxCHS | -0.01695 | 0.009406 | -0.03539 | 0.001486 | -1.802 | 0.07172 | 0.816 | 0.1151 | 0.02143 |
| 12 | rs35694882:121563709:G:A | 121563709 | A | DOMxCHS | -0.01695 | 0.009406 | -0.03539 | 0.001486 | -1.802 | 0.07172 | 0.816 | 0.1151 | 0.02143 |
| 12 | rs71454678:121563923:G:A | 121563923 | A | DOMxCHS | -0.01695 | 0.009406 | -0.03539 | 0.001486 | -1.802 | 0.07172 | 0.816 | 0.1151 | 0.02143 |
| 12 | rs12821512:121564398:T:C | 121564398 | C | DOMxCHS | -0.01695 | 0.009406 | -0.03539 | 0.001486 | -1.802 | 0.07172 | 0.8159 | 0.1149 | 0.02198 |

| 12 | rs11065444:121564917:T:C | 121564917 | C | DOMxCHS | -0.01695 | 0.009406 | -0.03539 | 0.001486 | -1.802 | 0.07172 | 0.816 | 0.1151 | 0.02143 |
| --- | --- | --- | --- | --- | --- | --- | --- | --- | --- | --- | --- | --- | --- |
| 12 | rs12314721:121565757:C:T | 121565757 | T | DOMxCHS | -0.01695 | 0.009406 | -0.03539 | 0.001486 | -1.802 | 0.07172 | 0.8159 | 0.1149 | 0.02198 |
| 12 | rs79623919:121615641:C:T | 121615641 | T | DOMxCHS | 0.01388 | 0.00774 | -0.00129 | 0.02905 | 1.793 | 0.07311 | 0.1151 | 0.2631 | 0.006044 |
| 12 | rs139282112:121612661:G:A | 121612661 | A | DOMxCHS | 0.07443 | 0.04177 | -0.00743 | 0.1563 | 1.782 | 0.07491 | 1 | 0.003874 | 0.007143 |
| 12 | rs55868376:121563155:A:T | 121563155 | T | DOMxCHS | -0.0167 | 0.009389 | -0.0351 | 0.001705 | -1.778 | 0.07551 | 0.6474 | 0.1172 | 0.02527 |
| 12 | rs74982443:121606005:T:C | 121606005 | C | DOMxCHS | 0.5846 | 0.3301 | -0.06239 | 1.232 | 1.771 | 0.07674 | 1 | 0.000828 | 0.004945 |
| 12 | rs7137542:121612646:G:T | 121612646 | T | DOMxCHS | 0.5846 | 0.3301 | -0.06239 | 1.232 | 1.771 | 0.07674 | 1 | 0.000828 | 0.004945 |
| 12 | rs1718160:121611350:T:C | 121611350 | C | DOMxCHS | 0.01427 | 0.008186 | -0.00178 | 0.03032 | 1.743 | 0.08148 | 0.01998 | 0.4107 | 0.002747 |
| 12 | rs141555626:121630339:G:A | 121630339 | A | DOMxCHS | -0.1212 | 0.07048 | -0.2594 | 0.01689 | -1.72 | 0.08555 | 1 | 0.00339 | 0.02747 |
| 12 | rs12830607:121562482:A:G | 121562482 | G | DOMxCHS | -0.01603 | 0.009325 | -0.03431 | 0.002246 | -1.719 | 0.08578 | 0.7339 | 0.1182 | 0.01923 |
| 12 | rs150561863:121628339:G:A | 121628339 | A | DOMxCHS | -0.1276 | 0.07447 | -0.2736 | 0.01831 | -1.714 | 0.0867 | 1 | 0.001393 | 0.01374 |
| 12 | rs2686384:121625091:G:A | 121625091 | A | DOMxCHS | 0.01384 | 0.008222 | -0.00227 | 0.02996 | 1.684 | 0.09243 | 0.02232 | 0.4091 | 0.006044 |
| 12 | rs2568003:121621817:A:G | 121621817 | G | DOMxCHS | 0.01306 | 0.007761 | -0.00215 | 0.02828 | 1.683 | 0.09254 | 0.667 | 0.3228 | 0.006044 |
| 12 | rs1718108:121618665:G:A | 121618665 | A | DOMxCHS | 0.01368 | 0.00821 | -0.00242 | 0.02977 | 1.666 | 0.09592 | 0.02272 | 0.4111 | 0.001648 |
| 12 | rs2915641:121620766:A:G | 121620766 | G | DOMxCHS | 0.01366 | 0.008215 | -0.00244 | 0.02977 | 1.663 | 0.09643 | 0.02004 | 0.4113 | 0.002198 |
| 12 | rs2567986:121614352:A:G | 121614352 | G | DOMxCHS | 0.01362 | 0.008209 | -0.00247 | 0.02971 | 1.66 | 0.09715 | 0.01994 | 0.4108 | 0.002198 |
| 12 | rs1653598:121615283:T:C | 121615283 | C | DOMxCHS | 0.0136 | 0.008209 | -0.00249 | 0.02969 | 1.657 | 0.09769 | 0.02007 | 0.4112 | 0.00055 |
| 12 | rs11065468:121615369:T:C | 121615369 | C | DOMxCHS | 0.0136 | 0.008209 | -0.00249 | 0.02969 | 1.657 | 0.09769 | 0.02007 | 0.4112 | 0.00055 |
| 12 | rs7137047:121615786:G:C | 121615786 | C | DOMxCHS | 0.0136 | 0.008209 | -0.00249 | 0.02969 | 1.657 | 0.09769 | 0.02007 | 0.4112 | 0.00055 |
| 12 | rs11065469:121616456:G:A | 121616456 | A | DOMxCHS | 0.0136 | 0.008209 | -0.00249 | 0.02969 | 1.657 | 0.09769 | 0.02007 | 0.4112 | 0.00055 |
| 12 | rs11065470:121616467:T:C | 121616467 | C | DOMxCHS | 0.0136 | 0.008209 | -0.00249 | 0.02969 | 1.657 | 0.09769 | 0.02007 | 0.4112 | 0.00055 |
| 12 | rs2567997:121617272:C:T | 121617272 | T | DOMxCHS | 0.0136 | 0.008209 | -0.00249 | 0.02969 | 1.657 | 0.09769 | 0.02007 | 0.4112 | 0.00055 |
| 12 | rs35734276:121617386:T:C | 121617386 | C | DOMxCHS | 0.0136 | 0.008209 | -0.00249 | 0.02969 | 1.657 | 0.09769 | 0.02007 | 0.4112 | 0.00055 |
| 12 | rs2686380:121617707:C:T | 121617707 | T | DOMxCHS | 0.0136 | 0.008209 | -0.00249 | 0.02969 | 1.657 | 0.09769 | 0.02007 | 0.4112 | 0.00055 |
| 12 | rs891781:121618438:C:T | 121618438 | T | DOMxCHS | 0.0136 | 0.008209 | -0.00249 | 0.02969 | 1.657 | 0.09769 | 0.02007 | 0.4112 | 0.00055 |
| 12 | rs1718107:121618762:A:G | 121618762 | G | DOMxCHS | 0.0136 | 0.008209 | -0.00249 | 0.02969 | 1.657 | 0.09769 | 0.02007 | 0.4112 | 0.00055 |
| 12 | rs1653610:121606405:G:A | 121606405 | A | DOMxCHS | 0.01367 | 0.008253 | -0.0025 | 0.02985 | 1.657 | 0.0977 | 0.01998 | 0.4116 | 0.002747 |
| 12 | rs1653617:121608680:C:G | 121608680 | G | DOMxCHS | 0.01367 | 0.008253 | -0.0025 | 0.02985 | 1.657 | 0.0977 | 0.01998 | 0.4116 | 0.002747 |
| 12 | rs2567999:121619521:A:G | 121619521 | G | DOMxCHS | 0.0136 | 0.00821 | -0.00249 | 0.02969 | 1.657 | 0.09775 | 0.01757 | 0.4109 | 0.001099 |
| 12 | rs2568001:121619974:A:C | 121619974 | C | DOMxCHS | 0.0136 | 0.00821 | -0.00249 | 0.02969 | 1.657 | 0.09775 | 0.01757 | 0.4109 | 0.001099 |
| 12 | rs11065471:121621245:G:A | 121621245 | A | DOMxCHS | 0.0136 | 0.00821 | -0.00249 | 0.02969 | 1.657 | 0.09775 | 0.01757 | 0.4109 | 0.001099 |
| 12 | rs1718130:121605954:G:A | 121605954 | A | DOMxCHS | 0.01356 | 0.008204 | -0.00252 | 0.02964 | 1.653 | 0.09847 | 0.01761 | 0.4117 | 0.001648 |
| 12 | rs2567991:121610489:A:G | 121610489 | G | DOMxCHS | 0.01355 | 0.008207 | -0.00254 | 0.02964 | 1.651 | 0.09894 | 0.01757 | 0.4109 | 0.001099 |
| 12 | rs1653605:121611055:A:C | 121611055 | C | DOMxCHS | 0.01355 | 0.008207 | -0.00254 | 0.02964 | 1.651 | 0.09894 | 0.01757 | 0.4109 | 0.001099 |
| 12 | rs1653603:121611281:A:G | 121611281 | G | DOMxCHS | 0.01355 | 0.008207 | -0.00254 | 0.02964 | 1.651 | 0.09894 | 0.01757 | 0.4109 | 0.001099 |
| 12 | rs1653602:121611635:A:G | 121611635 | G | DOMxCHS | 0.01355 | 0.008207 | -0.00254 | 0.02964 | 1.651 | 0.09894 | 0.01757 | 0.4109 | 0.001099 |
| 12 | rs2686375:121611929:T:C | 121611929 | C | DOMxCHS | 0.01355 | 0.008207 | -0.00254 | 0.02964 | 1.651 | 0.09894 | 0.01757 | 0.4109 | 0.001099 |
| 12 | rs1653601:121613695:T:C | 121613695 | C | DOMxCHS | 0.01355 | 0.008207 | -0.00254 | 0.02964 | 1.651 | 0.09894 | 0.01757 | 0.4109 | 0.001099 |
| 12 | rs2686376:121614337:T:C | 121614337 | C | DOMxCHS | 0.01355 | 0.008207 | -0.00254 | 0.02964 | 1.651 | 0.09894 | 0.01757 | 0.4109 | 0.001099 |
| 12 | rs2686377:121614338:G:A | 121614338 | A | DOMxCHS | 0.01355 | 0.008207 | -0.00254 | 0.02964 | 1.651 | 0.09894 | 0.01757 | 0.4109 | 0.001099 |
| 12 | rs2686378:121614536:G:A | 121614536 | A | DOMxCHS | 0.01355 | 0.008207 | -0.00254 | 0.02964 | 1.651 | 0.09894 | 0.01757 | 0.4109 | 0.001099 |
| 12 | rs2686379:121617260:G:A | 121617260 | A | DOMxCHS | 0.01354 | 0.008209 | -0.00255 | 0.02963 | 1.649 | 0.09931 | 0.01757 | 0.4109 | 0.001099 |
| 12 | rs2567985:121614534:A:G | 121614534 | G | DOMxCHS | 0.01352 | 0.008213 | -0.00258 | 0.02961 | 1.646 | 0.1 | 0.01747 | 0.4105 | 0.002198 |
| 12 | rs641940:121567912:C:T | 121567912 | C | DOMxCHS | -0.01412 | 0.008635 | -0.03105 | 0.0028 | -1.636 | 0.1021 | 0.7032 | 0.145 | 0.01319 |
| 12 | rs1169738:121584410:G:A | 121584410 | A | DOMxCHS | 0.02854 | 0.01745 | -0.00566 | 0.06273 | 1.636 | 0.1021 | 1 | 0.02241 | 0.007143 |
| 12 | rs2567978:121631065:G:T | 121631065 | T | DOMxCHS | 0.01343 | 0.008248 | -0.00274 | 0.0296 | 1.628 | 0.1037 | 0.9488 | 0.2555 | 0.08077 |
| 12 | rs2859393:121596959:C:T | 121596959 | T | DOMxCHS | 0.02822 | 0.01742 | -0.00593 | 0.06237 | 1.619 | 0.1055 | 1 | 0.02244 | 0.008242 |
| 12 | rs1618709:121606917:C:T | 121606917 | T | DOMxCHS | 0.01316 | 0.008201 | -0.00292 | 0.02923 | 1.605 | 0.1088 | 0.0228 | 0.4107 | 0 |
| 12 | rs1626329:121622023:C:T | 121622023 | T | DOMxCHS | 0.01303 | 0.0082 | -0.00304 | 0.0291 | 1.589 | 0.1122 | 0.0134 | 0.4103 | 0.001648 |
| 12 | rs1653611:121606760:A:G | 121606760 | G | DOMxCHS | 0.01303 | 0.008211 | -0.00306 | 0.02912 | 1.587 | 0.1127 | 0.02014 | 0.4128 | 0.001648 |
| 12 | rs2857590:121606059:C:A | 121606059 | A | DOMxCHS | 0.01297 | 0.008203 | -0.00311 | 0.02905 | 1.581 | 0.1139 | 0.01769 | 0.4113 | 0 |
| 12 | rs1718131:121606319:G:A | 121606319 | A | DOMxCHS | 0.01297 | 0.008203 | -0.00311 | 0.02905 | 1.581 | 0.1139 | 0.01769 | 0.4113 | 0 |
| 12 | rs1718132:121606350:G:T | 121606350 | T | DOMxCHS | 0.01297 | 0.008203 | -0.00311 | 0.02905 | 1.581 | 0.1139 | 0.01769 | 0.4113 | 0 |
| 12 | rs1619628:121607052:A:G | 121607052 | G | DOMxCHS | 0.01297 | 0.008203 | -0.00311 | 0.02905 | 1.581 | 0.1139 | 0.01769 | 0.4113 | 0 |
| 12 | rs1653612:121607056:C:T | 121607056 | T | DOMxCHS | 0.01297 | 0.008203 | -0.00311 | 0.02905 | 1.581 | 0.1139 | 0.01769 | 0.4113 | 0 |
| 12 | rs2857591:121607340:C:T | 121607340 | T | DOMxCHS | 0.01297 | 0.008203 | -0.00311 | 0.02905 | 1.581 | 0.1139 | 0.01769 | 0.4113 | 0 |
| 12 | rs2857592:121607344:A:G | 121607344 | G | DOMxCHS | 0.01297 | 0.008203 | -0.00311 | 0.02905 | 1.581 | 0.1139 | 0.01769 | 0.4113 | 0 |
| 12 | rs1653614:121607795:A:G | 121607795 | G | DOMxCHS | 0.01297 | 0.008203 | -0.00311 | 0.02905 | 1.581 | 0.1139 | 0.01769 | 0.4113 | 0 |
| 12 | rs1718133:121607861:G:A | 121607861 | A | DOMxCHS | 0.01297 | 0.008203 | -0.00311 | 0.02905 | 1.581 | 0.1139 | 0.01769 | 0.4113 | 0 |
| 12 | rs1718135:121607987:G:A | 121607987 | A | DOMxCHS | 0.01297 | 0.008203 | -0.00311 | 0.02905 | 1.581 | 0.1139 | 0.01769 | 0.4113 | 0 |
| 12 | rs2567988:121608273:C:T | 121608273 | T | DOMxCHS | 0.01297 | 0.008203 | -0.00311 | 0.02905 | 1.581 | 0.1139 | 0.02021 | 0.4115 | 0 |
| 12 | rs1653616:121608556:G:A | 121608556 | A | DOMxCHS | 0.01297 | 0.008203 | -0.00311 | 0.02905 | 1.581 | 0.1139 | 0.01769 | 0.4113 | 0 |
| 12 | rs1653618:121608856:C:T | 121608856 | T | DOMxCHS | 0.01297 | 0.008203 | -0.00311 | 0.02905 | 1.581 | 0.1139 | 0.01769 | 0.4113 | 0 |
| 12 | rs2567989:121608908:G:A | 121608908 | A | DOMxCHS | 0.01297 | 0.008203 | -0.00311 | 0.02905 | 1.581 | 0.1139 | 0.01769 | 0.4113 | 0 |
| 12 | rs1718136 | 121609608 | G | DOMxCHS | 0.01297 | 0.008203 | -0.00311 | 0.02905 | 1.581 | 0.1139 | 0.02021 | 0.4115 | 0 |
| 12 | rs1718137:121609813:C:T | 121609813 | T | DOMxCHS | 0.01297 | 0.008203 | -0.00311 | 0.02905 | 1.581 | 0.1139 | 0.01769 | 0.4113 | 0 |
| 12 | rs2686373:121609944:G:A | 121609944 | A | DOMxCHS | 0.01297 | 0.008203 | -0.00311 | 0.02905 | 1.581 | 0.1139 | 0.01769 | 0.4113 | 0 |
| 12 | rs2686374:121609953:T:C | 121609953 | C | DOMxCHS | 0.01297 | 0.008203 | -0.00311 | 0.02905 | 1.581 | 0.1139 | 0.01769 | 0.4113 | 0 |
| 12 | rs1621388:121622563:G:A | 121622563 | A | DOMxCHS | 0.01297 | 0.008203 | -0.00311 | 0.02905 | 1.581 | 0.1139 | 0.01546 | 0.411 | 0 |
| 12 | rs2568002:121621737:A:G | 121621737 | G | DOMxCHS | 0.01297 | 0.008205 | -0.00311 | 0.02906 | 1.581 | 0.114 | 0.0155 | 0.4109 | 0.00055 |
| 12 | 12:121598964:T:G | 121598964 | G | DOMxCHS | 0.6026 | 0.3823 | -0.1466 | 1.352 | 1.576 | 0.1151 | 1 | 0.000551 | 0.002747 |
| 12 | rs3751148:121612941:C:T | 121612941 | T | DOMxCHS | 0.07294 | 0.0467 | -0.01858 | 0.1645 | 1.562 | 0.1185 | 1 | 0.00509 | 0.02857 |
| 12 | rs504677:121605189:C:T | 121605189 | T | DOMxCHS | 0.01322 | 0.008475 | -0.0034 | 0.02983 | 1.559 | 0.1191 | 0.07564 | 0.4183 | 0.0478 |
| 12 | rs368882722:121624545:C:T | 121624545 | T | DOMxCHS | -0.3953 | 0.255 | -0.8951 | 0.1044 | -1.551 | 0.1212 | 1 | 0.00055 | 0.001099 |
| 12 | rs376814011:121607463:C:T | 121607463 | T | DOMxCHS | -0.3926 | 0.2545 | -0.8915 | 0.1063 | -1.543 | 0.1231 | 1 | 0.000553 | 0.007143 |
| 12 | exm1044252 | 121615103 | A | DOMxCHS | 0.01258 | 0.008205 | -0.0035 | 0.02866 | 1.533 | 0.1254 | 0.01179 | 0.411 | 0 |
| 12 | rs147220840:121620102:A:G | 121620102 | G | DOMxCHS | 0.1764 | 0.1154 | -0.04968 | 0.4025 | 1.529 | 0.1264 | 1 | 0.001101 | 0.002198 |
| 12 | rs208298:121602238:G:A | 121602238 | A | DOMxCHS | 0.01191 | 0.007814 | -0.0034 | 0.02723 | 1.525 | 0.1275 | 0.2771 | 0.3189 | 0.01538 |
| 12 | rs208299:121602381:C:T | 121602381 | T | DOMxCHS | 0.01181 | 0.007808 | -0.00349 | 0.02711 | 1.513 | 0.1305 | 0.3621 | 0.3235 | 0.003846 |
| 12 | rs208300:121602420:C:T | 121602420 | T | DOMxCHS | 0.01181 | 0.007808 | -0.00349 | 0.02711 | 1.513 | 0.1305 | 0.3621 | 0.3235 | 0.003846 |
| 12 | rs208301:121602534:A:G | 121602534 | G | DOMxCHS | 0.01181 | 0.007808 | -0.00349 | 0.02711 | 1.513 | 0.1305 | 0.3621 | 0.3235 | 0.003846 |
| 12 | rs208303:121602825:A:G | 121602825 | G | DOMxCHS | 0.01181 | 0.007808 | -0.00349 | 0.02711 | 1.513 | 0.1305 | 0.3621 | 0.3235 | 0.003846 |
| 12 | rs208304:121603053:T:C | 121603053 | C | DOMxCHS | 0.01181 | 0.007808 | -0.00349 | 0.02711 | 1.513 | 0.1305 | 0.3621 | 0.3235 | 0.003846 |
| 12 | rs507085:121603118:T:C | 121603118 | C | DOMxCHS | 0.01181 | 0.007808 | -0.00349 | 0.02711 | 1.513 | 0.1305 | 0.3621 | 0.3235 | 0.003846 |
| 12 | rs654856:121603122:C:A | 121603122 | A | DOMxCHS | 0.01181 | 0.007808 | -0.00349 | 0.02711 | 1.513 | 0.1305 | 0.3621 | 0.3235 | 0.003846 |
| 12 | rs208305:121603469:A:C | 121603469 | C | DOMxCHS | 0.01181 | 0.007808 | -0.00349 | 0.02711 | 1.513 | 0.1305 | 0.3621 | 0.3235 | 0.003846 |

| 12 | rs208306:121603745:T:G | 121603745 | G | DOMxCHS | 0.01181 | 0.007808 | -0.00349 | 0.02711 | 1.513 | 0.1305 | 0.3621 | 0.3235 | 0.003846 |
| --- | --- | --- | --- | --- | --- | --- | --- | --- | --- | --- | --- | --- | --- |
| 12 | rs208307:121603856:C:G | 121603856 | G | DOMxCHS | 0.01181 | 0.007808 | -0.00349 | 0.02711 | 1.513 | 0.1305 | 0.3621 | 0.3235 | 0.003846 |
| 12 | rs208308:121604126:A:G | 121604126 | G | DOMxCHS | 0.01181 | 0.007808 | -0.00349 | 0.02711 | 1.513 | 0.1305 | 0.3621 | 0.3235 | 0.003846 |
| 12 | rs208309:121604135:A:T | 121604135 | T | DOMxCHS | 0.01181 | 0.007808 | -0.00349 | 0.02711 | 1.513 | 0.1305 | 0.3621 | 0.3235 | 0.003846 |
| 12 | rs78930509:121604147:A:C | 121604147 | C | DOMxCHS | 0.01181 | 0.007808 | -0.00349 | 0.02711 | 1.513 | 0.1305 | 0.3621 | 0.3235 | 0.003846 |
| 12 | rs208310:121604250:G:C | 121604250 | C | DOMxCHS | 0.01181 | 0.007808 | -0.00349 | 0.02711 | 1.513 | 0.1305 | 0.3621 | 0.3235 | 0.003846 |
| 12 | rs190833971:121588752:A:G | 121588752 | G | DOMxCHS | -0.09961 | 0.06598 | -0.2289 | 0.02971 | -1.51 | 0.1313 | 1 | 0.001925 | 0.001099 |
| 12 | rs61953398:121610161:G:A | 121610161 | A | DOMxCHS | 0.02171 | 0.01447 | -0.00665 | 0.05006 | 1.5 | 0.1337 | 0.72 | 0.03335 | 0.01154 |
| 12 | rs2567979:121631100:G:T | 121631100 | T | DOMxCHS | 0.01271 | 0.00855 | -0.00405 | 0.02947 | 1.486 | 0.1374 | 0.6744 | 0.1712 | 0.03407 |
| 12 | rs208311 | 121604314 | G | DOMxCHS | 0.01157 | 0.007796 | -0.00372 | 0.02685 | 1.483 | 0.1381 | 0.3922 | 0.3245 | 0 |
| 12 | rs17435031:121600465:T:G | 121600465 | G | DOMxCHS | 0.01929 | 0.01306 | -0.00631 | 0.04488 | 1.477 | 0.14 | 1 | 0.04706 | 0.03681 |
| 12 | rs11615889:121609733:C:T | 121609733 | T | DOMxCHS | -0.02984 | 0.02035 | -0.06973 | 0.01005 | -1.466 | 0.1428 | 0.5958 | 0.0227 | 0.04396 |
| 12 | rs189002082:121630265:A:T | 121630265 | T | DOMxCHS | -1.114 | 0.7652 | -2.614 | 0.3857 | -1.456 | 0.1456 | 1 | 0.000553 | 0.007143 |
| 12 | rs6489794:121597413:G:A | 121597413 | A | DOMxCHS | 0.0133 | 0.009189 | -0.00471 | 0.03131 | 1.448 | 0.1478 | 0.4062 | 0.1316 | 0.02088 |
| 12 | rs531095:121605756:T:C | 121605756 | C | DOMxCHS | 0.01186 | 0.008222 | -0.00426 | 0.02797 | 1.442 | 0.1495 | 0.02543 | 0.412 | 0.007692 |
| 12 | rs117509023:121593002:A:G | 121593002 | G | DOMxCHS | -0.05605 | 0.03926 | -0.133 | 0.02091 | -1.428 | 0.1536 | 1 | 0.002211 | 0.006044 |
| 12 | rs1169715:121578332:G:A | 121578332 | A | DOMxCHS | 0.02574 | 0.0181 | -0.00973 | 0.06122 | 1.422 | 0.1552 | 1 | 0.02009 | 0.001648 |
| 12 | rs112831493:121586173:G:T | 121586173 | T | DOMxCHS | 0.05273 | 0.0374 | -0.02056 | 0.126 | 1.41 | 0.1587 | 1 | 0.002217 | 0.008791 |
| 12 | rs138403654:121562772:G:A | 121562772 | A | DOMxCHS | -0.1247 | 0.08929 | -0.2997 | 0.05025 | -1.397 | 0.1626 | 1 | 0.001384 | 0.007692 |
| 12 | rs192293736:121563446:G:A | 121563446 | A | DOMxCHS | -0.1247 | 0.08929 | -0.2997 | 0.05025 | -1.397 | 0.1626 | 1 | 0.001384 | 0.007692 |
| 12 | rs140940661:121602817:A:G | 121602817 | G | DOMxCHS | -0.1243 | 0.09029 | -0.3013 | 0.05267 | -1.377 | 0.1688 | 1 | 0.001101 | 0.001648 |
| 12 | rs151191599:121610077:A:T | 121610077 | T | DOMxCHS | -0.1243 | 0.09029 | -0.3013 | 0.05267 | -1.377 | 0.1688 | 1 | 0.001101 | 0.001648 |
| 12 | rs118002597:121625260:C:T | 121625260 | T | DOMxCHS | 0.06529 | 0.0489 | -0.03057 | 0.1611 | 1.335 | 0.1821 | 1 | 0.004151 | 0.007143 |
| 12 | rs657995:121576992:G:C | 121576992 | C | DOMxCHS | 0.01396 | 0.01053 | -0.00668 | 0.03461 | 1.326 | 0.1851 | 0.8762 | 0.08287 | 0.01209 |
| 12 | rs672140:121577847:T:C | 121577847 | C | DOMxCHS | 0.01396 | 0.01053 | -0.00668 | 0.03461 | 1.326 | 0.1851 | 0.8762 | 0.08287 | 0.01209 |
| 12 | rs28969479:121622641:G:A | 121622641 | A | DOMxCHS | 0.05114 | 0.03873 | -0.02477 | 0.127 | 1.32 | 0.1869 | 1 | 0.003615 | 0.01209 |
| 12 | exm1044289 | 121622550 | A | DOMxCHS | -0.03945 | 0.03011 | -0.09846 | 0.01955 | -1.31 | 0.1902 | 1 | 0.004396 | 0 |
| 12 | 12:121600618:C:T | 121600618 | T | DOMxCHS | -0.09251 | 0.0707 | -0.2311 | 0.04607 | -1.308 | 0.1909 | 1 | 0.001932 | 0.004396 |
| 12 | rs199691547:121603840:T:C | 121603840 | C | DOMxCHS | 0.0127 | 0.009712 | -0.00634 | 0.03173 | 1.307 | 0.1913 | 0.05564 | 0.1752 | 0.2802 |
| 12 | rs686638:121605599:C:T | 121605599 | T | DOMxCHS | 0.01038 | 0.007982 | -0.00527 | 0.02602 | 1.3 | 0.1939 | 0.6711 | 0.343 | 0.04121 |
| 12 | rs12829218:121618391:A:G | 121618391 | G | DOMxCHS | -0.01893 | 0.01469 | -0.04772 | 0.009854 | -1.289 | 0.1976 | 0.1861 | 0.0385 | 0.008242 |
| 12 | rs669547:121577284:T:C | 121577284 | C | DOMxCHS | 0.01336 | 0.01037 | -0.00696 | 0.03368 | 1.288 | 0.1978 | 0.7582 | 0.08338 | 0.01154 |
| 12 | 12:121619528:C:T | 121619528 | T | DOMxCHS | -0.1103 | 0.08614 | -0.2792 | 0.0585 | -1.281 | 0.2004 | 1 | 0.00166 | 0.007143 |
| 12 | rs1169737:121600294:C:T | 121600294 | T | DOMxCHS | 0.02307 | 0.01817 | -0.01254 | 0.05867 | 1.27 | 0.2043 | 1 | 0.01983 | 0.002747 |
| 12 | rs1614313:121600692:G:A | 121600692 | A | DOMxCHS | 0.02308 | 0.01818 | -0.01254 | 0.0587 | 1.27 | 0.2043 | 1 | 0.01986 | 0.003846 |
| 12 | rs1169714:121577612:A:G | 121577612 | G | DOMxCHS | 0.0134 | 0.01058 | -0.00734 | 0.03414 | 1.266 | 0.2055 | 0.8755 | 0.08213 | 0.01319 |
| 12 | rs117304865:121608109:C:G | 121608109 | G | DOMxCHS | -0.1002 | 0.08059 | -0.2582 | 0.05773 | -1.244 | 0.2138 | 1 | 0.00142 | 0.03242 |
| 12 | rs139429176:121632160:C:T | 121632160 | T | DOMxCHS | 0.0504 | 0.04102 | -0.02999 | 0.1308 | 1.229 | 0.2193 | 1 | 0.003339 | 0.01264 |
| 12 | 12:121581273:C:T | 121581273 | T | DOMxCHS | 0.06309 | 0.05187 | -0.03858 | 0.1648 | 1.216 | 0.2241 | 1 | 0.003863 | 0.004396 |
| 12 | rs61953400:121614284:G:A | 121614284 | A | DOMxCHS | 0.02059 | 0.01715 | -0.01302 | 0.0542 | 1.201 | 0.2301 | 0.5823 | 0.0219 | 0.008791 |
| 12 | rs11065450 | 121579657 | A | DOMxCHS | -0.00963 | 0.008128 | -0.02556 | 0.006299 | -1.185 | 0.2362 | 1 | 0.1907 | 0 |
| 12 | rs147998595:121578992:C:G | 121578992 | G | DOMxCHS | 0.07022 | 0.05931 | -0.04603 | 0.1865 | 1.184 | 0.2366 | 1 | 0.002206 | 0.003846 |
| 12 | exm1044191 | 121593936 | A | DOMxCHS | -0.05581 | 0.04806 | -0.15 | 0.03839 | -1.161 | 0.2457 | 1 | 0.003846 | 0 |
| 12 | rs208288:121588088:G:C | 121588088 | C | DOMxCHS | 0.01193 | 0.01049 | -0.00863 | 0.03249 | 1.138 | 0.2555 | 1 | 0.08209 | 0.002747 |
| 12 | rs35646440:121580985:G:A | 121580985 | A | DOMxCHS | 0.01146 | 0.01042 | -0.00897 | 0.03188 | 1.099 | 0.2717 | 1 | 0.08329 | 0.01374 |
| 12 | 12:121630071:A:G | 121630071 | G | DOMxCHS | -0.05459 | 0.04966 | -0.1519 | 0.04274 | -1.099 | 0.2718 | 1 | 0.002782 | 0.01264 |
| 12 | rs182839546:121583752:G:A | 121583752 | A | DOMxCHS | 0.138 | 0.1256 | -0.1081 | 0.3841 | 1.099 | 0.2721 | 1 | 0.000836 | 0.01374 |
| 12 | rs608425:121580753:G:A | 121580753 | A | DOMxCHS | 0.01166 | 0.01063 | -0.00918 | 0.03249 | 1.096 | 0.273 | 1 | 0.0825 | 0.01099 |
| 12 | rs1718125:121593019:C:T | 121593019 | T | DOMxCHS | 0.009982 | 0.00913 | -0.00791 | 0.02788 | 1.093 | 0.2744 | 0.6643 | 0.1244 | 0.01264 |
| 12 | rs523977:121569125:C:T | 121569125 | C | DOMxCHS | -0.00892 | 0.008223 | -0.02504 | 0.007193 | -1.085 | 0.278 | 0.6431 | 0.1877 | 0.01209 |
| 12 | rs594423:121579955:G:A | 121579955 | A | DOMxCHS | 0.01129 | 0.01046 | -0.00922 | 0.0318 | 1.079 | 0.2807 | 1 | 0.08273 | 0.01044 |
| 12 | rs607094:121580464:T:C | 121580464 | C | DOMxCHS | 0.01129 | 0.01046 | -0.00922 | 0.0318 | 1.079 | 0.2807 | 1 | 0.08273 | 0.01044 |
| 12 | rs487417:121580541:C:T | 121580541 | T | DOMxCHS | 0.01129 | 0.01046 | -0.00922 | 0.0318 | 1.079 | 0.2807 | 1 | 0.08273 | 0.01044 |
| 12 | rs28360455:121605263:T:C | 121605263 | C | DOMxCHS | -0.05288 | 0.0491 | -0.1491 | 0.04336 | -1.077 | 0.2817 | 1 | 0.001926 | 0.001648 |
| 12 | 12:121598584:C:A | 121598584 | A | DOMxCHS | 0.04803 | 0.04461 | -0.03941 | 0.1355 | 1.077 | 0.2818 | 1 | 0.004135 | 0.003297 |
| 12 | rs622937:121581738:T:C | 121581738 | C | DOMxCHS | 0.01137 | 0.01057 | -0.00935 | 0.0321 | 1.075 | 0.2823 | 1 | 0.08333 | 0.01099 |
| 12 | rs11065443:121564091:G:T | 121564091 | T | DOMxCHS | 0.01624 | 0.01513 | -0.01342 | 0.0459 | 1.073 | 0.2834 | 0.5563 | 0.04242 | 0.02198 |
| 12 | rs76842630:121579502:C:T | 121579502 | T | DOMxCHS | -0.05022 | 0.04689 | -0.1421 | 0.04168 | -1.071 | 0.2843 | 1 | 0.003035 | 0.004396 |
| 12 | rs144847890:121575830:G:A | 121575830 | A | DOMxCHS | -0.04706 | 0.04401 | -0.1333 | 0.0392 | -1.069 | 0.2851 | 1 | 0.005284 | 0.01209 |
| 12 | rs590477:121579078:A:G | 121579078 | G | DOMxCHS | 0.01102 | 0.01041 | -0.00939 | 0.03142 | 1.058 | 0.29 | 1 | 0.08356 | 0.01044 |
| 12 | rs592340:121579516:G:C | 121579516 | C | DOMxCHS | 0.01102 | 0.01041 | -0.00939 | 0.03142 | 1.058 | 0.29 | 1 | 0.08356 | 0.01044 |
| 12 | rs568531:121579673:C:T | 121579673 | T | DOMxCHS | 0.01102 | 0.01041 | -0.00939 | 0.03142 | 1.058 | 0.29 | 1 | 0.08356 | 0.01044 |
| 12 | rs593223:121579721:A:G | 121579721 | G | DOMxCHS | 0.01102 | 0.01041 | -0.00939 | 0.03142 | 1.058 | 0.29 | 1 | 0.08356 | 0.01044 |
| 12 | rs535557:121578387:T:C | 121578387 | C | DOMxCHS | 0.01101 | 0.01041 | -0.0094 | 0.03142 | 1.057 | 0.2905 | 1 | 0.08361 | 0.01099 |
| 12 | rs1794886:121578565:A:C | 121578565 | C | DOMxCHS | 0.01101 | 0.01041 | -0.0094 | 0.03142 | 1.057 | 0.2905 | 1 | 0.08361 | 0.01099 |
| 12 | rs1718148:121578722:C:A | 121578722 | A | DOMxCHS | 0.01101 | 0.01041 | -0.0094 | 0.03142 | 1.057 | 0.2905 | 1 | 0.08361 | 0.01099 |
| 12 | rs1718149:121578725:C:A | 121578725 | A | DOMxCHS | 0.01101 | 0.01041 | -0.0094 | 0.03142 | 1.057 | 0.2905 | 1 | 0.08361 | 0.01099 |
| 12 | rs28360453:121604122:C:T | 121604122 | T | DOMxCHS | -0.01454 | 0.01383 | -0.04165 | 0.01257 | -1.051 | 0.2934 | 0.05793 | 0.04769 | 0.02637 |
| 12 | rs28360448:121600252:G:A | 121600252 | A | DOMxCHS | 0.06703 | 0.06418 | -0.05875 | 0.1928 | 1.044 | 0.2964 | 1 | 0.00139 | 0.01209 |
| 12 | rs510660:121577892:C:T | 121577892 | T | DOMxCHS | 0.01081 | 0.01041 | -0.00959 | 0.03122 | 1.039 | 0.299 | 1 | 0.08389 | 0.01099 |
| 12 | rs511496:121577987:T:C | 121577987 | C | DOMxCHS | 0.01081 | 0.01041 | -0.00959 | 0.03122 | 1.039 | 0.299 | 1 | 0.08389 | 0.01099 |
| 12 | rs1718134:121607891:G:A | 121607891 | G | DOMxCHS | 0.009076 | 0.00887 | -0.00831 | 0.02646 | 1.023 | 0.3064 | 0.3023 | 0.4959 | 0 |
| 12 | 12:121571828:A:T | 121571828 | T | DOMxCHS | 0.05647 | 0.05752 | -0.05626 | 0.1692 | 0.9819 | 0.3263 | 1 | 0.000824 | 0 |
| 12 | rs9737637:121633437:A:G | 121633437 | G | DOMxCHS | 0.008716 | 0.008899 | -0.00873 | 0.02616 | 0.9795 | 0.3275 | 0.3516 | 0.1317 | 0.01978 |
| 12 | 12:121600720:A:G | 121600720 | G | DOMxCHS | 0.05599 | 0.05762 | -0.05695 | 0.1689 | 0.9716 | 0.3314 | 1 | 0.000832 | 0.008791 |
| 12 | rs149089381:121588925:G:A | 121588925 | A | DOMxCHS | -0.04726 | 0.04875 | -0.1428 | 0.04828 | -0.9695 | 0.3324 | 1 | 0.002206 | 0.003846 |
| 12 | rs684201:121570343:A:G | 121570343 | A | DOMxCHS | 0.01474 | 0.01521 | -0.01507 | 0.04454 | 0.9692 | 0.3326 | 0.5367 | 0.04063 | 0.006044 |
| 12 | rs7310821:121567185:C:T | 121567185 | T | DOMxCHS | 0.0146 | 0.01522 | -0.01524 | 0.04443 | 0.9589 | 0.3377 | 0.2407 | 0.04194 | 0.01099 |
| 12 | rs141259616:121601764:C:T | 121601764 | T | DOMxCHS | -0.627 | 0.6629 | -1.926 | 0.6723 | -0.9459 | 0.3444 | 1 | 0.000827 | 0.003846 |
| 12 | rs75497300:121626365:G:C | 121626365 | C | DOMxCHS | 0.03039 | 0.03224 | -0.03279 | 0.09358 | 0.9428 | 0.3459 | 1 | 0.006254 | 0.03352 |
| 12 | 12:121588341:G:A | 121588341 | A | DOMxCHS | 0.05414 | 0.05756 | -0.05867 | 0.167 | 0.9407 | 0.347 | 1 | 0.00222 | 0.00989 |
| 12 | rs503720:121605074:G:A | 121605074 | A | DOMxCHS | -0.00808 | 0.008613 | -0.02496 | 0.008803 | -0.9378 | 0.3485 | 0.8496 | 0.2825 | 0.1637 |
| 12 | rs11065442:121563896:A:C | 121563896 | C | DOMxCHS | -0.00799 | 0.00854 | -0.02473 | 0.008748 | -0.9356 | 0.3496 | 0.1347 | 0.1601 | 0.02033 |
| 12 | rs146784302:121588256:T:C | 121588256 | C | DOMxCHS | -0.02465 | 0.02643 | -0.07646 | 0.02716 | -0.9325 | 0.3512 | 0.3018 | 0.01417 | 0.01099 |
| 12 | rs187319818:121624580:G:A | 121624580 | A | DOMxCHS | -0.01648 | 0.01787 | -0.0515 | 0.01854 | -0.9223 | 0.3565 | 0.08609 | 0.01678 | 0.01758 |
| 12 | psy_rs61953403 | 121617990 | G | DOMxCHS | -0.00899 | 0.009824 | -0.02825 | 0.01026 | -0.9152 | 0.3602 | 0.7925 | 0.09918 | 0 |

| 12 | rs201007044:121565433:C:T | 121565433 | T | DOMxCHS | 0.01079 | 0.0118 | -0.01234 | 0.03391 | 0.9143 | 0.3607 | 0.3765 | 0.1042 | 0.2802 |
| --- | --- | --- | --- | --- | --- | --- | --- | --- | --- | --- | --- | --- | --- |
| 12 | rs11065445:121568945:C:G | 121568945 | G | DOMxCHS | 0.01379 | 0.0152 | -0.01601 | 0.04359 | 0.907 | 0.3645 | 0.5333 | 0.04031 | 0.004945 |
| 12 | rs3900976:121571221:C:T | 121571221 | T | DOMxCHS | 0.01379 | 0.0152 | -0.01601 | 0.04359 | 0.907 | 0.3645 | 0.5333 | 0.04031 | 0.004945 |
| 12 | rs2393800:121571779:G:A | 121571779 | A | DOMxCHS | 0.01379 | 0.0152 | -0.01601 | 0.04359 | 0.907 | 0.3645 | 0.5333 | 0.04031 | 0.004945 |
| 12 | 12:121630627:G:C | 121630627 | C | DOMxCHS | 0.139 | 0.1534 | -0.1616 | 0.4396 | 0.9063 | 0.3649 | 1 | 0.000552 | 0.004945 |
| 12 | rs61953396:121600738:A:G | 121600738 | G | DOMxCHS | 0.014 | 0.01547 | -0.01633 | 0.04433 | 0.9049 | 0.3656 | 0.7502 | 0.03924 | 0.02692 |
| 12 | rs11065458:121598244:C:T | 121598244 | T | DOMxCHS | 0.01396 | 0.01545 | -0.01632 | 0.04423 | 0.9034 | 0.3665 | 0.754 | 0.03943 | 0.01758 |
| 12 | rs3891262:121563481:C:T | 121563481 | T | DOMxCHS | 0.01372 | 0.01521 | -0.01609 | 0.04354 | 0.902 | 0.3672 | 0.5405 | 0.04097 | 0.007692 |
| 12 | rs11065441:121560971:T:C | 121560971 | C | DOMxCHS | 0.01359 | 0.01519 | -0.01618 | 0.04336 | 0.8946 | 0.3711 | 0.5394 | 0.04079 | 0.003297 |
| 12 | rs7313004:121562854:C:T | 121562854 | T | DOMxCHS | 0.01359 | 0.01519 | -0.01618 | 0.04336 | 0.8946 | 0.3711 | 0.5394 | 0.04079 | 0.003297 |
| 12 | rs3861798:121562036:T:C | 121562036 | C | DOMxCHS | 0.01359 | 0.0152 | -0.0162 | 0.04338 | 0.8941 | 0.3714 | 0.5396 | 0.04084 | 0.004396 |
| 12 | rs181033264:121631506:C:G | 121631506 | G | DOMxCHS | 0.1252 | 0.1408 | -0.1508 | 0.4012 | 0.8889 | 0.3742 | 1 | 0.0011 | 0.001099 |
| 12 | 12:121632301:G:A | 121632301 | A | DOMxCHS | 0.1252 | 0.1408 | -0.1508 | 0.4012 | 0.8889 | 0.3742 | 1 | 0.0011 | 0.001099 |
| 12 | rs117556439:121591748:C:G | 121591748 | G | DOMxCHS | 0.5854 | 0.6587 | -0.7056 | 1.876 | 0.8888 | 0.3743 | 1 | 0.000832 | 0.00989 |
| 12 | rs208278:121560664:G:A | 121560664 | G | DOMxCHS | -0.00697 | 0.008039 | -0.02273 | 0.008785 | -0.8672 | 0.3859 | 0.0201 | 0.2056 | 0.02033 |
| 12 | rs208279:121560724:T:A | 121560724 | T | DOMxCHS | -0.00697 | 0.008039 | -0.02273 | 0.008785 | -0.8672 | 0.3859 | 0.0201 | 0.2056 | 0.02033 |
| 12 | rs208282:121562104:G:A | 121562104 | G | DOMxCHS | -0.00697 | 0.008039 | -0.02273 | 0.008785 | -0.8672 | 0.3859 | 0.0201 | 0.2056 | 0.02033 |
| 12 | rs208283:121562135:G:A | 121562135 | G | DOMxCHS | -0.00697 | 0.008039 | -0.02273 | 0.008785 | -0.8672 | 0.3859 | 0.0201 | 0.2056 | 0.02033 |
| 12 | rs1718124 | 121594460 | C | DOMxCHS | 0.0089 | 0.01032 | -0.01133 | 0.02913 | 0.8624 | 0.3886 | 0.8772 | 0.08297 | 0 |
| 12 | rs12304035 | 121620145 | A | DOMxCHS | -0.00879 | 0.01025 | -0.02888 | 0.01131 | -0.8569 | 0.3916 | 0.3922 | 0.09071 | 0.00055 |
| 12 | rs1653585:121593355:C:A | 121593355 | A | DOMxCHS | 0.008835 | 0.01033 | -0.0114 | 0.02907 | 0.8556 | 0.3923 | 1 | 0.08246 | 0.00055 |
| 12 | rs1653584:121593371:C:T | 121593371 | T | DOMxCHS | 0.008835 | 0.01033 | -0.0114 | 0.02907 | 0.8556 | 0.3923 | 1 | 0.08246 | 0.00055 |
| 12 | rs1718162:121597631:G:A | 121597631 | A | DOMxCHS | 0.008835 | 0.01033 | -0.0114 | 0.02907 | 0.8556 | 0.3923 | 1 | 0.08246 | 0.00055 |
| 12 | rs1794887:121598565:G:A | 121598565 | A | DOMxCHS | 0.008835 | 0.01033 | -0.0114 | 0.02907 | 0.8556 | 0.3923 | 1 | 0.08246 | 0.00055 |
| 12 | rs1653583:121598652:G:A | 121598652 | A | DOMxCHS | 0.008835 | 0.01033 | -0.0114 | 0.02907 | 0.8556 | 0.3923 | 1 | 0.08246 | 0.00055 |
| 12 | rs2686369:121590949:C:T | 121590949 | T | DOMxCHS | 0.008823 | 0.01033 | -0.01142 | 0.02907 | 0.8543 | 0.3931 | 1 | 0.08251 | 0.001099 |
| 12 | rs191954471:121616896:G:T | 121616896 | T | DOMxCHS | -0.06832 | 0.08004 | -0.2252 | 0.08856 | -0.8535 | 0.3935 | 1 | 0.002483 | 0.004396 |
| 12 | rs10849850:121589462:A:G | 121589462 | G | DOMxCHS | 0.01262 | 0.01508 | -0.01693 | 0.04217 | 0.8369 | 0.4028 | 0.5475 | 0.04148 | 0.006593 |
| 12 | rs79362551:121579526:G:A | 121579526 | A | DOMxCHS | 0.01265 | 0.01516 | -0.01706 | 0.04237 | 0.8347 | 0.404 | 0.5333 | 0.04031 | 0.004945 |
| 12 | rs10849849:121586395:A:G | 121586395 | G | DOMxCHS | 0.01256 | 0.01506 | -0.01696 | 0.04208 | 0.8341 | 0.4043 | 0.5469 | 0.04139 | 0.004396 |
| 12 | rs148626102:121624954:T:C | 121624954 | C | DOMxCHS | -0.054 | 0.06484 | -0.1811 | 0.07308 | -0.8328 | 0.4051 | 1 | 0.003083 | 0.01978 |
| 12 | rs192778594:121567929:A:C | 121567929 | C | DOMxCHS | 0.04651 | 0.05593 | -0.06312 | 0.1561 | 0.8315 | 0.4058 | 1 | 0.001654 | 0.003297 |
| 12 | rs11065452:121581982:G:T | 121581982 | T | DOMxCHS | 0.0125 | 0.0151 | -0.01709 | 0.0421 | 0.8279 | 0.4078 | 0.5432 | 0.04111 | 0.004396 |
| 12 | rs11065453:121581983:C:T | 121581983 | T | DOMxCHS | 0.0125 | 0.0151 | -0.01709 | 0.0421 | 0.8279 | 0.4078 | 0.5432 | 0.04111 | 0.004396 |
| 12 | rs10849848:121582048:T:C | 121582048 | C | DOMxCHS | 0.0125 | 0.0151 | -0.01709 | 0.0421 | 0.8279 | 0.4078 | 0.5432 | 0.04111 | 0.004396 |
| 12 | rs187011593:121597819:C:T | 121597819 | T | DOMxCHS | -0.09222 | 0.112 | -0.3118 | 0.1273 | -0.8232 | 0.4105 | 1 | 0.001953 | 0.01538 |
| 12 | rs146878525:121597696:A:T | 121597696 | T | DOMxCHS | 0.04576 | 0.05592 | -0.06384 | 0.1554 | 0.8183 | 0.4133 | 1 | 0.001657 | 0.004945 |
| 12 | 12:121617258:G:A | 121617258 | A | DOMxCHS | 0.09429 | 0.1161 | -0.1332 | 0.3218 | 0.8123 | 0.4168 | 1 | 0.001119 | 0.01813 |
| 12 | rs117323590:121581618:G:A | 121581618 | A | DOMxCHS | 0.09745 | 0.1218 | -0.1412 | 0.3361 | 0.8003 | 0.4236 | 1 | 0.002256 | 0.02582 |
| 12 | rs113787960:121601299:A:G | 121601299 | G | DOMxCHS | -0.02083 | 0.02627 | -0.07231 | 0.03065 | -0.7931 | 0.4278 | 1 | 0.01263 | 0.02143 |
| 12 | rs10849851:121596644:A:G | 121596644 | G | DOMxCHS | 0.01199 | 0.01514 | -0.01769 | 0.04167 | 0.792 | 0.4285 | 0.5459 | 0.04153 | 0.01429 |
| 12 | rs374936671:121626310:C:T | 121626310 | T | DOMxCHS | 0.07053 | 0.08913 | -0.1042 | 0.2452 | 0.7914 | 0.4288 | 1 | 0.002205 | 0.003297 |
| 12 | 12:121574865:G:A | 121574865 | A | DOMxCHS | -0.04267 | 0.05477 | -0.15 | 0.06467 | -0.7792 | 0.436 | 1 | 0.000551 | 0.002747 |
| 12 | 12:121590250:G:A | 121590250 | A | DOMxCHS | -0.04267 | 0.05477 | -0.15 | 0.06467 | -0.7792 | 0.436 | 1 | 0.000551 | 0.002747 |
| 12 | rs75794459:121587037:T:A | 121587037 | A | DOMxCHS | 0.196 | 0.255 | -0.3038 | 0.6958 | 0.7685 | 0.4423 | 1 | 0.00055 | 0.00055 |
| 12 | rs148512694:121609750:C:T | 121609750 | T | DOMxCHS | 0.03769 | 0.04928 | -0.0589 | 0.1343 | 0.7648 | 0.4445 | 1 | 0.003882 | 0.009341 |
| 12 | rs208292:121596191:A:G | 121596191 | G | DOMxCHS | 0.0115 | 0.0151 | -0.01811 | 0.0411 | 0.7613 | 0.4466 | 0.5504 | 0.0419 | 0.01648 |
| 12 | rs117048777:121573621:C:T | 121573621 | T | DOMxCHS | 0.009639 | 0.01267 | -0.0152 | 0.03448 | 0.7606 | 0.447 | 1 | 0.05418 | 0.02143 |
| 12 | rs475836:121604146:C:G | 121604146 | G | DOMxCHS | 0.007015 | 0.009249 | -0.01111 | 0.02514 | 0.7584 | 0.4483 | 0.8841 | 0.4938 | 0.06978 |
| 12 | rs12301635:121624108:C:G | 121624108 | G | DOMxCHS | -0.00777 | 0.01028 | -0.02792 | 0.01238 | -0.7559 | 0.4498 | 0.3892 | 0.09026 | 0.001648 |
| 12 | rs34405253:121617378:C:G | 121617378 | G | DOMxCHS | -0.00764 | 0.01027 | -0.02778 | 0.01249 | -0.744 | 0.4569 | 0.3903 | 0.09038 | 0 |
| 12 | rs73421551:121561221:C:T | 121561221 | T | DOMxCHS | 0.009158 | 0.01252 | -0.01538 | 0.03369 | 0.7315 | 0.4645 | 1 | 0.05736 | 0.01813 |
| 12 | rs73421553:121561921:C:T | 121561921 | T | DOMxCHS | 0.009158 | 0.01252 | -0.01538 | 0.03369 | 0.7315 | 0.4645 | 1 | 0.05736 | 0.01813 |
| 12 | rs2686371:121604724:A:G | 121604724 | G | DOMxCHS | -0.00799 | 0.01096 | -0.02947 | 0.01349 | -0.729 | 0.4661 | 0.6322 | 0.08121 | 0.02912 |
| 12 | 12:121580559:G:A | 121580559 | A | DOMxCHS | -0.1854 | 0.2545 | -0.6841 | 0.3133 | -0.7285 | 0.4664 | 1 | 0.000832 | 0.00989 |
| 12 | rs79037236:121590747:G:A | 121590747 | A | DOMxCHS | 0.007526 | 0.01039 | -0.01283 | 0.02788 | 0.7246 | 0.4688 | 1 | 0.08132 | 0.02363 |
| 12 | rs57263663:121566204:C:T | 121566204 | T | DOMxCHS | 0.008948 | 0.01252 | -0.01559 | 0.03348 | 0.7149 | 0.4748 | 1 | 0.05736 | 0.01813 |
| 12 | rs111673922:121566423:G:A | 121566423 | A | DOMxCHS | 0.008948 | 0.01252 | -0.01559 | 0.03348 | 0.7149 | 0.4748 | 1 | 0.05736 | 0.01813 |
| 12 | rs76713875:121613144:G:C | 121613144 | C | DOMxCHS | -0.07184 | 0.1015 | -0.2707 | 0.127 | -0.7082 | 0.4789 | 1 | 0.000826 | 0.002747 |
| 12 | rs147014194:121628089:G:A | 121628089 | A | DOMxCHS | -0.04164 | 0.06213 | -0.1634 | 0.08014 | -0.6702 | 0.5028 | 1 | 0.004741 | 0.01484 |
| 12 | 12:121632994:G:A | 121632994 | A | DOMxCHS | -0.02645 | 0.04016 | -0.1052 | 0.05225 | -0.6588 | 0.5101 | 1 | 0.0022 | 0.001099 |
| 12 | rs11065476:121631103:T:G | 121631103 | G | DOMxCHS | -0.1635 | 0.2505 | -0.6545 | 0.3275 | -0.6527 | 0.5141 | 1 | 0.000825 | 0.00055 |
| 12 | rs2857587:121602515:G:A | 121602515 | A | DOMxCHS | -0.00694 | 0.01083 | -0.02816 | 0.01428 | -0.6407 | 0.5218 | 1 | 0.08329 | 0.02363 |
| 12 | rs2051551:121602628:T:C | 121602628 | C | DOMxCHS | -0.00694 | 0.01083 | -0.02816 | 0.01428 | -0.6407 | 0.5218 | 1 | 0.08329 | 0.02363 |
| 12 | rs2051550:121602469:C:T | 121602469 | T | DOMxCHS | -0.00688 | 0.01084 | -0.02813 | 0.01437 | -0.6348 | 0.5256 | 1 | 0.08347 | 0.02582 |
| 12 | 12:121616876:G:T | 121616876 | T | DOMxCHS | 0.04845 | 0.07648 | -0.1015 | 0.1984 | 0.6335 | 0.5265 | 1 | 0.000552 | 0.003846 |
| 12 | 12:121626343:G:A | 121626343 | A | DOMxCHS | 0.04845 | 0.07648 | -0.1015 | 0.1984 | 0.6335 | 0.5265 | 1 | 0.000552 | 0.003846 |
| 12 | rs142424712:121627805:G:A | 121627805 | A | DOMxCHS | 0.04845 | 0.07648 | -0.1015 | 0.1984 | 0.6335 | 0.5265 | 1 | 0.000552 | 0.003846 |
| 12 | 12:121634349:G:C | 121634349 | C | DOMxCHS | 0.04845 | 0.07648 | -0.1015 | 0.1984 | 0.6335 | 0.5265 | 1 | 0.000552 | 0.003846 |
| 12 | rs208291:121594170:G:A | 121594170 | A | DOMxCHS | 0.006369 | 0.01022 | -0.01366 | 0.02639 | 0.6234 | 0.5331 | 0.8838 | 0.08851 | 0.00989 |
| 12 | rs12826364:121614324:G:A | 121614324 | A | DOMxCHS | 0.004899 | 0.00793 | -0.01064 | 0.02044 | 0.6177 | 0.5368 | 0.09172 | 0.2701 | 0.02473 |
| 12 | rs1653613:121607647:G:T | 121607647 | G | DOMxCHS | 0.01113 | 0.01806 | -0.02427 | 0.04654 | 0.6163 | 0.5378 | 0.57 | 0.02145 | 0.001099 |
| 12 | rs1653624:121622520:A:T | 121622520 | A | DOMxCHS | 0.01113 | 0.01806 | -0.02427 | 0.04653 | 0.6161 | 0.5379 | 0.57 | 0.02145 | 0.001099 |
| 12 | rs142399888:121631995:C:T | 121631995 | T | DOMxCHS | 0.02115 | 0.03509 | -0.04762 | 0.08992 | 0.6028 | 0.5467 | 1 | 0.009637 | 0.002198 |
| 12 | 12:121624411:G:A | 121624411 | A | DOMxCHS | 0.02452 | 0.04069 | -0.05524 | 0.1043 | 0.6026 | 0.5469 | 1 | 0.004712 | 0.008791 |
| 12 | rs118049242:121628288:C:T | 121628288 | T | DOMxCHS | 0.01238 | 0.02067 | -0.02814 | 0.0529 | 0.5987 | 0.5495 | 0.02924 | 0.0124 | 0.003297 |
| 12 | rs17526121:121603808:A:T | 121603808 | T | DOMxCHS | 0.006319 | 0.01057 | -0.01441 | 0.02704 | 0.5976 | 0.5502 | 0.3347 | 0.08015 | 0.02308 |
| 12 | rs150489232:121589715:T:G | 121589715 | G | DOMxCHS | 0.015 | 0.02519 | -0.03438 | 0.06438 | 0.5955 | 0.5516 | 0.3714 | 0.01603 | 0.006044 |
| 12 | rs138309674:121589720:G:T | 121589720 | T | DOMxCHS | 0.015 | 0.02519 | -0.03438 | 0.06438 | 0.5955 | 0.5516 | 0.3714 | 0.01603 | 0.006044 |
| 12 | exm1044214 | 121603240 | A | DOMxCHS | -0.2268 | 0.3824 | -0.9764 | 0.5228 | -0.593 | 0.5532 | 1 | 0.001099 | 0 |
| 12 | rs55814439:121596786:C:T | 121596786 | T | DOMxCHS | 0.005865 | 0.01022 | -0.01416 | 0.02589 | 0.574 | 0.566 | 0.8838 | 0.08828 | 0.01044 |
| 12 | rs1182946:121601893:A:C | 121601893 | C | DOMxCHS | 0.01034 | 0.01814 | -0.02522 | 0.04589 | 0.5698 | 0.5689 | 0.5523 | 0.02098 | 0.004945 |
| 12 | rs2857589:121604476:C:A | 121604476 | A | DOMxCHS | -0.00625 | 0.01098 | -0.02777 | 0.01526 | -0.5698 | 0.5689 | 1 | 0.08159 | 0.02692 |
| 12 | rs2857588:121604325:C:T | 121604325 | T | DOMxCHS | -0.00624 | 0.01095 | -0.0277 | 0.01523 | -0.5693 | 0.5693 | 1 | 0.08268 | 0.02308 |
| 12 | 12:121630763:A:G | 121630763 | G | DOMxCHS | -0.02069 | 0.03692 | -0.09304 | 0.05167 | -0.5604 | 0.5753 | 1 | 0.005134 | 0.03681 |
| 12 | 12:121630764:T:G | 121630764 | G | DOMxCHS | -0.02069 | 0.03692 | -0.09304 | 0.05167 | -0.5604 | 0.5753 | 1 | 0.005134 | 0.03681 |

| 12 | 12:121630783:T:A | 121630783 | A | DOMxCHS | -0.02069 | 0.03692 | -0.09304 | 0.05167 | -0.5604 | 0.5753 | 1 | 0.005134 | 0.03681 |
| --- | --- | --- | --- | --- | --- | --- | --- | --- | --- | --- | --- | --- | --- |
| 12 | rs7137837:121613046:C:T | 121613046 | T | DOMxCHS | -0.0046 | 0.008239 | -0.02075 | 0.01155 | -0.5579 | 0.577 | 0.1509 | 0.1795 | 0.006593 |
| 12 | rs111337093:121602635:C:T | 121602635 | T | DOMxCHS | -0.01118 | 0.02014 | -0.05065 | 0.02828 | -0.5554 | 0.5787 | 0.5745 | 0.02171 | 0.01319 |
| 12 | rs76481706:121603294:T:C | 121603294 | C | DOMxCHS | -0.01118 | 0.02014 | -0.05065 | 0.02828 | -0.5554 | 0.5787 | 0.5745 | 0.02171 | 0.01319 |
| 12 | rs183248117:121628092:G:T | 121628092 | T | DOMxCHS | -0.2109 | 0.3827 | -0.961 | 0.5391 | -0.5512 | 0.5815 | 1 | 0.000551 | 0.002198 |
| 12 | rs181391234:121595797:G:A | 121595797 | A | DOMxCHS | 0.02656 | 0.04852 | -0.06853 | 0.1217 | 0.5475 | 0.5841 | 1 | 0.004128 | 0.001648 |
| 12 | rs183374345:121571804:G:A | 121571804 | A | DOMxCHS | 0.04679 | 0.08574 | -0.1212 | 0.2148 | 0.5458 | 0.5853 | 1 | 0.00275 | 0.001099 |
| 12 | rs76190743:121604252:A:G | 121604252 | G | DOMxCHS | -0.01099 | 0.02015 | -0.05047 | 0.0285 | -0.5454 | 0.5855 | 0.5743 | 0.0217 | 0.01264 |
| 12 | 12:121607890:C:T | 121607890 | T | DOMxCHS | 0.07883 | 0.1445 | -0.2044 | 0.3621 | 0.5455 | 0.5855 | 1 | 0.0011 | 0.00055 |
| 12 | 12:121583492:G:A | 121583492 | A | DOMxCHS | 0.01776 | 0.03312 | -0.04715 | 0.08268 | 0.5364 | 0.5917 | 1 | 0.006623 | 0.004396 |
| 12 | 12:121598011:C:T | 121598011 | T | DOMxCHS | -0.2042 | 0.3814 | -0.9517 | 0.5433 | -0.5354 | 0.5924 | 1 | 0.000552 | 0.004945 |
| 12 | rs78906567:121594894:G:A | 121594894 | A | DOMxCHS | 0.006485 | 0.01221 | -0.01744 | 0.03041 | 0.5313 | 0.5953 | 1 | 0.05648 | 0.007692 |
| 12 | rs74729617:121590811:T:C | 121590811 | C | DOMxCHS | 0.006483 | 0.01221 | -0.01744 | 0.03041 | 0.5311 | 0.5954 | 1 | 0.05651 | 0.008242 |
| 12 | rs11065464:121602135:C:A | 121602135 | A | DOMxCHS | 0.004334 | 0.008158 | -0.01166 | 0.02032 | 0.5312 | 0.5954 | 0.4585 | 0.2388 | 0.08407 |
| 12 | rs28360446:121600140:G:A | 121600140 | A | DOMxCHS | -0.01043 | 0.01971 | -0.04906 | 0.02821 | -0.5289 | 0.5969 | 0.5552 | 0.02115 | 0.01264 |
| 12 | rs73218213:121560835:G:A | 121560835 | A | DOMxCHS | -0.01894 | 0.03584 | -0.08919 | 0.0513 | -0.5286 | 0.5972 | 1 | 0.005966 | 0.03297 |
| 12 | rs71454682:121612098:G:C | 121612098 | C | DOMxCHS | -0.0051 | 0.009718 | -0.02414 | 0.01395 | -0.5246 | 0.5999 | 0.7415 | 0.1236 | 0.03791 |
| 12 | rs192047692:121560733:A:C | 121560733 | C | DOMxCHS | -0.1337 | 0.2552 | -0.6339 | 0.3664 | -0.5241 | 0.6003 | 1 | 0.00055 | 0 |
| 12 | 12:121563985:C:T | 121563985 | T | DOMxCHS | -0.1337 | 0.2552 | -0.6339 | 0.3664 | -0.5241 | 0.6003 | 1 | 0.00055 | 0 |
| 12 | rs145202233:121594435:T:C | 121594435 | C | DOMxCHS | -0.1337 | 0.2552 | -0.6339 | 0.3664 | -0.5241 | 0.6003 | 1 | 0.00055 | 0 |
| 12 | rs147694021:121605514:C:T | 121605514 | T | DOMxCHS | -0.1337 | 0.2552 | -0.6339 | 0.3664 | -0.5241 | 0.6003 | 1 | 0.00055 | 0 |
| 12 | rs151243516:121606181:G:A | 121606181 | A | DOMxCHS | -0.1337 | 0.2552 | -0.6339 | 0.3664 | -0.5241 | 0.6003 | 1 | 0.00055 | 0 |
| 12 | rs143390454:121607662:C:G | 121607662 | G | DOMxCHS | -0.1337 | 0.2552 | -0.6339 | 0.3664 | -0.5241 | 0.6003 | 1 | 0.00055 | 0 |
| 12 | rs372101643:121609880:G:A | 121609880 | A | DOMxCHS | -0.1337 | 0.2552 | -0.6339 | 0.3664 | -0.5241 | 0.6003 | 1 | 0.00055 | 0 |
| 12 | 12:121614567:A:C | 121614567 | C | DOMxCHS | -0.1337 | 0.2552 | -0.6339 | 0.3664 | -0.5241 | 0.6003 | 1 | 0.00055 | 0 |
| 12 | rs147788233:121616819:T:C | 121616819 | C | DOMxCHS | -0.1337 | 0.2552 | -0.6339 | 0.3664 | -0.5241 | 0.6003 | 1 | 0.00055 | 0 |
| 12 | rs12821688:121608585:A:T | 121608585 | T | DOMxCHS | -0.00538 | 0.0104 | -0.02575 | 0.015 | -0.5175 | 0.6049 | 0.389 | 0.09016 | 0.00055 |
| 12 | rs12321707:121609494:C:T | 121609494 | T | DOMxCHS | -0.00538 | 0.0104 | -0.02575 | 0.015 | -0.5175 | 0.6049 | 0.389 | 0.09016 | 0.00055 |
| 12 | 12:121633995:A:T | 121633995 | T | DOMxCHS | -0.1316 | 0.2543 | -0.63 | 0.3669 | -0.5174 | 0.605 | 1 | 0.000552 | 0.004945 |
| 12 | rs112803307:121605508:A:G | 121605508 | G | DOMxCHS | 0.008012 | 0.01555 | -0.02247 | 0.0385 | 0.5151 | 0.6066 | 0.4659 | 0.03453 | 0.02143 |
| 12 | rs560422:121575924:G:A | 121575924 | G | DOMxCHS | 0.005197 | 0.01016 | -0.01472 | 0.02511 | 0.5115 | 0.6091 | 0.8838 | 0.08827 | 0.007143 |
| 12 | rs12819741:121613603:A:T | 121613603 | T | DOMxCHS | -0.00491 | 0.009697 | -0.02391 | 0.0141 | -0.5059 | 0.613 | 0.7417 | 0.1237 | 0.03846 |
| 12 | 12:121585995:A:T | 121585995 | T | DOMxCHS | 0.03195 | 0.06339 | -0.09229 | 0.1562 | 0.5041 | 0.6143 | 1 | 0.002204 | 0.002747 |
| 12 | rs74356556:121579290:G:C | 121579290 | C | DOMxCHS | 0.005011 | 0.01013 | -0.01484 | 0.02486 | 0.4949 | 0.6208 | 0.884 | 0.08858 | 0.004396 |
| 12 | rs17434682:121579412:T:C | 121579412 | C | DOMxCHS | 0.005011 | 0.01013 | -0.01484 | 0.02486 | 0.4949 | 0.6208 | 0.884 | 0.08858 | 0.004396 |
| 12 | rs56017922:121583828:G:A | 121583828 | A | DOMxCHS | 0.005011 | 0.01013 | -0.01484 | 0.02486 | 0.4949 | 0.6208 | 0.884 | 0.08858 | 0.004396 |
| 12 | rs17434731:121583993:C:T | 121583993 | T | DOMxCHS | 0.005011 | 0.01013 | -0.01484 | 0.02486 | 0.4949 | 0.6208 | 0.884 | 0.08858 | 0.004396 |
| 12 | rs7973360:121584681:C:T | 121584681 | T | DOMxCHS | 0.005011 | 0.01013 | -0.01484 | 0.02486 | 0.4949 | 0.6208 | 0.884 | 0.08858 | 0.004396 |
| 12 | rs17434809:121585228:A:C | 121585228 | C | DOMxCHS | 0.005011 | 0.01013 | -0.01484 | 0.02486 | 0.4949 | 0.6208 | 0.884 | 0.08858 | 0.004396 |
| 12 | rs17525767:121588125:C:T | 121588125 | T | DOMxCHS | 0.005011 | 0.01013 | -0.01484 | 0.02486 | 0.4949 | 0.6208 | 0.884 | 0.08858 | 0.004396 |
| 12 | 12:121614767:T:C | 121614767 | C | DOMxCHS | 0.0899 | 0.1823 | -0.2674 | 0.4472 | 0.4931 | 0.622 | 1 | 0.001108 | 0.008242 |
| 12 | rs1963773:121601308:A:G | 121601308 | G | DOMxCHS | 0.00517 | 0.01061 | -0.01563 | 0.02597 | 0.4871 | 0.6262 | 0.196 | 0.08034 | 0.03571 |
| 12 | rs113838402:121601748:C:T | 121601748 | T | DOMxCHS | -0.00972 | 0.02005 | -0.04902 | 0.02958 | -0.4847 | 0.6279 | 0.5852 | 0.02207 | 0.01648 |
| 12 | 12:121630771:A:G | 121630771 | G | DOMxCHS | -0.01739 | 0.03608 | -0.08811 | 0.05333 | -0.4819 | 0.6299 | 1 | 0.005705 | 0.03681 |
| 12 | 12:121602065:G:T | 121602065 | T | DOMxCHS | 0.0732 | 0.152 | -0.2248 | 0.3712 | 0.4815 | 0.6302 | 1 | 0.000825 | 0.001099 |
| 12 | 12:121628100:G:A | 121628100 | A | DOMxCHS | 0.02478 | 0.05164 | -0.07644 | 0.126 | 0.4798 | 0.6314 | 1 | 0.003044 | 0.007143 |
| 12 | exm1044260 | 121618257 | C | DOMxCHS | -0.02874 | 0.06111 | -0.1485 | 0.09104 | -0.4703 | 0.6382 | 1 | 0.001374 | 0 |
| 12 | rs117073356:121630706:T:A | 121630706 | A | DOMxCHS | -0.06573 | 0.1426 | -0.3453 | 0.2138 | -0.4609 | 0.645 | 1 | 0.003081 | 0.01923 |
| 12 | rs7959194:121575987:G:A | 121575987 | A | DOMxCHS | 0.004648 | 0.01015 | -0.01524 | 0.02454 | 0.4581 | 0.647 | 0.8838 | 0.08817 | 0.006044 |
| 12 | rs148438900:121620401:T:C | 121620401 | C | DOMxCHS | -0.0366 | 0.07994 | -0.1933 | 0.1201 | -0.4578 | 0.6471 | 1 | 0.001661 | 0.007692 |
| 12 | rs28969483:121623633:C:T | 121623633 | T | DOMxCHS | -0.1739 | 0.3828 | -0.9242 | 0.5763 | -0.4544 | 0.6496 | 1 | 0.00055 | 0.00055 |
| 12 | rs2567990:121610411:A:G | 121610411 | A | DOMxCHS | 0.006255 | 0.01385 | -0.02088 | 0.03339 | 0.4518 | 0.6515 | 0.7664 | 0.04108 | 0.01703 |
| 12 | 12:121630620:T:C | 121630620 | C | DOMxCHS | 0.02267 | 0.05117 | -0.07762 | 0.123 | 0.4431 | 0.6578 | 1 | 0.00166 | 0.007143 |
| 12 | rs73218221:121567885:G:A | 121567885 | A | DOMxCHS | 0.004489 | 0.01018 | -0.01547 | 0.02445 | 0.4407 | 0.6595 | 1 | 0.08713 | 0.00989 |
| 12 | rs28360439:121568201:T:C | 121568201 | C | DOMxCHS | 0.004489 | 0.01018 | -0.01547 | 0.02445 | 0.4407 | 0.6595 | 1 | 0.08713 | 0.00989 |
| 12 | rs28969463:121568371:T:G | 121568371 | G | DOMxCHS | 0.004489 | 0.01018 | -0.01547 | 0.02445 | 0.4407 | 0.6595 | 1 | 0.08713 | 0.00989 |
| 12 | rs17434647:121579024:T:G | 121579024 | G | DOMxCHS | -0.00417 | 0.009678 | -0.02314 | 0.0148 | -0.4312 | 0.6664 | 0.1218 | 0.1035 | 0.03407 |
| 12 | rs147798098:121589199:C:T | 121589199 | T | DOMxCHS | -0.00417 | 0.009682 | -0.02315 | 0.0148 | -0.4311 | 0.6665 | 0.1221 | 0.1036 | 0.03516 |
| 12 | rs186783012:121617172:G:A | 121617172 | A | DOMxCHS | -0.08025 | 0.191 | -0.4546 | 0.2941 | -0.4202 | 0.6744 | 1 | 0.000552 | 0.003846 |
| 12 | rs115486488:121567731:T:G | 121567731 | G | DOMxCHS | 0.00428 | 0.01021 | -0.01574 | 0.0243 | 0.4191 | 0.6752 | 1 | 0.08652 | 0.009341 |
| 12 | 12:121560946:C:A | 121560946 | A | DOMxCHS | -0.04975 | 0.119 | -0.2831 | 0.1836 | -0.418 | 0.676 | 1 | 0.000825 | 0.001099 |
| 12 | 12:121580260:C:T | 121580260 | T | DOMxCHS | -0.04975 | 0.119 | -0.2831 | 0.1836 | -0.418 | 0.676 | 1 | 0.000825 | 0.001099 |
| 12 | rs147402932:121620337:T:C | 121620337 | C | DOMxCHS | -0.02282 | 0.05479 | -0.1302 | 0.08457 | -0.4165 | 0.6771 | 1 | 0.000551 | 0.002747 |
| 12 | rs11065457:121588225:G:A | 121588225 | A | DOMxCHS | -0.07579 | 0.1831 | -0.4348 | 0.2832 | -0.4138 | 0.6791 | 1 | 0.001102 | 0.002747 |
| 12 | rs117878399:121560631:C:T | 121560631 | T | DOMxCHS | 0.02735 | 0.0674 | -0.1047 | 0.1594 | 0.4058 | 0.6849 | 1 | 0.003064 | 0.01374 |
| 12 | rs141707772:121631392:G:A | 121631392 | A | DOMxCHS | -0.01318 | 0.03328 | -0.0784 | 0.05205 | -0.396 | 0.6922 | 1 | 0.007533 | 0.01538 |
| 12 | 12:121621921:C:T | 121621921 | T | DOMxCHS | 0.02926 | 0.07422 | -0.1162 | 0.1747 | 0.3942 | 0.6935 | 1 | 0.001651 | 0.001648 |
| 12 | rs113657985:121564665:G:A | 121564665 | A | DOMxCHS | 0.01434 | 0.03766 | -0.05947 | 0.08815 | 0.3808 | 0.7034 | 1 | 0.008718 | 0.02308 |
| 12 | rs113711923:121564949:C:T | 121564949 | T | DOMxCHS | 0.01434 | 0.03766 | -0.05947 | 0.08815 | 0.3808 | 0.7034 | 1 | 0.008718 | 0.02308 |
| 12 | rs150119521:121585618:G:A | 121585618 | A | DOMxCHS | 0.04213 | 0.1129 | -0.1791 | 0.2633 | 0.3732 | 0.709 | 1 | 0.001404 | 0.02143 |
| 12 | 12:121619764:C:T | 121619764 | T | DOMxCHS | -0.2463 | 0.6622 | -1.544 | 1.052 | -0.3719 | 0.71 | 1 | 0.000826 | 0.001648 |
| 12 | 12:121600741:G:A | 121600741 | A | DOMxCHS | -0.2461 | 0.6623 | -1.544 | 1.052 | -0.3716 | 0.7102 | 1 | 0.000825 | 0.001099 |
| 12 | 12:121581565:G:T | 121581565 | T | DOMxCHS | -0.1413 | 0.3825 | -0.8911 | 0.6085 | -0.3694 | 0.7119 | 1 | 0.000552 | 0.004945 |
| 12 | 12:121626605:C:A | 121626605 | A | DOMxCHS | -0.05314 | 0.1446 | -0.3365 | 0.2302 | -0.3675 | 0.7133 | 1 | 0.000826 | 0.002198 |
| 12 | rs117096891:121588434:G:A | 121588434 | A | DOMxCHS | 0.02548 | 0.07199 | -0.1156 | 0.1666 | 0.3539 | 0.7234 | 1 | 0.002238 | 0.01813 |
| 12 | rs190576548:121561514:T:C | 121561514 | C | DOMxCHS | -0.01468 | 0.0419 | -0.09681 | 0.06745 | -0.3504 | 0.7261 | 1 | 0.003057 | 0.01154 |
| 12 | rs17512249:121564117:A:G | 121564117 | G | DOMxCHS | 0.003475 | 0.0102 | -0.01652 | 0.02347 | 0.3406 | 0.7335 | 1 | 0.08657 | 0.00989 |
| 12 | rs188949296:121599218:G:T | 121599218 | T | DOMxCHS | 0.02308 | 0.06886 | -0.1119 | 0.158 | 0.3351 | 0.7376 | 1 | 0.002769 | 0.007692 |
| 12 | rs117464188:121600727:G:A | 121600727 | A | DOMxCHS | 0.03228 | 0.09636 | -0.1566 | 0.2211 | 0.335 | 0.7377 | 1 | 0.002238 | 0.01813 |
| 12 | rs373438932:121615397:A:G | 121615397 | G | DOMxCHS | 0.01252 | 0.03745 | -0.06088 | 0.08592 | 0.3343 | 0.7382 | 1 | 0.006392 | 0.01154 |
| 12 | rs28360458:121622024:G:A | 121622024 | A | DOMxCHS | -0.00335 | 0.01009 | -0.02313 | 0.01643 | -0.3317 | 0.7401 | 0.4815 | 0.09203 | 0 |
| 12 | rs74892325:121633282:C:T | 121633282 | T | DOMxCHS | -0.00385 | 0.01224 | -0.02783 | 0.02014 | -0.3143 | 0.7534 | 0.8295 | 0.05771 | 0.00989 |
| 12 | exm1044235 | 121605408 | G | DOMxCHS | 0.0135 | 0.04356 | -0.07188 | 0.09888 | 0.3099 | 0.7567 | 1 | 0.003022 | 0 |
| 12 | rs208296:121600953:G:A | 121600953 | A | DOMxCHS | 0.00254 | 0.008218 | -0.01357 | 0.01865 | 0.3091 | 0.7573 | 0.1182 | 0.2977 | 0.1308 |
| 12 | rs187833144:121567182:G:A | 121567182 | A | DOMxCHS | 0.01887 | 0.06109 | -0.1009 | 0.1386 | 0.3088 | 0.7575 | 1 | 0.003583 | 0.003297 |
| 12 | 12:121608343:C:T | 121608343 | T | DOMxCHS | 0.04536 | 0.1531 | -0.2547 | 0.3455 | 0.2962 | 0.7671 | 1 | 0.00055 | 0.00055 |

| 12 | rs1186055:121600529:C:A | 121600529 | A | DOMxCHS | -0.00249 | 0.008496 | -0.01914 | 0.01416 | -0.293 | 0.7696 | 0.8891 | 0.2426 | 0.1665 |
| --- | --- | --- | --- | --- | --- | --- | --- | --- | --- | --- | --- | --- | --- |
| 12 | 12:121602778:G:A | 121602778 | A | DOMxCHS | -0.07322 | 0.2551 | -0.5733 | 0.4268 | -0.287 | 0.7742 | 1 | 0.000551 | 0.002747 |
| 12 | exm1044230 | 121605373 | A | DOMxCHS | -0.00558 | 0.0202 | -0.04516 | 0.03401 | -0.2762 | 0.7824 | 1 | 0.01869 | 0.00055 |
| 12 | rs113760750:121618715:C:T | 121618715 | T | DOMxCHS | 0.003487 | 0.01265 | -0.02131 | 0.02828 | 0.2757 | 0.7828 | 1 | 0.05451 | 0.007143 |
| 12 | exm1044228 | 121605355 | A | DOMxCHS | 0.002194 | 0.007989 | -0.01346 | 0.01785 | 0.2746 | 0.7836 | 0.4678 | 0.2308 | 0 |
| 12 | rs11065456:121586827:G:A | 121586827 | A | DOMxCHS | 0.1047 | 0.3824 | -0.6449 | 0.8542 | 0.2737 | 0.7844 | 1 | 0.00055 | 0.001648 |
| 12 | rs56050444:121601175:A:G | 121601175 | G | DOMxCHS | 0.002185 | 0.008222 | -0.01393 | 0.0183 | 0.2657 | 0.7905 | 0.2346 | 0.252 | 0.1126 |
| 12 | exm1044225 | 121605337 | A | DOMxCHS | -0.04827 | 0.1831 | -0.4071 | 0.3106 | -0.2637 | 0.7921 | 1 | 0.000824 | 0 |
| 12 | rs28360442:121570940:G:C | 121570940 | C | DOMxCHS | 0.005049 | 0.01957 | -0.0333 | 0.0434 | 0.258 | 0.7964 | 0.3997 | 0.02914 | 0.03846 |
| 12 | rs2857585:121600802:G:A | 121600802 | A | DOMxCHS | 0.003983 | 0.01572 | -0.02683 | 0.0348 | 0.2533 | 0.8 | 0.5797 | 0.0485 | 0.139 |
| 12 | rs112265934:121621699:C:T | 121621699 | T | DOMxCHS | 0.003166 | 0.01265 | -0.02164 | 0.02797 | 0.2502 | 0.8025 | 1 | 0.05426 | 0.007692 |
| 12 | rs73220272:121614227:T:C | 121614227 | C | DOMxCHS | -0.00263 | 0.01053 | -0.02327 | 0.01801 | -0.2498 | 0.8028 | 0.6557 | 0.08669 | 0.004945 |
| 12 | rs34481602:121616464:G:A | 121616464 | A | DOMxCHS | -0.00263 | 0.01053 | -0.02327 | 0.01801 | -0.2498 | 0.8028 | 0.6557 | 0.08669 | 0.004945 |
| 12 | rs66850672:121619834:T:C | 121619834 | C | DOMxCHS | -0.00263 | 0.01053 | -0.02327 | 0.01801 | -0.2498 | 0.8028 | 0.6557 | 0.08669 | 0.004945 |
| 12 | rs12815078:121622007:A:G | 121622007 | G | DOMxCHS | -0.00263 | 0.01053 | -0.02326 | 0.01801 | -0.2495 | 0.803 | 0.6558 | 0.08674 | 0.005495 |
| 12 | rs28360441:121569476:G:A | 121569476 | A | DOMxCHS | -0.04794 | 0.1988 | -0.4376 | 0.3417 | -0.2412 | 0.8094 | 1 | 0.00194 | 0.008791 |
| 12 | rs11065472:121626221:G:A | 121626221 | A | DOMxCHS | 0.0019 | 0.00802 | -0.01382 | 0.01762 | 0.2369 | 0.8128 | 0.01718 | 0.2668 | 0.03736 |
| 12 | rs2686385:121628027:T:C | 121628027 | C | DOMxCHS | 0.001895 | 0.008057 | -0.0139 | 0.01769 | 0.2352 | 0.8141 | 0.003284 | 0.2258 | 0.03516 |
| 12 | rs373833096:121634207:G:A | 121634207 | A | DOMxCHS | 0.008988 | 0.03822 | -0.06591 | 0.08389 | 0.2352 | 0.8141 | 1 | 0.006121 | 0.01264 |
| 12 | rs191927757:121572297:C:T | 121572297 | T | DOMxCHS | 0.01115 | 0.0475 | -0.08195 | 0.1042 | 0.2347 | 0.8145 | 1 | 0.005254 | 0.006593 |
| 12 | rs191002770:121609030:C:T | 121609030 | T | DOMxCHS | 0.02511 | 0.1089 | -0.1883 | 0.2385 | 0.2306 | 0.8177 | 1 | 0.000824 | 0 |
| 12 | rs117050530:121610131:G:A | 121610131 | A | DOMxCHS | -0.00979 | 0.04321 | -0.09448 | 0.0749 | -0.2266 | 0.8208 | 1 | 0.003052 | 0.00989 |
| 12 | rs2393799:121570012:C:T | 121570012 | T | DOMxCHS | -0.0018 | 0.007965 | -0.01741 | 0.01381 | -0.2258 | 0.8214 | 0.0749 | 0.2376 | 0.04011 |
| 12 | rs169631:121583751:C:G | 121583751 | G | DOMxCHS | -0.00175 | 0.007767 | -0.01697 | 0.01347 | -0.2253 | 0.8218 | 0.01282 | 0.274 | 0.01154 |
| 12 | exm1044175 | 121570899 | A | DOMxCHS | -0.00855 | 0.03968 | -0.08633 | 0.06923 | -0.2154 | 0.8295 | 1 | 0.009341 | 0 |
| 12 | rs73220279:121624309:C:T | 121624309 | T | DOMxCHS | 0.00227 | 0.01076 | -0.01882 | 0.02336 | 0.2109 | 0.833 | 0.2313 | 0.0856 | 0.008242 |
| 12 | rs2686372:121605826:A:G | 121605826 | G | DOMxCHS | 0.002602 | 0.01269 | -0.02227 | 0.02747 | 0.2051 | 0.8375 | 1 | 0.05451 | 0.01209 |
| 12 | rs28969482:121622887:A:C | 121622887 | C | DOMxCHS | -0.00429 | 0.02092 | -0.04529 | 0.03671 | -0.2051 | 0.8375 | 1 | 0.01736 | 0.002747 |
| 12 | 12:121562742:C:T | 121562742 | T | DOMxCHS | -0.06788 | 0.3324 | -0.7194 | 0.5836 | -0.2042 | 0.8382 | 1 | 0.000832 | 0.00989 |
| 12 | rs208293:121600180:C:T | 121600180 | T | DOMxCHS | 0.001625 | 0.008029 | -0.01411 | 0.01736 | 0.2023 | 0.8397 | 0.02516 | 0.2744 | 0.05385 |
| 12 | rs183283:121582240:G:A | 121582240 | A | DOMxCHS | -0.00156 | 0.007765 | -0.01677 | 0.01366 | -0.2004 | 0.8412 | 0.01279 | 0.2742 | 0.01099 |
| 12 | 12:121571915:G:A | 121571915 | A | DOMxCHS | 0.07549 | 0.3828 | -0.6748 | 0.8258 | 0.1972 | 0.8437 | 1 | 0.00055 | 0.001099 |
| 12 | rs185394597:121599393:G:A | 121599393 | A | DOMxCHS | -0.00765 | 0.03981 | -0.08568 | 0.07037 | -0.1922 | 0.8476 | 1 | 0.008545 | 0.003297 |
| 12 | rs77593826:121577218:C:T | 121577218 | T | DOMxCHS | -0.1385 | 0.764 | -1.636 | 1.359 | -0.1812 | 0.8562 | 1 | 0.000553 | 0.007143 |
| 12 | rs189989:121590354:T:C | 121590354 | C | DOMxCHS | -0.00131 | 0.007789 | -0.01657 | 0.01396 | -0.168 | 0.8666 | 0.007858 | 0.2756 | 0.01209 |
| 12 | rs7978398:121601455:T:C | 121601455 | C | DOMxCHS | 0.001313 | 0.008189 | -0.01474 | 0.01736 | 0.1604 | 0.8726 | 0.693 | 0.2495 | 0.09505 |
| 12 | exm1044240 | 121613229 | A | DOMxCHS | 0.003158 | 0.02092 | -0.03785 | 0.04416 | 0.1509 | 0.8801 | 0.03427 | 0.01291 | 0 |
| 12 | 12:121622571:A:C | 121622571 | C | DOMxCHS | 0.03759 | 0.2548 | -0.4618 | 0.537 | 0.1475 | 0.8827 | 1 | 0.00055 | 0.001099 |
| 12 | rs56297690:121618568:C:G | 121618568 | G | DOMxCHS | -0.00151 | 0.01052 | -0.02212 | 0.0191 | -0.144 | 0.8856 | 0.3725 | 0.08702 | 0.005495 |
| 12 | rs112924491:121600381:G:C | 121600381 | C | DOMxCHS | 0.0365 | 0.2552 | -0.4638 | 0.5368 | 0.143 | 0.8863 | 1 | 0.000551 | 0.002747 |
| 12 | rs77594915:121627068:C:T | 121627068 | T | DOMxCHS | 0.001778 | 0.01286 | -0.02343 | 0.02699 | 0.1382 | 0.8901 | 1 | 0.05361 | 0.01099 |
| 12 | rs149037578:121591986:G:C | 121591986 | C | DOMxCHS | -0.00503 | 0.03679 | -0.07713 | 0.06707 | -0.1367 | 0.8913 | 0.06784 | 0.006347 | 0.004396 |
| 12 | rs169633:121594684:T:C | 121594684 | C | DOMxCHS | -0.00106 | 0.007794 | -0.01634 | 0.01421 | -0.1364 | 0.8915 | 0.007795 | 0.2762 | 0.01538 |
| 12 | rs169632:121588545:T:C | 121588545 | C | DOMxCHS | -0.00106 | 0.007889 | -0.01652 | 0.01441 | -0.1341 | 0.8934 | 0.01478 | 0.2754 | 0.02527 |
| 12 | rs191426006:121565719:G:C | 121565719 | C | DOMxCHS | -0.00545 | 0.04073 | -0.08527 | 0.07437 | -0.1339 | 0.8935 | 1 | 0.003872 | 0.006593 |
| 12 | rs208287:121587353:T:C | 121587353 | C | DOMxCHS | -0.00101 | 0.007759 | -0.01621 | 0.0142 | -0.1295 | 0.897 | 0.01099 | 0.2739 | 0.004945 |
| 12 | rs74567697:121614677:G:T | 121614677 | T | DOMxCHS | 0.003288 | 0.02575 | -0.04718 | 0.05375 | 0.1277 | 0.8984 | 1 | 0.01114 | 0.01374 |
| 12 | rs11065466:121604748:G:A | 121604748 | A | DOMxCHS | -0.00102 | 0.008177 | -0.01704 | 0.01501 | -0.1242 | 0.9011 | 0.4148 | 0.2632 | 0.07088 |
| 12 | rs1718161:121627458:A:G | 121627458 | G | DOMxCHS | -0.00105 | 0.008535 | -0.01778 | 0.01568 | -0.1227 | 0.9024 | 0.04128 | 0.1689 | 0.03571 |
| 12 | exm1044200 | 121600238 | A | DOMxCHS | 0.002265 | 0.01876 | -0.0345 | 0.03903 | 0.1207 | 0.9039 | 1 | 0.0206 | 0 |
| 12 | rs185266542:121595347:C:G | 121595347 | G | DOMxCHS | -0.00483 | 0.04041 | -0.08403 | 0.07438 | -0.1194 | 0.905 | 1 | 0.004146 | 0.006044 |
| 12 | rs151030336:121634266:G:A | 121634266 | A | DOMxCHS | -0.00599 | 0.05109 | -0.1061 | 0.09415 | -0.1172 | 0.9067 | 1 | 0.000827 | 0.003846 |
| 12 | rs7309886:121563267:T:A | 121563267 | A | DOMxCHS | -0.00089 | 0.007845 | -0.01627 | 0.01449 | -0.1136 | 0.9096 | 0.01176 | 0.25 | 0.01538 |
| 12 | 12:121607794:C:G | 121607794 | G | DOMxCHS | 0.08369 | 0.765 | -1.416 | 1.583 | 0.1094 | 0.9129 | 1 | 0.000551 | 0.002747 |
| 12 | rs671684:121577778:G:A | 121577778 | A | DOMxCHS | -0.00082 | 0.007726 | -0.01596 | 0.01432 | -0.1064 | 0.9153 | 0.01071 | 0.2738 | 0.01154 |
| 12 | rs112956506:121629032:G:A | 121629032 | A | DOMxCHS | 0.001336 | 0.01286 | -0.02387 | 0.02654 | 0.1039 | 0.9173 | 1 | 0.05435 | 0.009341 |
| 12 | rs3751145:121630562:G:A | 121630562 | A | DOMxCHS | 0.001264 | 0.01286 | -0.02395 | 0.02648 | 0.09823 | 0.9218 | 1 | 0.05441 | 0.01044 |
| 12 | rs113810203:121627872:G:A | 121627872 | A | DOMxCHS | 0.001224 | 0.01287 | -0.02399 | 0.02644 | 0.09512 | 0.9242 | 1 | 0.05411 | 0.00989 |
| 12 | rs75009692:121628267:C:T | 121628267 | T | DOMxCHS | -0.00122 | 0.01298 | -0.02667 | 0.02423 | -0.09396 | 0.9252 | 0.8128 | 0.05209 | 0.01374 |
| 12 | rs200485794:121615289:G:C | 121615289 | C | DOMxCHS | 0.01685 | 0.1911 | -0.3577 | 0.3914 | 0.08817 | 0.9298 | 1 | 0.00055 | 0.001648 |
| 12 | rs208280:121561052:C:T | 121561052 | C | DOMxCHS | -0.00066 | 0.007832 | -0.01601 | 0.01469 | -0.08457 | 0.9326 | 0.01417 | 0.251 | 0.01484 |
| 12 | rs208290:121594056:G:A | 121594056 | A | DOMxCHS | -0.00064 | 0.007803 | -0.01593 | 0.01466 | -0.08177 | 0.9348 | 0.009046 | 0.2747 | 0.01484 |
| 12 | rs685019:121570476:A:G | 121570476 | A | DOMxCHS | -0.00061 | 0.007871 | -0.01604 | 0.01482 | -0.07759 | 0.9382 | 0.0233 | 0.2506 | 0.01758 |
| 12 | exm1044202 | 121600253 | A | DOMxCHS | 0.000651 | 0.008505 | -0.01602 | 0.01732 | 0.07656 | 0.939 | 0.1568 | 0.4577 | 0 |
| 12 | rs369906318:121575925:C:T | 121575925 | T | DOMxCHS | -0.00197 | 0.02582 | -0.05258 | 0.04863 | -0.07639 | 0.9391 | 1 | 0.01253 | 0.01319 |
| 12 | rs591874:121571465:C:A | 121571465 | C | DOMxCHS | -0.00057 | 0.00784 | -0.01594 | 0.01479 | -0.07287 | 0.9419 | 0.01995 | 0.2508 | 0.01648 |
| 12 | rs208284:121563016:C:T | 121563016 | C | DOMxCHS | -0.00057 | 0.007834 | -0.01592 | 0.01479 | -0.07253 | 0.9422 | 0.01416 | 0.2508 | 0.01429 |
| 12 | rs116952507:121620306:C:T | 121620306 | T | DOMxCHS | 0.001442 | 0.02054 | -0.03882 | 0.04171 | 0.07018 | 0.9441 | 0.1839 | 0.02915 | 0.02912 |
| 12 | rs1183296:121573636:A:G | 121573636 | A | DOMxCHS | -0.00054 | 0.007872 | -0.01597 | 0.01488 | -0.06913 | 0.9449 | 0.01092 | 0.2476 | 0.02363 |
| 12 | 12:121582094:G:A | 121582094 | A | DOMxCHS | 0.005895 | 0.09571 | -0.1817 | 0.1935 | 0.06159 | 0.9509 | 1 | 0.000552 | 0.003846 |
| 12 | rs501167:121574089:A:G | 121574089 | A | DOMxCHS | -0.00047 | 0.007848 | -0.01585 | 0.01491 | -0.05995 | 0.9522 | 0.01947 | 0.2504 | 0.02033 |
| 12 | rs117629432:121561406:C:T | 121561406 | T | DOMxCHS | -0.00181 | 0.03525 | -0.07089 | 0.06727 | -0.05137 | 0.959 | 1 | 0.007255 | 0.0533 |
| 12 | rs76966772:121561420:T:C | 121561420 | C | DOMxCHS | 0.02403 | 0.4682 | -0.8937 | 0.9417 | 0.05132 | 0.9591 | 1 | 0.001657 | 0.004945 |
| 12 | rs78954593:121563143:C:A | 121563143 | A | DOMxCHS | 0.02403 | 0.4682 | -0.8937 | 0.9417 | 0.05132 | 0.9591 | 1 | 0.001657 | 0.004945 |
| 12 | rs79890533:121601021:C:T | 121601021 | T | DOMxCHS | 0.000875 | 0.01787 | -0.03415 | 0.0359 | 0.04896 | 0.961 | 1 | 0.03083 | 0.01978 |
| 12 | 12:121613915:C:T | 121613915 | T | DOMxCHS | -0.01641 | 0.3827 | -0.7664 | 0.7336 | -0.04287 | 0.9658 | 1 | 0.000552 | 0.004945 |
| 12 | 12:121607127:T:C | 121607127 | C | DOMxCHS | 0.005988 | 0.1531 | -0.2941 | 0.3061 | 0.03911 | 0.9688 | 1 | 0.00055 | 0.00055 |
| 12 | rs117786959:121563169:G:A | 121563169 | A | DOMxCHS | 0.001288 | 0.03719 | -0.0716 | 0.07417 | 0.03464 | 0.9724 | 1 | 0.006579 | 0.03956 |
| 12 | rs656612:121576652:C:A | 121576652 | C | DOMxCHS | -0.00027 | 0.007761 | -0.01548 | 0.01494 | -0.03443 | 0.9725 | 0.008552 | 0.2718 | 0.01758 |
| 12 | rs117606904:121608442:G:A | 121608442 | A | DOMxCHS | -0.00035 | 0.01045 | -0.02083 | 0.02013 | -0.03363 | 0.9732 | 0.3052 | 0.0885 | 0.006593 |
| 12 | rs73220266:121608663:G:C | 121608663 | C | DOMxCHS | -0.00035 | 0.01045 | -0.02083 | 0.02013 | -0.03363 | 0.9732 | 0.3052 | 0.0885 | 0.006593 |
| 12 | rs56067598:121610687:G:C | 121610687 | C | DOMxCHS | -0.00035 | 0.01045 | -0.02083 | 0.02013 | -0.03363 | 0.9732 | 0.3052 | 0.0885 | 0.006593 |
| 12 | rs500930:121573997:T:C | 121573997 | T | DOMxCHS | 0.000189 | 0.007875 | -0.01525 | 0.01562 | 0.02402 | 0.9808 | 0.01656 | 0.2544 | 0.03791 |
| 12 | rs11065462:121601433:T:C | 121601433 | C | DOMxCHS | -0.00013 | 0.00851 | -0.01681 | 0.01655 | -0.01503 | 0.988 | 0.6198 | 0.231 | 0.133 |
| 12 | rs11065463:121601436:A:G | 121601436 | G | DOMxCHS | -0.00013 | 0.00851 | -0.01681 | 0.01655 | -0.01503 | 0.988 | 0.6198 | 0.231 | 0.133 |
| 12 | 12:121610910:G:A | 121610910 | A | DOMxCHS | 0.000845 | 0.06605 | -0.1286 | 0.1303 | 0.0128 | 0.9898 | 1 | 0.001651 | 0.001648 |

| 12 | rs2686364:121634093:A:T | 121634093 | T | DOMxCHS | 0.000146 | 0.02109 | -0.04119 | 0.04148 | 0.006915 | 0.9945 | 1 | 0.01875 | 0.07692 |
| --- | --- | --- | --- | --- | --- | --- | --- | --- | --- | --- | --- | --- | --- |
| 12 | rs113773372:121560692:C:T | 121560692 | T | DOMxCHS | NA | NA | NA | NA | NA | NA | 1 | 0.000275 | 0.001648 |
| 12 | 12:121560834:C:T | 121560834 | T | DOMxCHS | NA | NA | NA | NA | NA | NA | 1 | 0.000276 | 0.004396 |
| 12 | rs113108100:121561706:C:T | 121561706 | 0 | DOMxCHS | NA | NA | NA | NA | NA | NA | 1 | 0 | 0 |
| 12 | rs208281:121561937:T:C | 121561937 | T | DOMxCHS | NA | NA | NA | NA | NA | NA | 1 | 0.000275 | 0.001099 |
| 12 | 12:121562146:G:A | 121562146 | A | DOMxCHS | NA | NA | NA | NA | NA | NA | 1 | 0.000275 | 0 |
| 12 | rs190100846:121562199:G:T | 121562199 | T | DOMxCHS | NA | NA | NA | NA | NA | NA | 1 | 0.000275 | 0.00055 |
| 12 | rs75618867:121562299:T:C | 121562299 | 0 | DOMxCHS | NA | NA | NA | NA | NA | NA | 1 | 0 | 0 |
| 12 | 12:121563156:T:A | 121563156 | A | DOMxCHS | NA | NA | NA | NA | NA | NA | 1 | 0.000275 | 0.00055 |
| 12 | rs208285:121563357:G:A | 121563357 | G | DOMxCHS | NA | NA | NA | NA | NA | NA | 1 | 0.000275 | 0.001099 |
| 12 | rs79868114:121564060:A:G | 121564060 | G | DOMxCHS | NA | NA | NA | NA | NA | NA | 1 | 0.000275 | 0 |
| 12 | rs566496:121564325:T:A | 121564325 | T | DOMxCHS | NA | NA | NA | NA | NA | NA | 1 | 0.000275 | 0.001099 |
| 12 | 12:121564448:T:G | 121564448 | G | DOMxCHS | NA | NA | NA | NA | NA | NA | 1 | 0.000275 | 0 |
| 12 | rs2708094:121565100:C:T | 121565100 | C | DOMxCHS | NA | NA | NA | NA | NA | NA | 1 | 0.000275 | 0.001099 |
| 12 | rs148325187:121566007:A:C | 121566007 | C | DOMxCHS | NA | NA | NA | NA | NA | NA | 1 | 0.000275 | 0.001648 |
| 12 | rs57942857:121566545:G:A | 121566545 | A | DOMxCHS | NA | NA | NA | NA | NA | NA | 1 | 0.000275 | 0.002198 |
| 12 | rs192805735:121567021:G:A | 121567021 | A | DOMxCHS | NA | NA | NA | NA | NA | NA | 1 | 0.000275 | 0.001648 |
| 12 | 12:121567045:G:C | 121567045 | C | DOMxCHS | NA | NA | NA | NA | NA | NA | 1 | 0.000275 | 0.002198 |
| 12 | 12:121567319:G:C | 121567319 | C | DOMxCHS | NA | NA | NA | NA | NA | NA | 1 | 0.000275 | 0.001648 |
| 12 | rs9805054:121567370:G:A | 121567370 | A | DOMxCHS | NA | NA | NA | NA | NA | NA | 1 | 0.000275 | 0 |
| 12 | rs113663757:121567871:G:A | 121567871 | A | DOMxCHS | NA | NA | NA | NA | NA | NA | 1 | 0.000275 | 0.002198 |
| 12 | rs653682:121568200:T:A | 121568200 | T | DOMxCHS | NA | NA | NA | NA | NA | NA | 1 | 0.000275 | 0.001099 |
| 12 | 12:121568489:A:G | 121568489 | G | DOMxCHS | NA | NA | NA | NA | NA | NA | 1 | 0.000276 | 0.002747 |
| 12 | rs111291922:121568882:G:C | 121568882 | C | DOMxCHS | NA | NA | NA | NA | NA | NA | 1 | 0.000275 | 0.002198 |
| 12 | rs113841817:121568905:C:T | 121568905 | T | DOMxCHS | NA | NA | NA | NA | NA | NA | 1 | 0.000275 | 0.002198 |
| 12 | rs657647:121569003:G:A | 121569003 | G | DOMxCHS | NA | NA | NA | NA | NA | NA | 1 | 0.000275 | 0.001099 |
| 12 | rs77850380:121569455:T:C | 121569455 | C | DOMxCHS | NA | NA | NA | NA | NA | NA | 1 | 0.000275 | 0.002198 |
| 12 | 12:121571163:T:C | 121571163 | C | DOMxCHS | NA | NA | NA | NA | NA | NA | 1 | 0.000276 | 0.004945 |
| 12 | rs185272507:121571371:C:G | 121571371 | G | DOMxCHS | NA | NA | NA | NA | NA | NA | 1 | 0.000275 | 0.00055 |
| 12 | rs148807698:121571539:C:T | 121571539 | T | DOMxCHS | NA | NA | NA | NA | NA | NA | 1 | 0.00055 | 0.00055 |
| 12 | rs114701429:121572136:A:G | 121572136 | G | DOMxCHS | NA | NA | NA | NA | NA | NA | 1 | 0.000275 | 0.001099 |
| 12 | rs112260002:121572345:T:C | 121572345 | C | DOMxCHS | NA | NA | NA | NA | NA | NA | 1 | 0.000275 | 0.002198 |
| 12 | rs182397877:121572623:G:C | 121572623 | C | DOMxCHS | NA | NA | NA | NA | NA | NA | 1 | 0.000276 | 0.003297 |
| 12 | rs149332019:121572909:C:T | 121572909 | T | DOMxCHS | NA | NA | NA | NA | NA | NA | 1 | 0.000275 | 0.002198 |
| 12 | rs2859405:121572910:A:G | 121572910 | A | DOMxCHS | NA | NA | NA | NA | NA | NA | 1 | 0.000275 | 0.001099 |
| 12 | rs2859406:121573090:A:G | 121573090 | A | DOMxCHS | NA | NA | NA | NA | NA | NA | 1 | 0.000275 | 0.001099 |
| 12 | rs145984668:121574335:G:A | 121574335 | A | DOMxCHS | NA | NA | NA | NA | NA | NA | 1 | 0.000275 | 0 |
| 12 | rs141624289:121574472:T:A | 121574472 | 0 | DOMxCHS | NA | NA | NA | NA | NA | NA | 1 | 0 | 0 |
| 12 | 12:121574537:C:A | 121574537 | A | DOMxCHS | NA | NA | NA | NA | NA | NA | 1 | 0.000275 | 0.00055 |
| 12 | rs112542334:121574574:C:T | 121574574 | 0 | DOMxCHS | NA | NA | NA | NA | NA | NA | 1 | 0 | 0.002198 |
| 12 | rs190004504:121574905:G:C | 121574905 | C | DOMxCHS | NA | NA | NA | NA | NA | NA | 1 | 0.000275 | 0.00055 |
| 12 | rs11065447:121574982:G:A | 121574982 | A | DOMxCHS | NA | NA | NA | NA | NA | NA | 1 | 0.000275 | 0 |
| 12 | rs78430762:121576444:G:A | 121576444 | 0 | DOMxCHS | NA | NA | NA | NA | NA | NA | 1 | 0 | 0.004945 |
| 12 | rs144700257:121576447:G:A | 121576447 | A | DOMxCHS | NA | NA | NA | NA | NA | NA | 1 | 0.000275 | 0.00055 |
| 12 | rs375299566:121576586:C:T | 121576586 | 0 | DOMxCHS | NA | NA | NA | NA | NA | NA | 1 | 0 | 0 |
| 12 | rs111935452:121576893:T:C | 121576893 | C | DOMxCHS | NA | NA | NA | NA | NA | NA | 1 | 0.000275 | 0.002198 |
| 12 | 12:121577496:C:T | 121577496 | T | DOMxCHS | NA | NA | NA | NA | NA | NA | 1 | 0.000276 | 0.003297 |
| 12 | rs111667701:121577634:T:C | 121577634 | 0 | DOMxCHS | NA | NA | NA | NA | NA | NA | 1 | 0 | 0.001099 |
| 12 | rs11065449:121577703:G:T | 121577703 | T | DOMxCHS | NA | NA | NA | NA | NA | NA | 1 | 0.000275 | 0 |
| 12 | 12:121577772:C:T | 121577772 | T | DOMxCHS | NA | NA | NA | NA | NA | NA | 1 | 0.000275 | 0 |
| 12 | rs672632:121577951:G:A | 121577951 | A | DOMxCHS | NA | NA | NA | NA | NA | NA | 1 | 0.000275 | 0.001648 |
| 12 | rs144824453:121578160:T:G | 121578160 | G | DOMxCHS | NA | NA | NA | NA | NA | NA | 1 | 0.000276 | 0.003297 |
| 12 | rs12831943:121578619:G:A | 121578619 | A | DOMxCHS | NA | NA | NA | NA | NA | NA | 1 | 0.000281 | 0.02308 |
| 12 | 12:121578806:T:A | 121578806 | A | DOMxCHS | NA | NA | NA | NA | NA | NA | 1 | 0.000275 | 0 |
| 12 | rs376938041:121579823:G:A | 121579823 | 0 | DOMxCHS | NA | NA | NA | NA | NA | NA | 1 | 0 | 0 |
| 12 | rs139431200:121580110:A:G | 121580110 | G | DOMxCHS | NA | NA | NA | NA | NA | NA | 1 | 0.000275 | 0 |
| 12 | rs138006429:121582526:C:A | 121582526 | 0 | DOMxCHS | NA | NA | NA | NA | NA | NA | 1 | 0 | 0 |
| 12 | rs149481328:121582722:G:A | 121582722 | A | DOMxCHS | NA | NA | NA | NA | NA | NA | 1 | 0.00055 | 0.00055 |
| 12 | rs79126683:121583081:C:T | 121583081 | T | DOMxCHS | NA | NA | NA | NA | NA | NA | 1 | 0.000275 | 0 |
| 12 | rs112725652:121584338:C:G | 121584338 | G | DOMxCHS | NA | NA | NA | NA | NA | NA | 1 | 0.000275 | 0.002198 |
| 12 | rs150877869:121584371:A:G | 121584371 | G | DOMxCHS | NA | NA | NA | NA | NA | NA | 1 | 0.000275 | 0.002198 |
| 12 | rs75787674:121584374:G:A | 121584374 | A | DOMxCHS | NA | NA | NA | NA | NA | NA | 1 | 0.000276 | 0.002747 |
| 12 | rs111664562:121584398:C:T | 121584398 | T | DOMxCHS | NA | NA | NA | NA | NA | NA | 1 | 0.000275 | 0.002198 |
| 12 | rs77372307:121584484:C:G | 121584484 | G | DOMxCHS | NA | NA | NA | NA | NA | NA | 1 | 0.000275 | 0.002198 |
| 12 | 12:121586099:C:T | 121586099 | T | DOMxCHS | NA | NA | NA | NA | NA | NA | 1 | 0.000275 | 0.00055 |
| 12 | 12:121586352:T:C | 121586352 | C | DOMxCHS | NA | NA | NA | NA | NA | NA | 1 | 0.000275 | 0.002198 |
| 12 | rs372563892:121586371:C:T | 121586371 | 0 | DOMxCHS | NA | NA | NA | NA | NA | NA | 1 | 0 | 0 |
| 12 | 12:121586550:C:G | 121586550 | G | DOMxCHS | NA | NA | NA | NA | NA | NA | 1 | 0.000826 | 0.002747 |
| 12 | 12:121586738:G:C | 121586738 | C | DOMxCHS | NA | NA | NA | NA | NA | NA | 1 | 0.000275 | 0 |
| 12 | rs208286:121586900:C:T | 121586900 | T | DOMxCHS | NA | NA | NA | NA | NA | NA | 1 | 0.000275 | 0.00055 |
| 12 | 12:121587979:C:T | 121587979 | T | DOMxCHS | NA | NA | NA | NA | NA | NA | 1 | 0.000275 | 0 |
| 12 | 12:121588181:T:A | 121588181 | A | DOMxCHS | NA | NA | NA | NA | NA | NA | 1 | 0.000275 | 0 |
| 12 | 12:121589928:G:A | 121589928 | A | DOMxCHS | NA | NA | NA | NA | NA | NA | 1 | 0.000275 | 0 |
| 12 | rs147822402:121591302:G:A | 121591302 | A | DOMxCHS | NA | NA | NA | NA | NA | NA | 1 | 0.000276 | 0.003297 |
| 12 | 12:121591345:G:A | 121591345 | A | DOMxCHS | NA | NA | NA | NA | NA | NA | 1 | 0.000276 | 0.002747 |
| 12 | 12:121591952:G:A | 121591952 | A | DOMxCHS | NA | NA | NA | NA | NA | NA | 1 | 0.000275 | 0.001099 |
| 12 | rs201754454:121592715:C:T | 121592715 | T | DOMxCHS | NA | NA | NA | NA | NA | NA | 1 | 0.000275 | 0.002198 |
| 12 | rs142113769:121593021:C:T | 121593021 | T | DOMxCHS | NA | NA | NA | NA | NA | NA | 1 | 0.000275 | 0 |
| 12 | rs192897139:121594136:C:T | 121594136 | T | DOMxCHS | NA | NA | NA | NA | NA | NA | 1 | 0.000275 | 0.001648 |
| 12 | rs147435951:121596175:C:A | 121596175 | A | DOMxCHS | NA | NA | NA | NA | NA | NA | 1 | 0.000275 | 0.002198 |
| 12 | 12:121597259:C:T | 121597259 | T | DOMxCHS | NA | NA | NA | NA | NA | NA | 1 | 0.000275 | 0 |
| 12 | rs75309104:121597698:C:T | 121597698 | T | DOMxCHS | NA | NA | NA | NA | NA | NA | 1 | 0.000275 | 0.001099 |
| 12 | variant.27844 | 121598738 | A | DOMxCHS | NA | NA | NA | NA | NA | NA | 1 | 0.000275 | 0 |
| 12 | rs115913335:121599826:C:T | 121599826 | T | DOMxCHS | NA | NA | NA | NA | NA | NA | 1 | 0.000276 | 0.002747 |
| 12 | rs201551247:121600361:C:T | 121600361 | T | DOMxCHS | NA | NA | NA | NA | NA | NA | 1 | 0.000275 | 0.00055 |
| 12 | 12:121600434:C:T | 121600434 | T | DOMxCHS | NA | NA | NA | NA | NA | NA | 1 | 0.000275 | 0 |

| 12 | rs169634:121600731:A:G | 121600731 | G | DOMxCHS | NA | NA | NA | NA | NA | NA | 1 | 0.000275 | 0.00055 |
| --- | --- | --- | --- | --- | --- | --- | --- | --- | --- | --- | --- | --- | --- |
| 12 | 12:121600901:G:A | 121600901 | A | DOMxCHS | NA | NA | NA | NA | NA | NA | 1 | 0.000275 | 0 |
| 12 | rs149130739:121601226:T:C | 121601226 | C | DOMxCHS | NA | NA | NA | NA | NA | NA | 1 | 0.000276 | 0.005495 |
| 12 | rs190081467:121601669:G:A | 121601669 | A | DOMxCHS | NA | NA | NA | NA | NA | NA | 1 | 0.000276 | 0.002747 |
| 12 | rs208297:121602027:C:T | 121602027 | T | DOMxCHS | NA | NA | NA | NA | NA | NA | 1 | 0.000275 | 0 |
| 12 | 12:121603027:G:A | 121603027 | A | DOMxCHS | NA | NA | NA | NA | NA | NA | 1 | 0.000275 | 0.001648 |
| 12 | 12:121604931:C:T | 121604931 | T | DOMxCHS | NA | NA | NA | NA | NA | NA | 1 | 0.000275 | 0.002198 |
| 12 | rs12299020:121604950:A:G | 121604950 | G | DOMxCHS | NA | NA | NA | NA | NA | NA | 1 | 0.000276 | 0.006044 |
| 12 | rs77414815:121605095:G:A | 121605095 | A | DOMxCHS | NA | NA | NA | NA | NA | NA | 1 | 0.000276 | 0.005495 |
| 12 | rs189238545:121605547:T:G | 121605547 | G | DOMxCHS | NA | NA | NA | NA | NA | NA | 1 | 0.000275 | 0.001099 |
| 12 | rs113142210:121605590:A:G | 121605590 | 0 | DOMxCHS | NA | NA | NA | NA | NA | NA | 1 | 0 | 0 |
| 12 | 12:121605702:G:A | 121605702 | 0 | DOMxCHS | NA | NA | NA | NA | NA | NA | 1 | 0 | 0.00055 |
| 12 | rs56896451:121605867:C:T | 121605867 | 0 | DOMxCHS | NA | NA | NA | NA | NA | NA | 1 | 0 | 0 |
| 12 | rs78892156:121606621:C:T | 121606621 | 0 | DOMxCHS | NA | NA | NA | NA | NA | NA | 1 | 0 | 0.00055 |
| 12 | 12:121607509:G:C | 121607509 | C | DOMxCHS | NA | NA | NA | NA | NA | NA | 1 | 0.000275 | 0 |
| 12 | rs186224346:121608027:A:G | 121608027 | G | DOMxCHS | NA | NA | NA | NA | NA | NA | 1 | 0.000275 | 0.001648 |
| 12 | rs114703019:121608135:G:A | 121608135 | 0 | DOMxCHS | NA | NA | NA | NA | NA | NA | 1 | 0 | 0 |
| 12 | rs1653615:121608316:G:A | 121608316 | A | DOMxCHS | NA | NA | NA | NA | NA | NA | 1 | 0.000275 | 0.00055 |
| 12 | rs111251427:121608317:C:G | 121608317 | 0 | DOMxCHS | NA | NA | NA | NA | NA | NA | 1 | 0 | 0 |
| 12 | rs112272761:121609123:C:T | 121609123 | 0 | DOMxCHS | NA | NA | NA | NA | NA | NA | 1 | 0 | 0 |
| 12 | rs372503527:121609226:G:A | 121609226 | A | DOMxCHS | NA | NA | NA | NA | NA | NA | 1 | 0.000276 | 0.004396 |
| 12 | rs145745616:121609229:G:A | 121609229 | A | DOMxCHS | NA | NA | NA | NA | NA | NA | 1 | 0.000275 | 0.002198 |
| 12 | rs78802962:121609388:T:G | 121609388 | 0 | DOMxCHS | NA | NA | NA | NA | NA | NA | 1 | 0 | 0 |
| 12 | 12:121609584:C:G | 121609584 | G | DOMxCHS | NA | NA | NA | NA | NA | NA | 1 | 0.000275 | 0.00055 |
| 12 | 12:121609726:G:A | 121609726 | A | DOMxCHS | NA | NA | NA | NA | NA | NA | 1 | 0.000276 | 0.002747 |
| 12 | rs112108667:121610035:G:C | 121610035 | 0 | DOMxCHS | NA | NA | NA | NA | NA | NA | 1 | 0 | 0 |
| 12 | rs192770237:121610581:G:A | 121610581 | A | DOMxCHS | NA | NA | NA | NA | NA | NA | 1 | 0.000276 | 0.005495 |
| 12 | rs79396321:121610884:T:C | 121610884 | 0 | DOMxCHS | NA | NA | NA | NA | NA | NA | 1 | 0 | 0 |
| 12 | 12:121610949:C:G | 121610949 | G | DOMxCHS | NA | NA | NA | NA | NA | NA | 1 | 0.000276 | 0.003297 |
| 12 | rs112199676:121611914:G:A | 121611914 | 0 | DOMxCHS | NA | NA | NA | NA | NA | NA | 1 | 0 | 0 |
| 12 | rs78021965:121612632:C:T | 121612632 | 0 | DOMxCHS | NA | NA | NA | NA | NA | NA | 1 | 0 | 0 |
| 12 | 12:121612977:G:T | 121612977 | 0 | DOMxCHS | NA | NA | NA | NA | NA | NA | 1 | 0 | 0 |
| 12 | rs74550515:121613310:C:G | 121613310 | G | DOMxCHS | NA | NA | NA | NA | NA | NA | 1 | 0.000275 | 0 |
| 12 | 12:121614249:G:T | 121614249 | T | DOMxCHS | NA | NA | NA | NA | NA | NA | 1 | 0.000275 | 0 |
| 12 | rs113128175:121614384:T:A | 121614384 | 0 | DOMxCHS | NA | NA | NA | NA | NA | NA | 1 | 0 | 0 |
| 12 | rs73220273:121614923:G:A | 121614923 | A | DOMxCHS | NA | NA | NA | NA | NA | NA | 1 | 0.000278 | 0.01154 |
| 12 | rs114333980:121617671:C:T | 121617671 | T | DOMxCHS | NA | NA | NA | NA | NA | NA | 1 | 0.000275 | 0 |
| 12 | rs138845503:121617781:C:T | 121617781 | T | DOMxCHS | NA | NA | NA | NA | NA | NA | 1 | 0.000275 | 0 |
| 12 | 12:121617981:C:T | 121617981 | T | DOMxCHS | NA | NA | NA | NA | NA | NA | 1 | 0.000275 | 0.001648 |
| 12 | rs1718110:121618003:C:T | 121618003 | T | DOMxCHS | NA | NA | NA | NA | NA | NA | 1 | 0.000275 | 0.00055 |
| 12 | rs2567998:121618141:A:C | 121618141 | C | DOMxCHS | NA | NA | NA | NA | NA | NA | 1 | 0.000275 | 0.00055 |
| 12 | rs113550058:121618564:G:A | 121618564 | 0 | DOMxCHS | NA | NA | NA | NA | NA | NA | 1 | 0 | 0 |
| 12 | rs149014451:121618756:G:C | 121618756 | C | DOMxCHS | NA | NA | NA | NA | NA | NA | 1 | 0.000275 | 0.00055 |
| 12 | rs193108219:121618983:C:T | 121618983 | T | DOMxCHS | NA | NA | NA | NA | NA | NA | 1 | 0.000276 | 0.003846 |
| 12 | rs76045059:121619054:A:G | 121619054 | G | DOMxCHS | NA | NA | NA | NA | NA | NA | 1 | 0.000277 | 0.009341 |
| 12 | rs2568000:121619529:G:A | 121619529 | A | DOMxCHS | NA | NA | NA | NA | NA | NA | 1 | 0.000275 | 0.00055 |
| 12 | 12:121620070:T:G | 121620070 | G | DOMxCHS | NA | NA | NA | NA | NA | NA | 1 | 0.000275 | 0.00055 |
| 12 | rs2686381:121621529:C:T | 121621529 | T | DOMxCHS | NA | NA | NA | NA | NA | NA | 1 | 0.000275 | 0.00055 |
| 12 | rs2686382:121621668:C:G | 121621668 | G | DOMxCHS | NA | NA | NA | NA | NA | NA | 1 | 0.000275 | 0.00055 |
| 12 | rs2686383:121621669:C:A | 121621669 | A | DOMxCHS | NA | NA | NA | NA | NA | NA | 1 | 0.000275 | 0.00055 |
| 12 | rs202019673:121622082:A:G | 121622082 | G | DOMxCHS | NA | NA | NA | NA | NA | NA | 1 | 0.000275 | 0.002198 |
| 12 | exm1044261 | 121622115 | 0 | DOMxCHS | NA | NA | NA | NA | NA | NA | 1 | 0 | 0 |
| 12 | exm1756219 | 121622380 | 0 | DOMxCHS | NA | NA | NA | NA | NA | NA | 1 | 0 | 0 |
| 12 | exm1756220 | 121622408 | A | DOMxCHS | NA | NA | NA | NA | NA | NA | 1 | 0.000275 | 0 |
| 12 | exm1044290 | 121622562 | A | DOMxCHS | NA | NA | NA | NA | NA | NA | 1 | 0.000275 | 0 |
| 12 | rs2568004:121622675:G:A | 121622675 | A | DOMxCHS | NA | NA | NA | NA | NA | NA | 1 | 0.000275 | 0.00055 |
| 12 | rs200082733:121623195:C:A | 121623195 | A | DOMxCHS | NA | NA | NA | NA | NA | NA | 1 | 0.000275 | 0 |
| 12 | rs188985951:121623388:C:T | 121623388 | T | DOMxCHS | NA | NA | NA | NA | NA | NA | 1 | 0.000275 | 0 |
| 12 | rs199946271:121623552:C:T | 121623552 | T | DOMxCHS | NA | NA | NA | NA | NA | NA | 1 | 0.000276 | 0.003846 |
| 12 | rs202085453:121623567:T:C | 121623567 | C | DOMxCHS | NA | NA | NA | NA | NA | NA | 1 | 0.000275 | 0.001648 |
| 12 | 12:121623953:C:T | 121623953 | T | DOMxCHS | NA | NA | NA | NA | NA | NA | 1 | 0.000276 | 0.004396 |
| 12 | rs1975123:121624355:A:G | 121624355 | G | DOMxCHS | NA | NA | NA | NA | NA | NA | 1 | 0.000275 | 0.00055 |
| 12 | 12:121624755:C:T | 121624755 | T | DOMxCHS | NA | NA | NA | NA | NA | NA | 1 | 0.000275 | 0.00055 |
| 12 | rs143207508:121625015:T:A | 121625015 | A | DOMxCHS | NA | NA | NA | NA | NA | NA | 1 | 0.000275 | 0.001099 |
| 12 | rs79757926:121625515:G:A | 121625515 | A | DOMxCHS | NA | NA | NA | NA | NA | NA | 1 | 0.000275 | 0 |
| 12 | rs190920223:121627302:C:T | 121627302 | T | DOMxCHS | NA | NA | NA | NA | NA | NA | 1 | 0.000275 | 0 |
| 12 | 12:121627669:C:T | 121627669 | T | DOMxCHS | NA | NA | NA | NA | NA | NA | 1 | 0.000275 | 0.001099 |
| 12 | rs138545160:121628458:G:C | 121628458 | C | DOMxCHS | NA | NA | NA | NA | NA | NA | 1 | 0.000275 | 0.001099 |
| 12 | rs192517925:121628938:C:T | 121628938 | T | DOMxCHS | NA | NA | NA | NA | NA | NA | 1 | 0.000276 | 0.003846 |
| 12 | rs142943384:121629453:A:G | 121629453 | G | DOMxCHS | NA | NA | NA | NA | NA | NA | 1 | 0.000275 | 0.001099 |
| 12 | rs114603231:121629860:G:T | 121629860 | 0 | DOMxCHS | NA | NA | NA | NA | NA | NA | 1 | 0 | 0 |
| 12 | 12:121630086:C:T | 121630086 | T | DOMxCHS | NA | NA | NA | NA | NA | NA | 1 | 0.000275 | 0 |
| 12 | 12:121630795:G:A | 121630795 | A | DOMxCHS | NA | NA | NA | NA | NA | NA | 1 | 0.000275 | 0.00055 |
| 12 | rs145352774:121631406:G:T | 121631406 | T | DOMxCHS | NA | NA | NA | NA | NA | NA | 1 | 0.000275 | 0 |
| 12 | rs186120742:121631688:G:A | 121631688 | A | DOMxCHS | NA | NA | NA | NA | NA | NA | 1 | 0.000275 | 0 |
| 12 | rs116052323:121632072:G:A | 121632072 | A | DOMxCHS | NA | NA | NA | NA | NA | NA | 1 | 0.000275 | 0 |
| 12 | 12:121632254:G:A | 121632254 | A | DOMxCHS | NA | NA | NA | NA | NA | NA | 1 | 0.000275 | 0 |
| 12 | 12:121632689:A:G | 121632689 | G | DOMxCHS | NA | NA | NA | NA | NA | NA | 1 | 0.000276 | 0.005495 |
| 12 | rs74355017:121632803:T:C | 121632803 | C | DOMxCHS | NA | NA | NA | NA | NA | NA | 1 | 0.000277 | 0.007143 |
| 12 | rs2686366:121633623:C:T | 121633623 | T | DOMxCHS | NA | NA | NA | NA | NA | NA | 1 | 0.000275 | 0.001099 |
| 12 | rs2686365:121633783:C:T | 121633783 | T | DOMxCHS | NA | NA | NA | NA | NA | NA | 1 | 0.000275 | 0.001099 |

S2 Table. In silico functional characterisation of SNPs in the significant clumps

| S2 Table SNP function prediction | | | |  |  |  |  |  |  |  |  |  |  |  |  |  |  |  |  |  |  |  |
| --- | --- | --- | --- | --- | --- | --- | --- | --- | --- | --- | --- | --- | --- | --- | --- | --- | --- | --- | --- | --- | --- | --- |
| No. | rs | Chromo- some | Position | Allele | TFBS | Splicin g(site) | Splicin  g(ESE  or ESS) | Splicing (abolish domain) | miRNA (miRan da) | miRNA (Sanger  ) | nsSNP | Stop Codo n | Polyphen | SNPs3D  (svm profile) | SNPs3D  (svm structure  ) | Reg Potential | Conservatio n | Nearby Gene | Distance (bp) | Allel e | European | CEU |
| 1 | **rs67881993** | 12 | 1.22E+08 | G/T | -- | -- | -- | -- | -- | -- | -- | -- | -- | -- | -- | NA | 0 | LOC105370032 | -- | G | -- | -- |
| 2 | rs111259202 | 12 | 1.22E+08 | G/A | -- | -- | -- | -- | -- | -- | -- | -- | -- | -- | -- | NA | 0 | P2RX7 \|\| LOC105370032 | -- | G | -- | -- |
| 3 | rs12810503 | 12 | 1.2E+08 | C/G | Y | -- | -- | -- | -- | -- | -- | -- | -- | -- | -- | NA | 0 | OASL\|\|P2RX7 | -89735\|\|-4163 | -- | -- | -- |
| 4 | rs12813980 | 12 | 1.2E+08 | A/C | -- | -- | -- | -- | -- | -- | -- | -- | -- | -- | -- | 0 | 0 | P2RX7 | 27809\|\|25389 | C | 0.933 | 0.93 |
| 5 | rs12816966 | 12 | 1.2E+08 | A/G | Y | -- | -- | -- | -- | -- | -- | -- | -- | -- | -- | NA | 0 | OASL\|\|P2RX7 | -90890\|\|-3008 | -- | -- | -- |
| 6 | rs12820593 | 12 | 1.2E+08 | A/G | -- | -- | -- | -- | -- | -- | -- | -- | -- | -- | -- | 0 | 0.022 | P2RX7 | 13259\|\|39939 | A | -- | -- |
| 7 | rs12824585 | 12 | 1.2E+08 | A/C | -- | -- | -- | -- | -- | -- | -- | -- | -- | -- | -- | 0 | 0.183 | OASL\|\|P2RX7 | -85088\|\|-8810 | -- | -- | -- |
| 8 | rs12830584 | 12 | 1.2E+08 | A/T | -- | -- | -- | -- | -- | -- | -- | -- | -- | -- | -- | NA | 0 | P2RX7 | 10983\|\|42215 | -- | -- | -- |
| 9 | rs12832396 | 12 | 1.2E+08 | C/T | -- | -- | -- | -- | -- | -- | -- | -- | -- | -- | -- | NA | 0 | P2RX7 | 11003\|\|42195 | T | 0.963 | 0.96 |
| 10 | rs139825828 | 12 | 1.22E+08 | A/T | -- | -- | -- | -- | -- | -- | -- | -- | -- | -- | -- | - | - | LOC105370032 | - | A | -- | -- |
| 11 | rs146637843 | 12 | 1.21E+08 | G/A | -- | -- | -- | -- | -- | -- | -- | -- | -- | -- | -- | - | - | LOC105370032 | - | G | -- | -- |
| 12 | rs17434640 | 12 | 1.2E+08 | A/G | -- | -- | -- | -- | -- | -- | -- | -- | -- | -- | -- | 0 | 0 | P2RX7 | 7772\|\|45426 | G | 0.913 | 0.94 |
| 13 | Rs17525809 /  exm1044184 | 12 | 1.2E+08 | C/T | -- | -- | Y | -- | -- | -- | Y | -- | benign | -- | -- | 0.01125 | 0 | P2RX7 | 22011\|\|31187 | T | 0.913 | 0.93 |
| 14 | rs28969465 | 12 | 1.2E+08 | A/G | Y | -- | -- | -- | -- | -- | -- | -- | -- | -- | -- | NA | 0 | OASL\|\|P2RX7 | -92116\|\|-1782 | -- | -- | -- |
| 15 | rs28969469 | 12 | 1.2E+08 | A/G | Y | -- | -- | -- | -- | -- | -- | -- | -- | -- | -- | 0.23362 | 0 | OASL\|\|P2RX7 | -93367\|\|-531 | -- | -- | -- |
| 16 | rs34185850 | 12 | 1.2E+08 | C/T | -- | -- | -- | -- | -- | -- | -- | -- | -- | -- | -- | 0.00789 | 0 | P2RX7 | 7673\|\|45525 | -- | -- | -- |
| 17 | rs34480856 | 12 | 1.2E+08 | A/G | -- | -- | -- | -- | -- | -- | -- | -- | -- | -- | -- | NA | 0.002 | P2RX7 | 14312\|\|38886 | -- | -- | -- |
| 18 | rs34572498 | 12 | 1.2E+08 | C/G | -- | -- | -- | -- | -- | -- | -- | -- | -- | -- | -- | 0 | 0.006 | P2RX7 | 30133\|\|23065 | -- | -- | -- |
| 19 | rs35018823 | 12 | 1.2E+08 | A/G | -- | -- | -- | -- | -- | -- | -- | -- | -- | -- | -- | 0 | 0.007 | P2RX7 | 25461\|\|27737 | -- | -- | -- |
| 20 | rs35076950 | 12 | 1.2E+08 | C/T | -- | -- | -- | -- | -- | -- | -- | -- | -- | -- | -- | NA | 0 | P2RX7 | 3732\|\|49466 | -- | -- | -- |
| 21 | rs35237790 | 12 | 1.2E+08 | A/G | -- | -- | -- | -- | -- | -- | -- | -- | -- | -- | -- | 0 | 0.008 | P2RX7 | 24508\|\|28690 | -- | -- | -- |
| 22 | rs35605477 | 12 | 1.2E+08 | C/T | -- | -- | -- | -- | -- | -- | -- | -- | -- | -- | -- | 0.04845 | 0 | P2RX7 | 16356\|\|36842 | -- | -- | -- |
| 23 | rs494986 | 12 | 1.2E+08 | C/A | Y | -- | -- | -- | -- | -- | -- | -- | -- | -- | -- | 0.03698 | 0 | OASL\|\|P2RX7 | -93156\|\|-742 | C | -- | -- |
| 24 | rs657172 | 12 | 1.2E+08 | A/G | Y | -- | -- | -- | -- | -- | -- | -- | -- | -- | -- | NA | 0 | OASL\|\|P2RX7 | -92094\|\|-1804 | -- | -- | -- |
| 25 | rs670541 | 12 | 1.2E+08 | C/T | Y | -- | -- | -- | -- | -- | -- | -- | -- | -- | -- | 0 | 0 | OASL\|\|P2RX7 | -92854\|\|-1044 | C | -- | 0.93 |
| 26 | rs67691679 | 12 | 1.22E+08 | C/A,T | -- | -- | -- | -- | -- | -- | -- | -- | -- | -- | -- | NA | 0 | P2RX7 \|\| LOC105370032 | -- | C | -- | -- |
| 27 | rs71454680 | 12 | 1.22E+08 | G/A | -- | -- | -- | -- | -- | -- | -- | -- | -- | -- | -- | NA | 0 | P2RX7 \|\| LOC105370032 | -- | G | -- | -- |
| 28 | rs73218241 | 12 | 1.22E+08 | G/A | -- | -- | -- | -- | -- | -- | -- | -- | -- | -- | -- | NA | 0 | P2RX7 \|\| LOC105370032 | -- | G | -- | -- |
| 29 | rs78473339 | 12 | 1.22E+08 | G/C | -- | -- | -- | -- | -- | -- | -- | -- | -- | -- | -- | -- | -- | P2RX7\|\|LOC105370032 | -- | G | -- | -- |

Page 1

explanation

| TFBS | nsSNP | Splicing |
| --- | --- | --- |
| transcriptional regulation by affecting transcription factor binding sites (TFBS) activity; TFBS Prediction  If a non-coding SNP is located at a transcription factor-binding site (TFBS) of a gene, then it may affect the level, location, or timing of gene expression. We predicted such SNPs according to the procedure described in Xu and Taylor (submitted). | SNPs in protein- coding regions that can cause amino acid change (non- synonymous coding SNPs, nsSNP). | SNPs that are located at 2 base pair of intron-exon junction region, exonic splicing enhancer (ESE), or exonic splicing silencer (ESS) may disrupt splicing activity and cause alternative splicing. We predict SNPs whose alternative alleles may affect splicing using the methods detailed in Xu and Taylor (submitted). |

<https://snpinfo.niehs.nih.gov/snpinfo/guide.html>

Page 2

clinvar

|  | clinvar | Has function | dbSNP | GWAS cat. | Ref | Litvar |  |
| --- | --- | --- | --- | --- | --- | --- | --- |
| **rs67881993** | - | - | Genic downstream transcript variant,intron variant | - |  |  | lead SNP |
| rs12824585 | **-** | **conservation = 0.183** | **Intron variant, genic downstream transcript variant** | - |  |  |  |
| rs12810503 | - | TFBS | Genic downstream transcript variant,intron variant | - |  |  |  |
| rs12816966 | - | TFBS | Genic downstream transcript variant,intron variant | - |  |  |  |
| rs139825828 | - | - | Genic downstream transcript variant,intron variant | - |  |  |  |
| rs146637843 | - | - | Genic downstream transcript variant,intron variant | - |  |  |  |
| rs657172 | - | TFBS | Upstream transcript variant,intron variant,2KB upstream variant, genic downstream transcript variant | - |  |  |  |
| rs28969465 | - | TFBS | 2KB_upstream_variant,genic_downstream_transcript_vari ant,upstream_transcript_variant,intron_variant | - |  |  |  |
| rs670541 | yes, but not related | TFBS | upstream_transcript_variant,intron_variant,2KB_upstream  _variant,genic_downstream_transcript_variant | [- .](https://www.ncbi.nlm.nih.gov/pmc/articles/PMC4265416/) | [ncbi.nlm.nih.gov/pmc/articles/PMC4265416/](https://www.ncbi.nlm.nih.gov/pmc/articles/PMC4265416/) | |  |
| rs494986 | - | TFBS, reg.pot = 0.036984 | upstream_transcript_variant,intron_variant,2KB_upstream  _variant,genic_downstream_transcript_variant | - |  |  |  |
| rs28969469 | - | TFBS, reg.pot = 0.233619 | 2KB_upstream_variant,genic_downstream_transcript_vari ant,upstream_transcript_variant,intron_variant | - |  |  |  |
| rs67691679 | - | - | genic_downstream_transcript_variant,intron_variant | - |  |  |  |
| rs35076950 | - | no | genic_downstream_transcript_variant,intron_variant | - |  |  |  |
| rs34185850 | - | reg.pot = 0.007891 | genic_downstream_transcript_variant,intron_variant | - |  |  |  |
| rs12830584 | - | no | genic_downstream_transcript_variant,intron_variant | - |  |  |  |
| rs12832396 | - | no | genic_downstream_transcript_variant,intron_variant | - |  |  |  |
| rs12820593 | - | conservation = 0.022 | genic_downstream_transcript_variant,intron_variant | - |  |  |  |
| rs34480856 | - | conservation = 0.002 | genic_downstream_transcript_variant,intron_variant | - |  |  |  |
| rs35605477 | - | reg.pot = 0.048451 | genic_downstream_transcript_variant,intron_variant | - |  |  |  |
| rs111259202 | - | - | genic_downstream_transcript_variant,intron_variant | - |  |  |  |
| rs71454680 | - | - | genic_downstream_transcript_variant,intron_variant | - |  |  |  |
| rs73218241 | - | - | genic_downstream_transcript_variant,intron_variant | - |  |  |  |
| **7525809 (exm10441** | yes, but only one related (multiple sclerosis) | Splicing(ESE or ESS), Polyphen (Benign), nsSNP | **coding_sequence_variant,5_prime_UTR_variant,**non_c oding_transcript_variant,intron_variant,missense_variant,g enic_downstream_transcript_variant | - | rm=rs17525809&sort=date&ac=yes  ,  https://[www.ncbi.nlm.nih.gov/pm](http://www.ncbi.nlm.nih.gov/pm) | [https://www.ncbi.nlm.ni](https://www.ncbi.nlm.nih.gov/CBBresearch/Lu/Demo/LitVar/%23!?query=rs17525809) [h.gov/CBBresearch/Lu/](https://www.ncbi.nlm.nih.gov/CBBresearch/Lu/Demo/LitVar/%23!?query=rs17525809) [Demo/LitVar/#!?query=](https://www.ncbi.nlm.nih.gov/CBBresearch/Lu/Demo/LitVar/%23!?query=rs17525809) [rs17525809](https://www.ncbi.nlm.nih.gov/CBBresearch/Lu/Demo/LitVar/%23!?query=rs17525809) |  |
| rs35237790 | - | conservation = 0.008 | genic_downstream_transcript_variant,intron_variant | - |  |  |  |
| rs35018823 | - | conservation = 0.007 | genic_downstream_transcript_variant,intron_variant | - |  |  |  |
| rs12813980 | - | no | genic_downstream_transcript_variant,intron_variant | - |  |  |  |
| rs78473339 | - | - | genic_downstream_transcript_variant,intron_variant,downs tream_transcript_variant | - |  |  |  |
| rs34572498 | - | conservation = 0.006 | intron_variant | - | - | - |  |
| rs17434640 | - | - | intron_variant,genic_downstream_transcript_variant | - | - | - |  |

Page 3
